# Supplementary material for: Effect of community health education on mothers’ knowledge of obstetric danger signs and birth preparedness and complication readiness practices in southern Ethiopia: A cluster randomized controlled trial
Source: PLoS One. 2024 Nov 27;19(11):e0312267. doi: 10.1371/journal.pone.0312267 (PMC11602057; doi:10.1371/journal.pone.0312267)
Supplement: S2 File — (DOCX) [file pone.0312267.s002.docx]

**S2 File Table 1:** Description of study variables

| Study variables | Description |
| --- | --- |
| **Individual-level variables** | |
| Classification of obstetric danger signs (ODS) | Basic ODS are distinguished by women and categorized into three categories based on the time of occurrence. Vaginal bleeding, blurring of vision, high-grade fever, severe headaches, high blood pressure, and swelling of the hands and face are common ODS during pregnancy. The basic ODS during labor and childbirth are severe vaginal bleeding, convulsions, retained placenta > 30 minutes, and prolonged labor > 12 hours. The danger signs during the postpartum period include severe vaginal bleeding, high-grade fever, high blood pressure, severe headaches, and foul-smelling vaginal discharge [[1-3](#_ENREF_1)]. |
| Spontaneous knowledge | The ability of study subjects to identify or call an ODS without having data collectors read their names is known as spontaneous knowledge. During the interview, only true ODS that study participants spontaneously mentioned were recorded [[4](#_ENREF_4)]. |
| Component of birth preparedness and complication readiness (BPCR) plan | includes the following components: finding and communicating with a skilled birth attendant (SBA); identifying a close proper health facility (HF); saving money and material resources; preparing for transportation to a proper HF; and identifying and fixing compatible blood group donors |
| Distance from the HF | if a woman is reported to walk less than 30 minutes by foot to arrive at HFs or travel less than 5 km to reach the nearest HFs, the distance from the HF is assumed to be close to the HF [[5](#_ENREF_5),[6](#_ENREF_6)]. |
| Use of mass media | is calculated by combining whether a study subject listens to the radio, watches television, and reads the newspaper and is classified as "yes" if the participants is exposed to at least one of the three media and "no" otherwise [[7](#_ENREF_7),[8](#_ENREF_8)]. |
| Family size | is defined as the total number of people living in the household and is classified as small (fewer than 5) and large (more than 5) [[9](#_ENREF_9)]. |
| Formal education | is education that extends from primary to secondary and higher education and demands a careful and organized purpose that manifests itself in an official curriculum that is implemented according to a set calendar and timetable [[10](#_ENREF_10)]. |
| Women’s autonomy | a woman is regarded as autonomous if she can decide when and where to use MHS or on health care spending by herself alone or with her husband, and non-autonomous if she must rely on others based on her self-reporting [[11](#_ENREF_11)]. |
| **Community-level variables** | |
| Place of residence | classified as rural and urban |
| Community-level women's literacy | The aggregate value of community-level women's literacy was computed by calculating the percentage of women in the cluster who had at least a primary level of literacy using data from individual participants. If the percentage of women with at least a primary level of education is greater than 50%, the *kebeles* are classified as having a "high" concentration of literate women and otherwise as having a "low" concentration [[12](#_ENREF_12),[13](#_ENREF_13)]. |
| Community-level poverty | The aggregate value of community-level poverty was computed by using the proportion of households in the *kebele* in the poorer and poorest quintiles derived from individual participants' data. The *kebeles* are classified as having a "high" concentration of poverty if the proportion of households in the poorest and poorest quintiles exceeds 50% and as "low" otherwise [[12-14](#_ENREF_12)]. |
| Community-level mass media use | The aggregate value of community-level mass media use was computed by calculating the proportion of study participants who listen to the radio, watch television, and read the newspaper in a *kebele* based on individual participant data. The *kebeles* are classified as having a "high" concentration of mass media use if the proportion of study participants who used at least one social media is greater than 50% and "low" otherwise [[13](#_ENREF_13),[15](#_ENREF_15)]. |
| Distance from nearest HF | The aggregate value of community-level distance was computed by calculating the proportion of study respondents walking hours to the nearest HF in a *kebele* from individual participants' data. In the *kebeles*, a problem is classified as "not a big problem" if more than 50% of study participants reported it as "close" and as "a big problem" otherwise [[6](#_ENREF_6),[16](#_ENREF_16),[17](#_ENREF_17)]. |

**The wealth index computation procedure**

The multiple response variables were classified as binary (yes/no) and "I don't know" responses, which were frequently coded as 999 to zero (Table 3). Similarly, for continuous variables, the "I don't know" response and any missing value are frequently coded as 999 to zero [[18](#_ENREF_18)]. Simple frequency analysis was used to select factors that can distinguish between relatively "poor" and "rich" households. As a result, our PCA did not include any assets or variables that were owned by less than 5% or more than 95% of the people in the sample [[6](#_ENREF_6),[18](#_ENREF_18)]. Ultimately, five classes—the lowest, second-lowest, middle, second-highest, and highest—were created from the component factors or wealth index scores [[9](#_ENREF_9),[19](#_ENREF_19)]. The PCA method was used to calculate the wealth index [[18](#_ENREF_18),[19](#_ENREF_19)]. Before categorizing the component factor scores into wealth quintiles, all of PCA's basic assumptions were validated. We removed variables from PCA that did not meet the assumptions, such as the Kaiser-Meyer-Olkin (KMO) measure of sampling adequacy being less than 0.5, commonalities being less than 0.5, and variables with complex structure (high loading correlation >0.4 on more than one component [[19](#_ENREF_19),[20](#_ENREF_20)].

**S2 File Table 2:** Some of variables and given values to facilitate the computation of wealth index

| S.no | Variables | Given values |
| --- | --- | --- |
| 1 | Main source of drinking water | Improved: Piped water, tube well or borehole, protected well, protected spring = 1  Unimproved: Unprotected well, Unprotected spring, Lake/pond/stream/canal = 0 |
| 2 | Main source of water used for other purposes such as cooking and hand washing | Improved: Piped water, tube well or borehole, protected well, protected spring = 1  Unimproved: Unprotected well, Unprotected spring, Lake/pond/stream/canal, Surface water (River/dam) = 0 |
| 3 | Where is that water source located? | In own dwelling or yard/plot = 1  Elsewhere = 0 |
| 4 | Type of toilet facilities | Improved: comprise any non-shared toilet of the subsequent kinds: pour/flush toilets to septic tanks, piped sewer systems, and pit latrines; pit latrines with slabs; ventilated improved pit (VIP) latrines; and composting toilets = 1  Unimproved: Pit latrine without slab/open pit, bucket toilet and hanging toilet = 0 |
| 5 | Where is this toilet facility located? | In own dwelling or yard/plot = 1  Elsewhere = 0 |
| 6 | Type of fuel the household mainly use for cooking | Clean fuels include electricity, liquefied petroleum gas (LPG), natural gas, and biogas = 1  Solid fuels include coal, charcoal, wood, straw/shrub/grass, agricultural crops, and animal dung = 0 |
| 7 | Where is the cooking usually done? | In the house and outdors = 0  In a separate building = 1 |
| 8 | Who is the owner of the house? | Me = 1  Rental, family, and relative = 0 |
| 9 | Main material of the roof of the house | Natural roofing (no roof, mud, and sod) = 0  Rudimentary and finished roofing = 1 |
| 10 | Main material of the floor of the house | Natural floor (Earth/sand, dung) = 0  Rudimentary and finished floor = 1 |
| 11 | Main material of the wall of the house | Natural walls (no walls, cane/palm/trunks/bamboo/ree, dirt) = 0  Rudimentary and finished wall = 1 |
| 12 | All other categorical variables were considered as yes and no form | Yes = 1 and no =0 |
| 13 | All continuous variables were treated as continuous |  |
| 14 | “I don’t know” response often coded as 999 for categorical variables | 999 = 0 |
| 15 | “I don’t know” response and any missing value often coded as 999 to zero | 999 and missing value = 0 |

**HEI procedure**

The principal investigator developed the health education messages, which the research team members reviewed. Also, it was reviewed by a health education expert from Hawassa University. Then, the final version of health education messages was developed after several revisions. Subsequently, a female midwife and media experts were provided training about audio-recording the HEI. The midwife, a bachelor's degree holder in midwifery, narrated the developed script several times until all messages and sounds conveyed the intended messages in the context of the local language and culture. Then, the final version of the pre-recorded audio-based health education lecture was recorded. The audio recording was done by experts at a local media network studio (Sidama Media Network). Portable Bluetooth devices (Gepps's) were used to play the pre-recorded audio messages at every health education session.

The HEI was delivered for six months, twice a month; one session took one hour, and 12 sessions were held. One health education session consisted of critical messages on normal pregnancy and childbirth, ODS during pregnancy, delivery, and the postpartum period, the practice of BPCR, and the benefits of MHSU. The other actions executed by WDT leaders were motivating mothers and their families to attend HEI sessions. One hour was allocated for each session; 20 minutes were allowed for the pre-recorded audio-based lecture, and the remaining 40 minutes were used for questions and answers (discussion).

After each session, some women were selected to perform a role-play, an essential way to demonstrate important messages and share experiences. The role-play was done to repeat the information, enabling them to internalize the critical message. The women were also exposed to posters to strengthen the session message or cover the missed information during the audio lecture. Any questions, confusions, or misunderstandings during the session were recorded and reported to the midwife through HEWs if they could not explain adequately. HEWs residing in particular clusters were responsible for clarifying questions once per month. We recruited a midwife to provide a more detailed explanation if the issues raised were beyond the capacity of HEWs. The midwife, located outside the study area, was actively involved in clarifying the issues for HEWs and mothers via mobile phone during the next meeting at the end of the session for all women who attended the session. During the study period, a supervisor was assigned to each district to supervise the health education sessions monthly or more frequently if any potential problems were indicated for quality monitoring. Supervisors reported any concerns encountered by the principal investigator, like absences or disagreements between group members and WDT leaders. The principal investigator smoothly discussed and fixed the problems with group members, WDT leaders, and HEWs.

The present intervention differs from the routine intervention in several ways [[21-23](#_ENREF_21)]. Among these, the community-based nature of the present intervention includes all pregnant women in kebeles organized into small women's groups, each containing 15 pregnant women. The WDT leaders led 15 pregnant women and facilitated the intervention. A total of 38 groups of pregnant women were formed. Thus, the intervention included pregnant women from hard-to-reach areas in the kebele (decentralized in principle). The routine intervention is health post-based and provides health education for pregnant women who attend health posts. It does not consider pregnant women at home or in hard-to-reach areas. Second, our HEI was delivered twice a month, while the routine intervention was only once. The hypothesis is that frequent teaching will lead to increased knowledge and a birth preparedness plan. Third, our HEI is simple, straightforward, detailed in content, and supported by pre-recorded audio teaching material compared to the routine intervention (only lecture method). This audio-device-assisted health education is expected to provide standard or uniform information for all clusters to create a similar level of understanding. Fourth, this intervention detects pregnant women (before 12 weeks of pregnancy) via home-to-home visits and enrolls them. The routine intervention delivers health education to pregnant women regardless of gestational age (more likely at greater than 16 weeks). Thus, our method will likely increase the probability of women going through the continuum of care.

**S2 File Table 3:** Description of obstetric danger signs of the trial participants (N = 1,070)

| **Variables** | **Intervention group** | **Control group** | **Total** | **P- value** |
| --- | --- | --- | --- | --- |
|  | N (%) | N (%) | N (%) |  |
| **ODS during pregnancy** |  |  |  |  |
| Severe headache | 473 (87.6) | 395 (74.5) | 868 (81.1) | 0.001 |
| Excessive vaginal bleeding | 315 (58.3) | 306 (57.7) | 621 (58.0) | 0.843 |
| Blurry vision | 129 (23.9) | 87 (16.4) | 216 (20.2) | 0.002 |
| Reduced or absent foetal movement | 243 (45.0) | 90 (17.0) | 333 (31.1) | 0.001 |
| High blood pressure | 144 (26.7) | 59 (11.1) | 203 (19.0) | 0.001 |
| Oedema of the face | 147 (27.2) | 51 (9.6) | 198 (18.5) | 0.001 |
| Oedema of the hands and feet | 150 (27.8) | 66 (12.5) | 216 (20.2) | 0.001 |
| Convulsions | 222 (41.1) | 141 (26.6) | 363 (33.9) | 0.001 |
| Lower abdominal pain | 156 (28.9) | 63 (11.9) | 219 (20.5) | 0.001 |
| **ODS during childbirth** |  |  |  |  |
| Excessive vaginal bleeding | 509 (94.3) | 470 (88.7) | 979 (91.5) | 0.001 |
| Foul smelling discharge | 129 (23.9) | 86 (16.2) | 215 (20.1) | 0.002 |
| High grade fever | 208 (38.5) | 98 (18.5) | 306 (28.6) | 0.001 |
| Baby's hand or feet come first | 154 (28.5) | 66 (12.5) | 220 (20.6) | 0.001 |
| Baby bad position/ mal presentation | 148 (27.4) | 57 (10.8) | 250 (19.2) | 0.001 |
| Prolong labour (> l2 Hours) | 239 (44.3) | 156 (29.4) | 395 (36.9) | 0.001 |
| Retained placenta | 232 (43.0) | 199 (37.5) | 431 (40.3) | 0.071 |
| Torn uterus | 86 (15.9) | 40 (7.5) | 126 (11.8) | 0.001 |
| Prolapsed cord | 62 (11.5) | 25 (4.7) | 87 (8.1) | 0.001 |
| Cord around the neck | 112 (20.7) | 36 (6.8) | 148 (13.8) | 0.001 |
| Convulsions | 145 (26.9) | 50 (9.4) | 195 (18.2) | 0.001 |
| Perineal Tear | 246 (45.6) | 83 (15.7) | 329 (30.7) | 0.001 |
| **ODS during postpartum** |  |  |  |  |
| Excessive vaginal bleeding | 506 (93.7) | 457 (86.2) | 963 (90.0) | 0.001 |
| Foul smelling discharge | 146 (27.0) | 96 (18.1) | 242 (22.6) | 0.001 |
| High fever | 220 (40.7) | 107 (20.2) | 327 (30.6) | 0.001 |
| Tetanus | 163 (30.2) | 82 (15.5) | 245 (22.9) | 0.001 |
| Inverted nipples | 114 (21.1) | 52 (9.8) | 166 (15.5) | 0.001 |
| Retained placenta | 145 (26.9) | 58 (10.9) | 203 (19.0) | 0.001 |
| Severe abdominal pain | 282 (52.2) | 224 (42.3) | 506 (47.3) | 0.002 |
| Convulsions | 177 (32.8) | 127 (24.0) | 304 (28.4) | 0.002 |
| Engorged breasts | 268 (49.6) | 110 (20.8) | 378 (35.3) | 0.001 |
| **Overall** | 371 (68.7) | 192 (36.2) | 563 (52.6) | 0.001 |

**S2 File Table 4:** Description of birth preparedness and complication readiness practices of the trial participants (N = 1,070)

| Variables | Intervention group | Control group | Total | P- value |
| --- | --- | --- | --- | --- |
|  | N (%) | N (%) | N (%) |  |
| **BPCR practice** |  |  |  |  |
| Save money and material resources | 366 (97.1) | 266 (92.7) | 632 (95.2) | 0.009 |
| Identify proper closer HFs for childbirth | 357 (94.7) | 219 (76.3) | 576 (86.7) | 0.001 |
| Identify SBA for deliver | 361 (95.8) | 240 (83.6) | 601 (90.5) | 0.001 |
| Identify proper transport for delivery | 236 (62.6) | 107 (37.3) | 343 (51.7) | 0.001 |
| Identify and fixing the compatible blood group givers | 26 (6.9) | 16 (5.6) | 42 (6.3) | 0.488 |
| **Overall** | 347 (64.3) | 206 (38.9) | 553 (51.7) | 0.001 |

**S2 File Table 5:** Multilevel regression analysis result of a random intercept model for ODS knowledge and BPCR practice variation at cluster level in north zone of Sidama region, Ethiopia, 2023 (N = 1,070)

| Measure of variation | Model 1 (95% CI) | Model 2 (95% CI) | Model 3 (95% CI) | Model 4 (95% CI) |
| --- | --- | --- | --- | --- |
| **ODS knowledge related information** | | | | |
| Variance of intercept | 0.20 (0.06, 0.60) | 0.15 (0.04, 0.58) | 0.06 (0.01, 0.38) | 0.07 (0.01, 0.44) |
| ICC percentage | 27.46 (17.14-40.92) |  |  |  |
| Model fitness |  |  |  |  |
| Log-likelihood ratio | -898.98 | -869.88 | -890.41 | -864.85 |
| AIC | 1801.97 | 1775.78 | 1794.82 | 1775.71 |
| BIC | 1811.92 | 1865.33 | 1829.65 | 1790.15 |
| **BPCR practice related information** | | | | |
| Variance of intercept | 0.17 (0.04, 0.65) | 0.13 (0.02, 0.63) | 0.06 (0.01, 0.39) | 0.07 (0.01, 0.47) |
| ICC percentage | 38.78 (20.20, 74.46) |  |  |  |
| Model fitness |  |  |  |  |
| Log-likelihood ratio | -898.67 | -866.52 | -891.40 | -862.29 |
| AIC | 1801.34 | 1769.04 | 1796.81 | 1770.58 |
| BIC | 1811.30 | 1858.60 | 1831.64 | 1785.02 |

ICC: Intra-class correlation coefficient; AIC: Akaike information criteria; BIC: Bayesian information criteria; CI: confidence interval.

**Effect modification result of ODS knowledge**

We entered the interaction terms in the final model for women's occupation and intervention status, husband occupation and intervention status, mass media use and intervention status, wealth index and intervention status, model family training and intervention status to see if women's occupation, husband occupation, mass media use, wealth index and model family training modifies the effect of intervention. None of the interaction terms was statistically significant, implying the absence of a significant effect modification.

**Effect modification result of BPCR practice**

We entered the interaction terms in the final model for women's occupation and intervention status, husband occupation and intervention status, mass media use and intervention status, wealth index and intervention status, model family training and intervention status to see if women's occupation, husband occupation, mass media use, wealth index and model family training modifies the effect of intervention. None of the interaction terms was statistically significant, implying the absence of a significant effect modification.

**References**

1. JHPIEGO (2001) Maternal and Neonatal health (MNH) Program: Birth preparedness and complication readiness. A matrix of shared responsibilities. Maternal and Neonatal Health. 2001.

2. Making pregnancy and childbirth safer (2021) Policy brief, Washington, DC. USA world population reference bureau. 1997. Available: www. measuecommunication.org. Accessed June 2021.

3. Stevens RD (2000) Safe motherhood: an insight into maternal mortality in the developing world. Health Millions 26: 34-37.

4. Hailu D, Berhe H (2014) Knowledge about obstetric danger signs and associated factors among mothers in Tsegedie district, Tigray region, Ethiopia 2013: community based cross-sectional study. PLoS One 9: e83459.

5. Zegeye K GA, Melese T, (2014) The Role of Geographical Access in the Utilization of Institutional Delivery Service in Rural Jimma Horro District, Southwest Ethiopia . Primary Health Care 4: 150. doi:10.4172/2167- 1079.1000150.

6. Ahmed R SM, Abose S, Assefa B, Nuramo A, Alemu A, et al, (2022) Levels and associated factors of the maternal healthcare continuum in Hadiya zone, Southern Ethiopia: A multilevel analysis. PLoS ONE 17(10): e0275752. https://doi.org/10.1371/journal.pone.0275752

7. Singh P, Singh KK, Singh P (2021) Maternal health care service utilization among young married women in India, 1992–2016: trends and determinants. BMC Pregnancy and Childbirth 21: 122.

8. Fatema K (2019) "Impact of Mass Media on the Utilization of Maternal Healthcare Services in South Asia" (2019). Electronic Theses and Dissertations. 2031. https://digitalcommons.memphis.edu/etd/2031

9. Central Statistical Agency (CSA) [Ethiopia] and ICF (2019) Mini Ethiopia Demographic and Health Survey 2019: Key Indicators Report. Addis Ababa, Ethiopia, and Rockville, Maryland, USA. CSA and ICF. 2019.

10. Shudura E, Yoseph A, Tamiso A (2020) Utilization and predictors of maternal health care services among women of reproductive age in Hawassa University health and demographic surveillance system site, South Ethiopia: a Cross-Sectional Study. Advances in Public Health 2020: 1-10.

11. Asefa A, Gebremedhin S (2019) Mismatch between antenatal care attendance and institutional delivery in south Ethiopia: A multilevel analysis. 9: e024783.

12. Negash WD, Fetene SM (2022) Multilevel analysis of quality of antenatal care and associated factors among pregnant women in Ethiopia: a community based cross-sectional study. 12: e063426.

13. Huda TM, Chowdhury M, El Arifeen S, Dibley MJ (2019) Individual and community level factors associated with health facility delivery: A cross sectional multilevel analysis in Bangladesh. PLoS One 14: e0211113.

14. Liyew AM, Teshale AB (2020) Individual and community level factors associated with anemia among lactating mothers in Ethiopia using data from Ethiopian demographic and health survey, 2016; a multilevel analysis. BMC Public Health 20: 775.

15. Tessema ZT, Animut Y (2020) Spatial distribution and determinants of an optimal ANC visit among pregnant women in Ethiopia: further analysis of 2016 Ethiopia demographic health survey. BMC Pregnancy Childbirth 20: 137.

16. Chaka EE (2022) Multilevel analysis of continuation of maternal healthcare services utilization and its associated factors in Ethiopia: A cross-sectional study. PLOS Glob Public Health 2(5): e0000517. https://doi.org/10.1371/journal.pgph.0000517.

17. Zegeye B, Olorunsaiye CZ (2021) Individual/Household and Community-Level Factors Associated with Child Marriage in Mali: Evidence from Demographic and Health Survey. 2021: 5529375.

18. Fry K. FR, Chakraborty N.M, (2014) Measuring Equity with Nationally Representative Wealth Quintiles. Washington, DC: PSI. .

19. Vyas S, Kumaranayake L (2006) Constructing socio-economic status indices: how to use principal components analysis. Health Policy Plan 21: 459-468.

20. Principal component analysis Available online from https://slideplayer.com/slide/4238108/

21. Federal Democratic Republic of Ethiopia Ministry of Health (2015) Health Education, Advocacy and Community Mobilisation, Part 1. Blended Learning Module for the Health Extension Programme.

22. Minister of Health of Ethiopia (2013.) Prenant women monthly Conference Manual.

23. Save the Children International (SCI) Pregnant Women Conference Best Practice from Ethiopia. Available online from https://www.healthynewbornnetwork.org/hnn-content/uploads/Pregnant-Women-Conference.pdf.
