# Supplementary material for: Effect of community health education on mothers’ knowledge of obstetric danger signs and birth preparedness and complication readiness practices in southern Ethiopia: A cluster randomized controlled trial
Source: PLoS One. 2024 Nov 27;19(11):e0312267. doi: 10.1371/journal.pone.0312267 (PMC11602057; doi:10.1371/journal.pone.0312267)
Supplement: S3 File — (DOCX) [file pone.0312267.s003.docx]

1. **ANNEXES**

**Annex I: Information sheet**

**Hawassa University**

**College of Medicine and Health Sciences**

**School of Public Health**

**Project title: Improving maternal health service utilization among women of reproductive age in North Zone of Sidama National Regional State, Ethiopia**

Hello! My name is _------------------------------------------------------_. I am a data collector in a study being conducted about maternal health service utilization under the College of Medicine and Health Sciences of Hawassa University. We are conducting a study to improve maternal health service utilization in your community.

**Goal of the study**

The objective of this study is to improve maternal health service utilization. The study will be conducted for six months. After this baseline survey, selected mothers in some villages will be provided health education regarding maternal health topics.

At the end of intervention, we will test or check how well the education has assisted to improve MHSU in your villages by comparing to other villages that did not obtain education. The data obtained in this study will be valuable to prepare the education regarding maternal health topics.

**Significance of the study and method of participant selection**

The aim of this study is to generate information about the maternal health service utilization among women of reproductive age in North Zone of Sidama region. The study may help public health planners, stakeholders, policy makers, responsible body, and others to take actions based on the finding. The study comprises various intimate and private questions. We randomly selected you to help us by participating in this study as the data you offer us will helpful in averting maternal and child death and illness. Interview will take not more than 40 minutes.

**Risks and discomfort**

We assure you that there is no risk or harm in participation of this study. The study comprises various friendly and private questions. However, you may refuse to respond any of the queries if you feel uncomfortable.

**Benefits**

Your involvement will assist us to find out more regarding maternal health issue in the villages and to provide education to women in your communities. If this study revealed that the education is useful in increasing maternal health by improving proper use of MHS, the government and other stakeholders will be able to improve maternal health in other communities in this setting and other part of the country.

**Incentives**

There is no incentive or payment for participating in this research. Nonetheless, we will thank you very much for your involvement.

**Confidentiality**

All information will be kept confidentially. Name of a participant will not be written or specified. Your privacy will also be protected and no one shall know your response.

**Right to withdraw or refuse**

You have full right whether or not to participate in this study. You may respond to all questions or you may not answer to the questions you don’t want to or you may quit your participation totally at any time you want. You can ask any questions which is not clear for you. Moreover, we assure you that refusing to participate will not affect your future treatment at the health institution or elsewhere in any way.

**Who to contact**

You can ask any questions which are not clear for you now or later. If you wish to ask queries later, you may contact: Amanuel Yoseph, Hawassa University, Telephone: 0915683578.

**Annex II: Informed Consent**

I have been requested to take part in the research and read the foregoing information, or it has been read to me in the language I comprehend and understand all condition stated above. I have had the opportunity to ask questions about it and any questions I have been asked have been answered to my satisfaction. I consent voluntarily to be a participant in this study and understand that I have the right to withdraw from the interview at any time without in anyway affecting my right.

Are you willing to participate in this study?

Yes □ No □

If “Yes”, study participant’s ID……………………Signature…………….Date…………..

And proceed with the interview.

If “No” ...........thank you and end.

Name of the principal investigator: Amanuel Yoseph

Cell phone Number - +251-915683578 E mail: [amanuelyoseph45@gmail.com](mailto:amanuelyoseph45@gmail.com)

Name of interviewer___________________ Signature__________

Data of interviewer (Ethiopia calendar) _____/_____/_____

Result of interview: 1. Complete 2. Refused 3. Partially complete 4. Respondent not available

Cheeked by supervisor: Name______________ Signature_________ Data___/_____/____

**Annex III: English version questionnaire**

**Hawassa University**

**College of Medicine and Health Sciences**

**School of Public Health**

***Questionnaire designed to study the maternal health service utilization among women of reproductive age in rural North Zone of Sidama National Regional State, Ethiopia.***

**PART I: General Information**

01. Name of Woreda: ____________

02. Name of Kebele: ________________

03. Name of Gote __________________

04. House number ____________________

05. Individual Id __________________

06. Date of interview: ____________time interview started __________ ended at ____________

07. Respondent available on:

1. 1^st^ visit.

2. 2^nd^ visit.

3. 3^rd^ visit.

08: Data collection phase 1. Baseline (before intervention) 2. Endline (post-intervention)

09: Post-intervention data collection 1. Study participants from intervention group

2. Study participants from control group

**Module I: Socio-demographic characteristics of study participants**

**Instruction:** First, tell interviewee that you are going to ask her questions about herself. Second, properly explain to the study participant each question to elicit genuine response from the respondents. Then, request her to correctly respond to the questions. Please write the responses based on the respondents genuine responses (in field to be filled by data collectors).

| S.no | Questions | Response categories | Skip to | Remark |
| --- | --- | --- | --- | --- |
| 101 | What is your age (in completed years)? | __________ years |  |  |
| 102 | To which ethnic group do you belong? | \| 1. Sidama  2. Amhara  3. Oromo  4. Gurage  5. Wolayita  88.Other,specify_____ \| \| --- \| |  |  |
| 103 | What is your religious affiliation?  (encircle the response) | 1. Protestant Christian  2. Orthodox Christian  3. Catholic  4. Muslim  5.Other (specify) __________ |  |  |
| 104 | What is your highest educational level you have completed? | 1. Cannot read and write  2. Can read and write only  3. Primary education(1-8)  4. Secondary education(9-12)  5. College diploma  6. College/university degree and above |  |  |
| 105 | What is your occupation? | 1. Housewife  2. Farmer  3. Governmental employee  4. Merchant  5. Housemaid  6. Other (specify)……………… |  |  |
| 106 | What is your marital status? | 1. Not ever married…………….  2. Married  3. Divorced  4. Separated  5. Widowed  6. Cohabiting | Q110 |  |
| 107 | What is your husband‘s age in complete years? | _______________ |  |  |
| 108 | What is your husband‘s occupation? | 1. Governmental employee  2. Merchant  3. Farmer  4. Daily labourer  5. NGO employee  6. Private organization employee  7. Other (specify)……………… |  |  |
| 109 | What is your husband‘s educational status? | 1. Cannot read and write  2. Can read and write only  3. Primary education(1-8)  4. Secondary education(9-12)  5. College diploma  6. College/university degree and above |  |  |
| 110 | What is your family size? (In number) | ________ |  |  |
| 111 | Which social media do you use? | 1. Listens the radio 2. Watches the television 3. Reads the newspaper 4. All 5. Others |  |  |

**Module II: Household wealth index of study participants**

| S.no | Questions | Response categories | Skip to | Remark |
| --- | --- | --- | --- | --- |
| 201 | What is the main source of drinking water for members of your household? | 1. Piped water 2. Tube well or Borehole 3. Protected well 4. Unprotected well 5. Protected spring 6. Unprotected spring 7. Rain water 8. Surface water (River/dam) 9. Lake/pond/stream/canal 10. Bottled water |  |  |
| 202 | What is the main source of water used by your household for other purposes such as cooking and handwashing? | 1. Piped water 2. Tube well or Borehole 3. Protected well 4. Unprotected well 5. Protected spring 6. Unprotected spring 7. Rain water 8. Surface water (River/dam) 9. Lake/pond/stream/canal 10. Bottled water |  |  |
| 203 | Where is that water source located? | 1. In own dwelling 2. In own yard/plot 3. Elsewhere |  |  |
| 204 | How long does it take to go there, get water, and come back? | Minutes . . . . . . . . . . . . . . . .  998. Don’t know |  |  |
| 205 | In the past two weeks, was the water from this source not available for at least one full day? | 1. Yes 2. No   998. Don’t know |  |  |
| 206 | Do you do anything to the water to make it safer to drink? | 1. Yes 2. No   998. Don’t know |  |  |
| 207 | What kind of toilet facility do members of your household usually use?  IF NOT POSSIBLE TO DETERMINE, ASK PERMISSION TO OBSERVE THE FACILITY. | 1. Flush or pour flush toilet 2. Ventileted improved pit latrine 3. Pit latrine with slab 4. Pit latrine without slab/open pit 5. Compositing toilet 6. Bucket toilet 7. Hanging toilet 8. No facility/bush/field |  |  |
| 208 | Do you share this toilet facility with other households? | 1. Yes 2. No |  |  |
| 209 | Including your own household, how many households use this toilet facility? | 998. Don’t know |  |  |
| 210 | Where is this toilet facility located? | 1. In own dwelling  2. In own yard/plot  3. Elsewhere |  |  |
| 211 | What type of fuel does your household mainly use for cooking? | 1.Electricity  2. Liquefied petroleum gas  3. Natural gas  4.Biogas  5.Keresone  6. Charcoal  7. Wood  8. Straw/shrubs/grass  9. Agricultural crop  10. Animal dung  11.No food cooked in household |  |  |
| 212 | Is the cooking usually done in the house, in a separate building, or outdoors? | 1. In the house  2. In a separate building  3. Outdors  4. Others (specify)……………… |  |  |
| 213 | Do you have a separate room which is used as a kitchen? | 1. Yes  0. No |  |  |
| 214 | Who is the owner of the house? | 1. Me 2. Rental 3. Family 4. Relative 5. Others (specify)………….. |  |  |
| 215 | How many rooms in this household are used for sleeping? | -------------------------- |  |  |
| 216 | Main material of the roof of the house? | 1. Natural roofing (no roof, mud, and sod) 2. Rudimentary roofing (rustic mat/plastic shee, reed/bamboo, wood planks,and cardboard) 3. Finished roofing (metal/corrugated iron, wood, calamine/cement, ceramic tiles, roofing shingles) |  |  |
| 217 | Main material of the floor of the house? | 1. Natural floor (Earth/sand, dung) 2. Rudimentary floor (wood planks,and palm/bamboo) 3. Finished floor (parquet or polished wood, vinyl or asphalt strips/ plastic tiles, cement, ceramic tiles, carpet) |  |  |
| 218 | Main material of the wall of the house? | 1. Natural walls (no walls, cane/palm/trunks/bamboo/ree, dirt) 2. Rudimentary walls (bamboo with mud, stone with mud, uncovered adobe, plywood, cardboard, and reused wood ) 3. Finished floor (cement, stone with lime/cement, bricks, cement blocks, covered adobe, wood planks/shingles) |  |  |
| 219 | Does this household own any livestock, herds, other farm animals, or poultry? | 1.Yes  0.No | 222 |  |
| 220 | How many of the following animals does this household own?  IF NONE, RECORD ‘00’.  IF 95 OR MORE, RECORD ‘95’.  IF UNKNOWN, RECORD ‘98’.  a) Milk cows, oxen or bulls?  b) Other cattle?  c) Horses/donkeys/mules?  d) Camels?  e) Goats?  f) Sheep?  g) Chickens/poultry?  h) Beehives? | a) Milk cows, oxen or bulls………  b) Other cattle………………….  c) Horses/donkeys/mules……….  d) Camels……………………..  e) Goats………………………  f) Sheep………………………….  g) Chickens/poultry……………..  h) Beehives……………………. |  |  |
| 221 | Do you have separate rooms for cattle? | 1.Yes  0.No |  |  |
| 222 | Does any member of this household own any agricultural land? | 1.Yes  0. No | 224 |  |
| 223 | How many hectares of agricultural land do members of this household own? | ---------------------------hectares |  |  |
| 224 | Does your household have:   1. Electricity? 2. A radio? 3. A television? 4. A non-mobile phone? 5. A computer? 6. A refrigerator? 7. A table? 8. A chair? 9. A bed with cotton/sponge/spring mattress? 10. An electric mitad? 11. A kereson lamp/pressure lamp? | Yes No   1. Electricity……..1 0 2. Radio…………..1 0 3. Television………1 0 4. Non-mobile phone.1 0 5. Computer…………1 0 6. Refrigerator………1 0 7. Table………………1 0 8. Chair………………1 0 9. Bed with cotton/sponge/spring mattress…………..1 0 10. Electric mitad…….1 0 11. Kereson lamp/pressure lamp……………….1 0 |  |  |
| 225 | Does any member of this household own:  a) A watch?  b) A mobile phone?  c) A bicycle?  d) A motocycles/scooter?  e) An animal drawn cart?  f) A car/truck?  g) A boat with motor?  h) A bajaja? | Yes No a) Watch . . . . . . . . . . 1 0  b) Mobile phone . . . . . 1 0  c) Bicycle. . . . . . . . . . . 1 0  d) Motocycles/scooter. . 1 0  e) Animal drawn cart . . .1 0  f) Car/truck . . . . . . . . . . .1 0  g) Boat with motor . . . . . 1 0  h) Bajaja . . . . . . . ………1 0 |  |  |
| 226 | Does any member of this household have a bank account? | 1. Yes 2. No |  |  |
| 227 | Does any member of this household have a microfinanceaccount? | 1. Yes 2. No |  |  |

**Module III: Reproductive history of study participants**

| S.no | Questions | Response categories | Skip to | Remark |
| --- | --- | --- | --- | --- |
| 301 | What was your age when you were first married? | 998. I don’t know |  |  |
| 302 | What was your age at your first pregnancy? | 998. I don’t know |  |  |
| 303 | Have you ever become a pregnant? | 1. No 2. Yes | 3 |  |
| 304 | If yes for question 303, how many times? | --------------------------- |  |  |
| 305 | Have you ever faced abortion? | 1. No 2. Yes | 307 |  |
| 306 | If yes for question 305, how many times? | ---------------------------- |  |  |
| 307 | Have you ever delivered a child? | 1. No 2. Yes | 311 |  |
| 308 | If yes for question 306, how many times? | ----------------------------- |  |  |
| 309 | How many of them were live births? | ------------------------------ |  |  |
| 310 | How many of them were delivered at home? | --------------------------- |  |  |
| 311 | Infection during the current pregnancy (e.g., UTI, periodontal disease) | 1. No 2. Yes |  |  |
| 312 | Do you have any family history of hypertension? | 1. No 2. Yes | 3 |  |
| 313 | If Yes to Q312, who? | 1. Father 2. Mother 3. Sister 4. Grand mother 5. Other (specify)……………….. |  |  |
| 314 | Have you ever experienced death of fetus or stillbirth (fetal death at or after 28 weeks of pregnancy)? | 0. Yes  1. No  998. I don‘t remember |  |  |
| 315 | Have you ever experienced death of neonate (age less than seven days)? | 0. Yes  1. No  998. I don‘t remember | end |  |
| 316 | If yes to Q 314, how many times? | _____________ |  |  |
| 317 | If yes to Q 314, where was the neonate born? | 1. Home  2. Health institution  3. Other (specify) _________ |  |  |

**Module IV: Maternal health service utilization**

| S.no | Questions | Response categories | Skip to | Remark |
| --- | --- | --- | --- | --- |
| 401 | Was your last pregnancy planned? | 1. Yes  0. No | 405 |  |
| 402 | If ‘No’ to Q401, what was reason? | 1. Lack of modern contraceptives  2. Failure of contraceptive method  3. Others (specify)………………… |  |  |
| 403 | If ‘No’ to Q401, don’t want at all the last pregnancy? | 1. Yes  0. No |  |  |
| 404 | If ‘No’ to Q401, do you want after months of delivery? | 1. Yes  0. No |  |  |
| 405 | Did you face any health problem during your the last pregnancy? | 1. Yes  0. No | 407 |  |
| 406 | If ‘Yes’ to Q 405, what were they?  (Do not read the choices) | 1. Excessive vaginal bleeding 2. A high grade fever 3. Bad smelling vaginal discharge 4. Convulsions 5. Severe headache 6. Face/ hand swelling 7. Persistent vomiting 8. High blood pressure 9. Other (specify)_________ |  |  |
| 407 | Did you visit a health facility during your last pregnancy? | 1. Yes  0. No | 409 |  |
| 408 | If yes to Q 407, reason for visit? | 1. Pregnancy related health problem  2. Health problems not related to pregnancy  3. For antenatal care  4. Other (specify) ------------------ |  |  |
| 409 | Did you obtain ANC visit during your last pregnancy? | 1. Yes  0. No  998. Don’t remember | 428  428 |  |
| 410 | If yes to Q409, how many times did you obtain ANC? | 998.Don’t know/remember | 412 |  |
| 411 | Mention chronologically in how many months did you obtain ANC visits? | 1^st^ ANC ……………….months  2^nd^ ANC ………………months  3^rd^ ANC………………..months  4^th^ ANC……………….months  999. Don’t know/remember |  |  |
| 412 | From where did you receive ANC service? | 1. Government hospital  2. Private hospital  3. Faith based charity ospital  4. Health centre  5. Private clinic  6. Faith based charity clinic  7. Health post  8. Other (specify) ________________ |  |  |
| 413 | Why did you prefer the above stated health facility (Q412)? | 1. Close to my house  2. Competent health worker  3. Other (specify) _________ |  |  |
| 414 | Who provided you ANC in the last pregnancy? | 1. Doctors 2. Nurse 3. Midwifes 4. Health officers 5. Health extension workers 6. Traditional birth attendant 7. Other (specify)___________ |  |  |
| 415 | Did you receive any tetanus toxoid (TT) injection during your last ANC visits? | 1. Yes 2. No   998. Don’t remember |  |  |
| 416 | If ‘Yes’ to Q415, how many times? | __________________ |  |  |
| 417 | Did you receive any laboratory test during your last ANC visits? | 1. Yes  0. No  998. Don’t remember |  |  |
| 418 | If ‘Yes’ to Q417, what tests? | 1= Blood (Blood groups)  2= Blood (Syphilis test)  3= Stool  4= Urine  5= Other (Specify)……………………… |  |  |
| 419 | Did you receive any health message during your last ANC visits? | 1. Yes  0. No  998. Don’t remember | 428  428 |  |
| 420 | If ‘Yes’ to Q419, what messages? | 1= Breastfeeding  2= Danger Signs Maternal  3= Birth Planning  4= Facility Delivery  5= Danger Signs New-born  6= Basic Care New Born  8= Other (Specify)……………………… | 421 |  |
| 421 | Which danger signs of were you informed about?  (Don‘t read the choices) | l= Severe headache  2= Blurry Vision  3= Reduced or absent foetal movement  4= High blood pressure  5= Oedema of the face  6= Oedema of the hands & feet  7= Convulsions  8= Vaginal bleeding  9= Lower abdominal pain  l0=None of above |  |  |
| 422 | Were you informed where to go during danger signs problems stated above (Q421)? | 1. Yes  0. No  998. Do not remember |  |  |
| 423 | Where you informed about where to deliver during your last baby? | 1. Yes  0. No  998. Do not remember | 425  425 |  |
| 424 | If yes to Q 423, where were you recommended to deliver? | 1. Home 2. Health post 3. Health centre 4. Private clinic 5. Faith based charity clinic 6. Government hospital 7. Private hospital 8. Faith based charity hospital 9. Others specify……………… |  |  |
| 425 | Were you informed about who should attend you during delivery? | 1. Yes  0. No  998. Do not remember | 427  427 |  |
| 426 | If ‘Yes’ to Q 425, who was recommended to attend your delivery? | 1. Doctors 2. Nurse/Midwifes/health officers 3. HEWs 4. Traditional birth attendant 5. Other (specify)__________ |  |  |
| 427 | Were you informed to prepare the following items before your delivery date? | 1. Transport  2. Save money  3. Identify blood donor  4. Nothing |  |  |
| 428 | If you didn’t ANC visits, can you tell me the reasons?  (Multiple answer is possible)  (Do not read the choice) | 1.No or little Knowledge about ANC  2. No health problem encountered  3. Health institution is too far from my home  4. Long waiting time  5. Poor handling by health care providers  6. Lack of transportation  7. Lack of time to go to health institution  8. Other (specify)---------------------- |  |  |
| 429 | Did you plan to deliver in health facility? | 1. Yes  0. No  998. Don’t remember | 431 |  |
| 430 | If no to Q429, can you tell me the reasons?  (Multiple answer is possible)  (Do not read the choice) | 1.No or little Knowledge about institutional delivery  2. No health problem encountered  3. Health institution is too far from my home  4. Poor handling by health care providers  5. Lack of transportation  6. Other (specify)---------------------- |  |  |
| 431 | Before your date of delivery for your last pregnancy did you or your family prepared the following? | 1. Transport  2. Save money  3. Identify appropriate health facility  4. Identify blood donor  5. Identify skilled attendant  6. Nothing |  |  |
| 432 | Where did you give birth to your last child? | 1. Home 2. Health post 3. Health centre 4. Private clinic 5. Faith based charity clinic 6. Government hospital 7. Private hospital 8. Faith based charity hospital 9. Others specify……………… | 442 |  |
| 433 | If your last delivery is in health institution, when did you go to the health institution? | 1. At the beginning of labour  2. 6-12 hours after the beginning of labour  3. 13-18 hours after the beginning of labour  4. 19-24 hours after the beginning of labour  5. Other specify __________ |  |  |
| 434 | If your last delivery is in health institution, what was the mode of delivery? | 1. Spontaneous vaginal delivery  2. Instrumental delivery  3. Caesarean section  4. Other (specify) __________ |  |  |
| 435 | Who conducted or assisted you in the last delivery? | 1. Doctors 2. Nurse 3. Midwife 4. Health officer 5. Health extension worker 6. Traditional birth attendant (TBA) 7. Other (specify)___________ |  |  |
| 436 | Did anyone other than health care providers assist you with the delivery of last baby? | 1. Yes 2. No |  |  |
| 437 | If ‘Yes’ to Q436, who assisted you with the delivery?  Anything else?  RECORD ALL RESPONSES | 1. Mother 2. Mother-in law 3. Sister 4. Sister-in law 5. Other member of in-laws home 6. Other member of fathers 7. Other relatives 8. Neighbour 9. Other specify…………………… |  |  |
| 438 | Were any of the following procedures performed at the time of delivery? | 1. Blood transfusion  2. Fluid  3. Injection  4. Episiotomy  5. Received tablet Misoprostol |  |  |
| 439 | If injection provided. What was the injection for?  (Do not read the choice)  RECORD ALL RESPONSES | 1= Speed up labour  2 = Reduce bleeding  3= Stop convulsions  4=Other (Specify)  999=Don’t Know |  |  |
| 440 | For how long were you in labour during the last delivery? | 1. Less than 12 hours  2. 12- 24 hours  3. 25- 36 hours  4. 37 – 48 hours  5. More than 48 hours |  |  |
| 441 | What was the condition of the baby at birth in the last delivery? | 1. Born alive  2. Still birth (born died)  3. Born alive but died immediately |  |  |
| 442 | If responded at home to Q432, why did you prefer to deliver at home?  (Don‘t read the choice) | 1. The labour was short  2. No nearby health facility  3. The service is not available in the nearby health facility  4. Lack of money for transport  5. Poor handling by health professionals  6. Prefer to deliver in the presence of relatives  7. Fear of manipulation (like episiotomy)  8. Lack of privacy in the health institutions  9. I didn‘t know the importance of health facility delivery  10.Opinions of (husband, neighbours, other community members)  11. Other (specify) ____________ |  |  |
| 443 | Have you encountered any health problems during labour in the last delivery? | 1. Yes  0. No  999. I don‘t remember | 456 |  |
| 444 | If yes to Q 443, what were they?  (Do not read the choices) | 1 = Excessive Vaginal bleeding  2 = Foul smelling discharge  3 = High grade fever  4 = Baby’s hand or feet come first  5= Baby bad position/ mal presentation  6= Prolong labour (> l2 Hours)  7= Retained placenta/placental expelled late  8 = Torn uterus  9= Prolapsed cord  10 = Cord around the neck  11= Convulsions  l2 = Perineal tear  l3= None of above | 456 |  |
| 445 | Did you seek any care or treatment for any of these/this complication(s)?  Probe: Any care/treatment receive at home | 1. Yes 2. No | 456 |  |
| 446 | If ‘Yes’ to Q445, where did you seek care/treatment for these/this complication(s)?  RECORD ONLY ONE ANSWER- WHERE WOMAN OR FAMILY FIRST SOUGIIT CARE/TREATMENT | 1=Home  2=Health post  3=Health centre  4= Private clinic  5=Faith based charity clinic  6=Government hospital  7=Private hospital  8=Faith based charity hospital  9=Others specify……………… |  |  |
| 447 | If ‘Yes’ to Q445, who provided this care/treatment?  RECORD ONLY ONE ANSWER | 1=Doctors  2=Nurse  3=Midwife  4=Health offer  5=Health extension worker  6=Traditional birth attendant (TBA)  7=Family  8=Relative  9=Neighbour  10=Other (specify)___________ |  |  |
| 448 | Were you referred to any other place for care/treatment of complication(s)?  PROBE. CARE INSIDE/OUTSIDE THE HOME? | 1. Yes 2. No | 451 |  |
| 449 | If ‘Yes’ to Q448, where were you referred? | 1. Health post 2. Health centre 3. Hospital 4. Prayer 5. Other specify…………………….. |  |  |
| 450 | Did they tell you the reasons for referral? | 1. Yes 2. No | 455 |  |
| 451 | If ‘Yes’ to Q449, why were you told to seek treatment at this than other place?  RECORD ALL RESPONSES | 1 = No surgery equipment  2 = High blood pressure  3 = For better treatment  4 = Doctor unavailable  5 = No arrangement for blood transfusion  6 = Baby’s upward position  7 = Some part of baby came out  8 =Baby Stool in womb  9 = cervix did not open  10 = Other specify  999 = Don’t know |  |  |
| 452 | Did you go to this place? | 1. Yes 2. No   999. Don’t remember | 455 |  |
| 453 | If ‘Yes’ to Q452, how long did you wait to seek care for this complication? | _______________ |  |  |
| 454 | If ‘Yes’ to Q449, did anybody call you after you want to the referred place, to see how you were doing? | 1. Yes 2. No   999. Don’t remember |  |  |
| 455 | Why did you not go to the referred place? | 1 = Woman didn’t think necessary  2 = Husband /family didn’t think necessary  3 = Facility too far  4 = No transport  5 = No child care  6 = too expensive  7 = Services are poor quality  8 = Didn’t know where to go  9 = No time to go  10 = Money not easily available  I l = Other (Specify)  99 = Don’t know |  |  |
| 456 | Did you use the transport you identified during pregnancy? | 1. Yes 2. No |  |  |
| 457 | Did you use the money that you saved during pregnancy? | 1. Yes 2. No |  |  |
| 458 | Have you encountered any health problems during the first 6 weeks of post-partum? | 1. Yes  0. No  999. I don‘t remember | 460 |  |
| 459 | If ‘Yes’ to Q 455, what were they?  (Do not read the choices) | 1 = Excessive vaginal bleeding  2 = Foul smelling discharge  3 = High fever  4 = Inverted nipples  5 = Tetanus  6 = Retained placenta  7 = Severe abdominal pain  8 = Convulsions  9 = Engorged breasts  10 = None of above |  |  |
| 460 | Did you get medical check-up or PNC after your last delivery within 42 days? | 1. Yes  0. No  999. I don‘t remember |  |  |
| 461 | If ‘Yes’ to Q461, when did you get it for the first time after delivery? | 1. After ______ hours  2. After ______ days  3. After ______ weeks |  |  |
| 462 | If ‘Yes’ to Q461, how many times did you obtain PNC within 42 days? | 998.Don’t know/remember |  |  |
| 463 | Where did you get the check-up for the first time? | 1= Home  2=Health post  3=Health centre  4=Hospital  5=Others specify……………… |  |  |
| 464 | What additional services did you get? | 1. Child vaccination  2. Family planning methods  3. Counselling about breast feeding  4. Others specify ____________ |  |  |
| 465 | From where did you receive PNC service? | 1. Government hospital  2. Private hospital  3. Faith based charity ospital  4. Health centre  5. Private clinic  6. Faith based charity clinic  7. Health post  8. Other (specify) ________________ |  |  |
| 466 | Who provided you PNC in the last childbirth? | 1=Doctors  2=Nurse  3=Midwifes  4=Health officers  5=Health extension workers  6=Traditional birth attendant  7=Other (specify)___________ |  |  |
| 467 | If you didn’t PNC visits, can you tell me the reasons?  (Multiple answer is possible)  (Do not read the choice) | 1.No or little Knowledge about ANC  2. No health problem encountered  3. Health institution is too far from my home  4. Long waiting time  5. Poor handling by health care providers  6. Lack of transportation  7. Lack of time to go to health institution  8. Other (specify)---------------------- |  |  |
| 468 | What was your husband‘s attitude towards MHSU? | 1. Positive  2. Negative  3. Don‘t know |  |  |
| 469 | Who is the decision maker in your HH to seek care from modern health institution? | 1. Both myself and my husband  2. My husband  3. My self  4. Other (specify) _____________ |  |  |
| 470 | Is road accessible to near health facility? | 1. Yes 2. No |  |  |
| 471 | Have you taken model household training? | 1. Yes 2. No |  |  |

**Module V: Knowledge and attitudes of study participants towards MHSU**

**1. Knowledge of study participants towards MHSU**

| S.no | Questions | Response categories | Skip to | Remark |
| --- | --- | --- | --- | --- |
|  | **Knowledge questions for ANC** |  |  |  |
| 501 | Do you know about the ANC service? | 1. 0. No   Yes 1. Yes | 503 |  |
| 502 | If yes to Q 501, is ANC important for mothers and fetus positive health outcome? | 1. No 2. Yes |  |  |
| 503 | When does the first ANC visit is recommended? | 1. Greater than 16 weeks 2. Less than 16 weeks |  |  |
| 504 | Do you know total number of ANC visits recommended for the pregrant women throughout her preganancy period? | 1. Less than 4 2. Four and more |  |  |
| 505 | Where is the appropriate place for the first ANC visit? | 1. Health post 2. Helth center or hospital |  |  |
| 506 | Does a pregnant woman need to undergo the laboratory test during her ANC visit? | 1. No 2. Yes |  |  |
| 507 | Does a pregnant woman need to be provided the essential drugs during her ANC visit? | 1. No 2. Yes |  |  |
| 508 | Does a pregnant woman need to be provided the counseling during her ANC visit? | 1. No 2. Yes |  |  |
|  | **Knowledge questions for institutional delivery** |  |  |  |
| 509 | Do you know about the institutional delivery service? | 1. No 2. Yes |  |  |
| 510 | If yes to Q 501, is institutional delivery care important for mothers and newborn positive outcome? | 1. No 2. Yes |  |  |
| 511 | Which woman is recommended to give birth at HFs? | 1. High risk 2. All |  |  |
| 512 | Which place is safe for child delivery? | 1. Home 2. HFs |  |  |
| 513 | Which provider is skilled for child delivery? | 1. TBA, relative, friend 2. Health professional |  |  |
| 514 | Does a woman die due to the complications happen during child birth? | 1. No 2. Yes |  |  |
| 515 | Does a SBA at HFs can prevent maternal deaths that happen during the child birth? | 1. No 2. Yes |  |  |
|  | **Knowledge questions for PNC** |  |  |  |
| 516 | Do you know about the PNC service? | 1. No 2. Yes |  |  |
| 517 | If yes to Q 501, is PNC important for mothers and neonetus positive health outcome? | 1. No 2. Yes |  |  |
| 518 | Do you know total number of PNC visits recommended for the women throughout her pospartum period? | 1. Less than 3 2. Three and more |  |  |
| 519 | Where is the appropriate place for the first PNC visit? | 1. Health post 2. Helth center or hospital |  |  |
| 520 | Does a pregnant woman need to be provided the counseling during her PNC visit? | 1. No 2. Yes |  |  |
| 521 | Does a woman die due to the complications happen during postpartum period? | 1. No 2. Yes |  |  |
| 522 | Does a PNC at HFs can prevent maternal deaths that happen during the postpartum period? | 1. No 2. Yes |  |  |

1. **Attitude of study participants towards MHSU**

**Tool to assess client attitude towards MHSU**

1-Strongly disagree; 2- Disagree; 3- Neutral; 4- Agree; 5- Strongly agree

| S.no | Questions | 1 | 2 | 3 | 4 | 5 |
| --- | --- | --- | --- | --- | --- | --- |
|  | **Attitude questions for ANC** |  |  |  |  |  |
| 523 | Your attitude about the ANC is important for mothers' positive health outcomes? |  |  |  |  |  |
| 524 | Your attitude about the first ANC visit should be obtained in less than 16 weeks? |  |  |  |  |  |
| 525 | Your attitude about the four ANC visits is adequate for uncomplicated pregnancy? |  |  |  |  |  |
| 526 | Your attitude about the health post is an inappropriate place for the first ANC visit? |  |  |  |  |  |
| 527 | Your attitude about the pregnant woman needs to use the laboratory test during her ANC visit? |  |  |  |  |  |
| 528 | Your attitude about the pregnant woman needs to be providing the essential drugs during her ANC visits? |  |  |  |  |  |
| 529 | Your attitude about the pregnant woman needs to be providing counsel during her ANC visits? |  |  |  |  |  |
| 530 | Your attitude about the women's reasons not to go to a health facility for ANC is mainly because of high opportunity cost? |  |  |  |  |  |
| 531 | Your attitude about the women's reasons not to go to a health facility for ANC because HCPs don't treat them respectfully? |  |  |  |  |  |
|  | **Attitude questions for institutional delivery** |  |  |  |  |  |
| 532 | Your attitude about institutional delivery care is important for mothers’ positive health outcomes? |  |  |  |  |  |
| 533 | Your attitude about all women is recommended to give birth at HFs? |  |  |  |  |  |
| 534 | Your attitude about the HFs is safe for child delivery? |  |  |  |  |  |
| 535 | Your attitude about the HCPs being skilled professionals for child delivery? |  |  |  |  |  |
| 536 | Your attitude about the woman dying due to the complications happens during childbirth? |  |  |  |  |  |
| 537 | Your attitude about the SBA at HFs can prevent maternal deaths that happen during childbirth? |  |  |  |  |  |
| 538 | Your attitude about the importance of having a plan on possible pregnancy complications? |  |  |  |  |  |
| 539 | Your attitude about the delivery being attended by male HCPs isn’t shameful? |  |  |  |  |  |
| 540 | Your attitude about the delivery on the delivery bed isn't shameful in the labour ward? |  |  |  |  |  |
| 541 | Your attitude about the women doesn’t go to a health facility for delivery is due to high opportunity cost? |  |  |  |  |  |
| 542 | Your attitude about the women doesn’t go to a health facility for delivery due to HCPs doesn’t treat them respectfully? |  |  |  |  |  |
|  | **Attitude questions for PNC** |  |  |  |  |  |
| 543 | Your attitude about the PNC is important for mothers’ positive health outcomes? |  |  |  |  |  |
| 544 | Your attitude about the 3 PNC visits is adequate for the women with uncomplicated delivery? |  |  |  |  |  |
| 545 | Your attitude about the health post is an inappropriate place for the first PNC? |  |  |  |  |  |
| 546 | Your attitude about the delivered woman needs to be provided the counsel during her PNC visit? |  |  |  |  |  |
| 547 | Your attitude about the woman dying due to the complications happens during the postpartum period? |  |  |  |  |  |
| 548 | Your attitude about the PNC visits in HFs can prevent maternal deaths that happen during the postpartum period? |  |  |  |  |  |
| 549 | Your attitude about the women doesn’t go to a health facility for PNC is due to high opportunity cost? |  |  |  |  |  |
| 550 | Your attitude about the women doesn’t go to a health facility for PNC is due to HCPs don’t treat them respectfully? |  |  |  |  |  |

**Module VI: Socio-cultural predictors**

| S.no | Questions | Response categories | Skip to | Remark |
| --- | --- | --- | --- | --- |
| 601 | Can you tell me any socio-cultural beliefs that affect MHSU in your community?  (Do not read the choices)  RECORD ALL RESPONSES | 1. Availability and influence of traditional healers  2. Availability and influence of spiritual healers   1. Availability and influence of traditional birth attendants 2. Holy water 3. Fear of using MHS 4. Peer influence 5. Knowledge of community towards MHSU 6. Husband influence 7. Father and mother in law influence 8. Other (specify)…………. |  |  |

**Model VII: Perceived quality of MHSU**

**Tool to assess client perceived quality of antenatal care**

1-Very poor; 2- Poor; 3- Neutral; 4- Good; 5- Very good

|  | **ITEMS** | 1 | 2 | 3 | 4 | 5 |
| --- | --- | --- | --- | --- | --- | --- |
| 1 | Your perception about the friendliness shown towards you by the HCPs in the ANC |  |  |  |  |  |
| 2 | Your perception about the patience shown towards you when you did not cooperate with HCPs |  |  |  |  |  |
| 3 | Your perception about the promptness of the attention given by the HCPs when you needed it |  |  |  |  |  |
| 4 | Your perception about the enough time with HCPs during ANC visit |  |  |  |  |  |
| 5 | Your perception about the way your privacy was maintained by the HCPs in the ANC room |  |  |  |  |  |
| 6 | Your perception about the willingness of the HCPs to discuss about your concerns |  |  |  |  |  |
| 7 | Your perception about the your involvement in decision making |  |  |  |  |  |
| 8 | Your perception about the fairness of waiting time in the ANC visit |  |  |  |  |  |
| 9 | Your perception about the overall cleanliness of the HF |  |  |  |  |  |
| 10 | Your perception about the help you received from the health care workers to take care of fetus and yourself (ex: maintaining your cleanliness) |  |  |  |  |  |
| 11 | Your perception about the adequacy of information given to you on ANC benefits |  |  |  |  |  |
| 12 | Your perception about the adequacy of information given on expected date of delivery and gestational age |  |  |  |  |  |
| 13 | Your perception about the adequacy of information given to you to identify danger signals |  |  |  |  |  |
| 14 | Your perception about the skills of the HCPs to identify and manage health issues |  |  |  |  |  |
| 15 | Your perception about adequacy of information received to clarify any issues you had |  |  |  |  |  |
| 16 | Your perception about the Cleanliness of the ANC room |  |  |  |  |  |
| 17 | Your perception about adequacy of information received on the nutrition |  |  |  |  |  |
| 18 | Your perception about the information on Tetanus toixed vacination |  |  |  |  |  |
| 19 | Your perception about the information on blood test |  |  |  |  |  |
| 20 | Your perception about the information on urine test |  |  |  |  |  |
| 21 | Your perception about the information on weight, height measurement |  |  |  |  |  |
| 22 | Your perception about the information and blood pressure measurement |  |  |  |  |  |
| 23 | Your perception about an appointment clarity |  |  |  |  |  |

**Tool to assess mother perceived quality of institutional delivery care**

1-Strongly disagree; 2- Disagree; 3- Neutral; 4- Agree; 5- Strongly agree

| S.no | Questions | 1 | 2 | 3 | 4 | 5 |
| --- | --- | --- | --- | --- | --- | --- |
| 1 | I think there is good waiting area in the nearby health facility |  |  |  |  |  |
| 2 | I think there is good maternity ward’s toilets in the nearby health facility |  |  |  |  |  |
| 3 | Hand washing and shower is available in the nearby health facility |  |  |  |  |  |
| 4 | Infrastructures like electricity, and water is available in the nearby health facility |  |  |  |  |  |
| 5 | It is easy to get maternity ward starting from the gate |  |  |  |  |  |
| 6 | The health care providers in the nearby clinic are good at labor and delivery care |  |  |  |  |  |
| 7 | The health care providers in the nearby clinic are good at newborn care |  |  |  |  |  |
| 8 | The health care providers in the nearby clinic are good at pain management |  |  |  |  |  |
|  | | | | | | |
| 9 | How long does it take to reach to the nearby health facility on foot? | ________hrs | | | |  |
| 10 | Prompt transport service is available from home to the nearby health facility | 1. Yes  0. No | | | |  |
| 11 | Delivery service charge in the nearby health facility | 1. High  2. Moderate  3. Low | | | |  |

**Tool to assess client perceived quality of institutional postnatal care**

1-Very poor; 2- Poor; 3- Neutral; 4- Good; 5- Very good

|  | **ITEMS** | 1 | 2 | 3 | 4 | 5 |
| --- | --- | --- | --- | --- | --- | --- |
| 1 | Your perception about the friendliness shown towards you by the HCPs in the postnatal ward |  |  |  |  |  |
| 2 | Your perception about the patience shown towards you when you did not cooperate with HCPs |  |  |  |  |  |
| 3 | Your perception about the promptness of the attention given by the HCPs when you needed it |  |  |  |  |  |
| 4 | Your perception about the availability of pain relief during the postpartum period |  |  |  |  |  |
| 5 | Your perception about the way your privacy was respected by the HCPs in the postnatal ward |  |  |  |  |  |
| 6 | Your perception about the willingness of the HCPs to discuss about your concerns |  |  |  |  |  |
| 7 | Your perception about the way HCPs treated your family members |  |  |  |  |  |
| 8 | Your perception about the help given for the initiation of breast feeding in the labour room |  |  |  |  |  |
| 9 | Your perception about the help you received from the HCPs to take care of your baby |  |  |  |  |  |
| 10 | Your perception about the help you received from the health care workers to take care of yourself (ex: maintaining your cleanliness) |  |  |  |  |  |
| 11 | Your perception about the adequacy of information given to you on taking care of the baby |  |  |  |  |  |
| 12 | Your perception about the adequacy of information given on proper method of breast feeding |  |  |  |  |  |
| 13 | Your perception about the adequacy of information to identify danger signals following delivery, for the mother & the baby |  |  |  |  |  |
| 14 | Your perception about the skills of the HCPs to identify and manage health issues of your baby |  |  |  |  |  |
| 15 | Your perception about the skills of the HCPs to identify and manage health issues in relation to you |  |  |  |  |  |
| 16 | Your perception about adequacy of information received to clarify any issues you had |  |  |  |  |  |
| 17 | Your perception about the Cleanliness of the ward |  |  |  |  |  |
| 18 | Your perception about the Cleanliness of the toilets & washrooms |  |  |  |  |  |
| 19 | Your perception about adequacy of space in the postnatal ward |  |  |  |  |  |
| 20 | Your perception about the availability of adequate facilities in the ward in relation to the number of patients |  |  |  |  |  |
| 21 | Your perception about adequacy of delivery beds in the labour room |  |  |  |  |  |
| 22 | Your perception about the availability of adequate numbers of HCPs to assist you |  |  |  |  |  |
| 23 | Your perception about the ability to get some rest in the postnatal ward (without the interferences such as light, noise, ward activities) |  |  |  |  |  |

**Module IIX: Knowledge of ODS during pregnancy, childbirth and postpartum periods**

| S.no | Questions | Response categories | Skip to | Remark |
| --- | --- | --- | --- | --- |
| 801 | Please tell me what complications may occur during pregnancy that needs rnedical care?  (Do not read the choices)  Anything else?  RECORD ALL RESPONSES | l= Severe headache  2= Blurry vision  3= Reduced or absent foetal movement  4= High blood pressure  5= Oedema of the face  6= Oedema of the hands & feet  7= Convulsions  8= Vaginal bleeding  9= Lower abdominal pain  l0=None of above |  |  |
| 802 | Now, please tell me what are the complications in women during child birth that need medical treatment?  (Do not read the choices)  Anything else?  RECORD ALL RESPONSES | 1 = Excessive vaginal bleeding  2 = Foul smelling discharge  3 = High grade fever  4 = Baby's hand or feet come first  5= Baby bad position/ mal presentation  6= Prolong labour (> l2 Hours)  7= Retained placenta  8 =Torn uterus  9= Prolapsed cord  10 = Cord around the neck  11= Convulsions  l2 = Perineal Tear  l3= None of above |  |  |
| 803 | Now, please tell me what are the complications in women immediately child birth that need medical treatment?  (Do not read the choices)  Anything else?  RECORD ALL RESPONSES | 1 = Excessive vaginal bleeding  2 = Foul smelling discharge  3 = High fever  4 = Inverted nipples  5 = Tetanus  6 = Retained placenta  7 = Severe abdominal pain  8 = Convulsions  9 = Engorged breasts  10 = None of above |  |  |

**Module IX: Practices of BPCR**

| S.no | Questions | Response categories | Skip to | Remark |
| --- | --- | --- | --- | --- |
| 901 | During pregnancy and before the delivery, did you or your family make any plans for the birth? | l= Yes  0= No  999= Don't Remember | 906 |  |
| 902 | If yes to Q901, which plans did you or your family makes for the birth of (NAME)?  (Do not read the choices)  Anything else?  RECORD ALL RESPONSES | l= Identify proper closer HFs for childbirth  2= Identify SBA for deliver  3= Save money and material resources  4= Identify proper transport for delivery  5= Identify and fixing the compatible blood group givers |  |  |
| 903 | If identified facility for delivery. Where did you plan to give birth to (NAME)?  (Do not read the choices) | 1. Home 2. Health post 3. Health centre 4. Hospital 5. Others specify……………… |  |  |
| 904 | If identified provider for delivery. Who did you select as the birth attendant?  (Do not read the choices) | 1. Doctors 2. Nurse/Midwifes 3. Health extension workers 4. Traditional birth attendant 5. Other (specify)___________ |  |  |
| 905 | If identified transport. Did you use the transport you identified during pregnancy? | 1. Yes 2. No |  |  |
| 906 | If saved money. Did you use the money that you saved during pregnancy? | 1. Yes 2. No |  |  |

**Thank you for your participation!!!**

1. **QA’MISO**

**Qa’miso I: MASHALAQQETE QOOLA**

**Hawaasi Yuniversite**

**HIKKIMINNUNNA FAYYIMMATE SAYINSE KOLLEEJE**

**Dagoomu Fayyimmate Rosi Mine**

**Xiinxallote umo: DIRINSA ILATE DEERRIRA NOO AMUWI FAYYIMMARA UYINANNI OWAANTE WOYYEESSA Sidaamu Dagoomu Qoqqowu Mootimma, Itophiya**

AYIDDE KEEREHO!! Su’ma’ya _________________ yinannie, ani xiinxallote taje gamba aseemoho.

Fayyimmate rosi horo amuwu fayyimmate owaante horora meentu fayyimmate qodhaano horonsi’ratenni afi'rino rosi marichi horo abbinoronna amuwu fayyimma aana luphi yiino qeechi maati amuwaho fayyimmate owaante horo aana dagoomu giddo yaanno qaru koorinniiti.

**XIINXALLOTE GOOFIMARCHU MIXONKE**

Amuwu reyo dagankera ikko kalqete qarraati.Qajeelino amuwi fayyimma owaataasine shiimu baatooshshinni calla ikkado lifinxenni amuwu xissonna reyo ajishanno qarra tirate hayyooti. Xiinxallote akeeki qaru guulchu hedo amuwaho rosichonsa luphi assatenna fayyimansa agarate. Xiinxaallo xiinxaallinannihu 6 aganaati.Tenne kaimu taje gamba assinihu gedensaanni, dooramino amuwira ikkado fayyimmate rosicho amuwu fayyimma daafira uyinanni. Ikkado rosicho uynihu gedensaanni, awuwu rosicho afi'rihu gedensaanni, fayyimmansara marichi egennonna rosicho afi'ri yaatenna ikkado fayyimmate rosicho afi'rinokki amuwira may badooshshi no yaateetinna heewisiisateeti rosicho afi'rinohanna afi'rinokki amuwa.

Taje tini horontanni amuwu fayyimma daafira callaati.

**XIINXAALLOTE BEEQQORA DOORROONNI HAYYO**

Xiinxaallote beeqqora doorroonni hayyo sassaete. Konni daafira ati beeqqaancho ikkootta.Borri-xa'mote aattanke dawaro amatenna daaimu xissoranna reyo ittisate aatenke taje luphiima kaa’litanonke.Sumuu yaattae ikkiro, borri-xa’mo nabbawa hanafeemmo.Taje aattata mittu mannino fojo horontanni dikullanni.Sumuu yaa giwittaro borri-xa’mo agure sa’’a dandaatta.Xiinxallo mereeho agurate qoossokki agarantinote.Wolu xiinxallaanchi nafa fojokki afa dindadaanno.

**XIINXALLOTE BEEQQOOTTA DAAFIRA MITTE HULULO QARRI DINO**

Xiinxallote beeqqootta daafira mite mexamme woy huluullo heedhoonkehe!

Huluullamittaro dee’ni yaa dandaatta.

.

**Xiinxallote Horo**

Xiinxallo amuwaho uytanno horo umikkihunni Amuwu fayyimmate horo maatiro afate, layinkihunni Amuwaho fayyimmate owaantera marichi badooshshi nooro afatenna awuwu godowara albaanni may qarri xaadannonsaro balaxe huwanyo heedhaansa gede assatenna rosichonsa buuxate.Qoleno amuwu fayyimmate owaantera may badooshshi nooronna awuwu fayyimmate owaantera hiittene amaati owaante afi’ra hasiissannoti yee badateeti. Anfe ka’niro taaltino amaale aatenna wole fayyimmate uurrinshsha tareessateeti.Ate beeqqo xiinxaallote uytanno kaa’lo ate qachira no awuwira fayyimmansa afaateeti.Ledotenno ikkado fayyimmate rosicho qachikkira uyineemo gede kaa’lannonke. Ikkado fayyimmate rosicho dagankenna gobbanke.

**Xiinxallote beeqqo lainohunni baatooshshu dino.**

Xiinxallote beeqqora baatooshshu dino ikkinohura, beeqqo assootta daafira lowo galati noe..

**FOJJO LAINOHUNNI.**

Wo’manti taje kulattaeti, lowo geeshsha fojo wolu mannira dikuleemmo .Qole ate su’ma horontanni diborreeseemmo.Horontanni dawarootta dawaro ayeno diafanno.

**XIINXALLOTE BEEQQO ASSATTOHU SUMUU YIITTARONNA MEREEROHO XA’MO XA’ME GUDUMMOKKINO AGURATE QOOSSOKKI AGARANTINOTE!**

Xiinxallote beeqqo assa hoogakki mittoreno amuwu afi’ranno owaante hooltannohetano di"ikitino/mereeroho dawarte gudittakkinni agurte ha'ra dandaatta.

Xa’mo heedhuhero woy xawinnokkiri nooro, Amunuel Yooseefiha Hawaasi yuniversitera loosaasincho xa tenne yannara xiinxallote aana xa’ma dandiitinanni

Bilibilu kiirosi: 0915683578.

**Qa’miso II: MASHALAQQE AATE FAJJO**

SUMUU YAATTAE? DANCHA FAJJOOTEKKI IKKIRO: EE □ Dee’ni □

Tumi-qolokki ‘’ Ee’’ ikkiro ... Xa’mote Sai.

Xa’mamaanchu malaate________________________________

‘’Dee’ni’’ ikkiro Uurrissi!!

Xiinxallaanchu su’ma: Amanuel Yoseph

Bilbilu kiiro - +251-915683578 E-maile: [amanuelyoseph45@gmail.com](mailto:amanuelyoseph45@gmail.com)

Xa’manchu su’ma___________________ malaate__________

Xa’minoonni barra Tophiyu kiiro garinni borreessi. Xa’maanchu Qaali-xamo _____/_____/_____

Xa’mote dawaro: 1. Wo’manka dawaroonni. 2. Sumuu diyitino 3. Gama maahoyye yite gama gibbinno. 4. Xa’mamaancho hooggino mine.

Illaallisaanchu su’ma:______________ malaate_________ barra__/_____/____

**Qa’miso III: MASHALAQQETE QOOLA**

**Hawaasi Yuniversite**

**HIKKIMINNUNNA FAYYIMMATE SAYINSE KOLLEEJE**

**Dagoomu Fayyimmate Rosi Mine**

***Tini Borri-Xa’mo Qixxaabbinohu Itophiyu GiddoSidaamu Dagoomu Qoqqowu MootimmaAmuwu Ilate Dirinsa Keeraanchimma Buuxate Uynanninsa Owaante Aanaati***

**GAAMO I: Xaphoomu Taje**

01. Woradu su’ma: ____________

02. Olluu su’ma: ________________

03. Qachumu su’ma __________________

04. Minu kiiro____________________

05. Ayimmate Kaarde kiiro __________________

06. Xa’minoonni barra ____________Xa’mo hananfoonni yanna __________ Xa’mo gooffinno yanna.____________

07. Xa’mamaanchu towaanyo. Mashalaqqisaanchu qooxeessaho no:

1. Umikki towaanyo

2. Layinki towaanyo

3. Sayikki towaanyo.

08: Taje gamba assate gafa 1. Ikkito ikkate albaanni (before intervention) 2. Ikkito ikkite sa’eenna (post-intervention)

09: Ikkito ikkite sa’eenna gamba assinanni taje 1. Xiinxallote taji-mashalaqqisaano beeqqaano gaamo

2. Xiinxallote taji-mashalaqqisaano beeqqaanchiweelo gaamo

**Mojule I: Mashalaqqisaanonniha Dagi-mannoomittete Heeshsho Gade**

**Biddissa:** Umihunni, qaali-xamishsha assakkira balaxxe dawootto/a haja odeessi. Layinkimeeshsho, mashalaqqisaanokki halaalaancho xawishsha uytahe geeshsha tittirte dawoottore xawisi.Jeefoteno, mashalaqqisaanokki uytuhe taje garunni borreessite amadi ballo (Xiinxallaanchu taje gamba assanno ba’re).

| A.K | Xa’mo | Mashalaqqisaanote Tumo | Sai/ kubbi | Buuxo |
| --- | --- | --- | --- | --- |
| 101 | Dirikki me’’eho (xaphooma)? | __________ diro |  |  |
| 102 | Hiikko ayiddeeti? / Hiikko mineeti? | \| 1. Sidaama  2. Amaara  3. Oromo  4. Guraage  5. Wolayitta  6. woleno, xawisi____ \| \| --- \| |  |  |
| 103 | Ammanokki hiitteeti?  (dawarokki qoqqowi) | 1. Pirotestaante /Kiristaana  2. Ortodokise / Kiristaana  3. Kaatoolike  4. Musiliime  5.Wolete? (xawisi) __________ |  |  |
| 104 | Jawiidihu rosikki deerri hiikkonneeti? | 1.Nabbawanna borreessa didandeemmo/a  2. Nabbawanna borreessa calla dandeemmo/a  3. Umi dirimi rosaanchooti (1-8)  4. 2ki dirimi rosaanchooti (9-12)  5. Dippoloomu rosaanchooti  6. Digiretenna hakkuy aleeti |  |  |
| 105 | Loosu Qeechi maati? | 1. Minaama (mini ama)  2. Baatto loossi’re galinoha  3. Mootimmate Loosaasincho  4. Daddalaancho  5. Gobbate giddoydi  6.Wolereno (Xawisi) |  |  |
| 106 | Adhhammete Gara? | 1. Leexa …………….  2. Adhaminoha  3. Adhe/ ite Tirroha  4. Baxxinoha  5. Shiidhinoha  6. Woleno (Xawisi) _______ | X110 |  |
| 107 | Halaalaancho galtekki diro | _______________ |  |  |
| 108 | Galtekki loosi qeechi maati? | 1. mootimmate loosaasinchooti  2. daddalaanchoho  3. baatto loosi’re, saada ce’e galinoho  4. barru looso loose hee’rannoho  5. mootimma ikkitinokki (NGO) loosaasinchooti  6. hallanyu uurrinsha giddo loosanno  7. woleno, xawisi ------------------- |  |  |
| 109 | Galtekki rosi deerri mageeshshiho? | 1.Nabbawanna borreessa dandaannokkiho  2. Nabbawanna borreessa calla dandaannoho  3. Umi dirimi rosaanchooti (1-8)  4. Layinki dirimi rosaanchooti (9-12)  5. Dippolooma ronsoho  6. Digirenna hakkuy ale |  |  |
| 110 | Maatekki batinyi mageeshshiho? (kiirotenni) | ________ |  |  |
| 111 | Hiikkonne dagoomi-tuqi-xaado horoonsi’ratta? | 1. Macciishshinanni raadoone 2. La’nanni televishiine 3. Nabbambanni gaazeexa 4. Baalanka 5. woleno, xawisi |  |  |

**Mojule II: Maatete Miccinna Miinji Mayimma Mashalaqqe**

| A.K | Xa’mubba | Response categories | Sai/kubbi | Buuxo |
| --- | --- | --- | --- | --- |
| 201 | Maatekki aggannoha co’icha waa afidhannohu mamiinniiti? | 1. Baambu waa   1. Tuubbote waa 2. Huxxaminoha balete waa 3. Huxxaminokkiha balete waa 4. Huxxinoonniha buete waa 5. Huxxaminokkiha burqanno waa 6. Xeenu waa 7. Daadanno waa (lagga/xashshuwa) 8. Garba /kooficho/ga’rinowaa 9. Hayilaandete waa |  |  |
| 202 | Maatekki sagale qishi’ratenna anga hayishshi’rate horoonsidhanno waa hiikkiinni afidhanno? | 1. Baambu waa 2. Tuubbote waa 3. Huxxaminoha balete waa 4. Huxxaminokkiha balete waa 5. Huxxinoonniha buete waa 6. Huxxaminokkiha burqanno waa 7. Xeenu waa 8. Daadanno waa (lagga/xashshuwa) 9. Garba /kooficho/ga’rinowaa   10. Hayilaandete waa |  |  |
| 203 | Way afamanno dargi hiikkooti? | 1.Qae’yara   1. Hoowete giddoydinni 2. Wole darga |  |  |
| 204 | Waa dirri’ne hinganni yanna mageeshsha adhanno? | Daqiiqunni/xu’eessunni . . . . . . . . .  998. dibadoomma |  |  |
| 205 | Sa’uta lame lamala giddo way bae barra wo’ma keeshshino? | 1. Eewa 2. Dee’ni   998. dibuuxoomma |  |  |
| 206 | Way co’ichimma agarsiisate atewayinni agarrannire assoottori/tari no yite hedatta/o? | 1.Eewa   1. Dee’ni   998. dibuuxooma |  |  |
| 207 | Maatekki duucha yannara hiittooha shumate mine horoonsidhanno?  MITEEKKITE KULA FOKKITURO, DARGU GEESHSHA SA’E LAI | WAYINNI XAADINSOONNITA/ XAADINSOONNIKKITA   1. Baamba fanne horoonsi’nannita 2. Baambaho xaaddino way maashine 3. Ummooni balera waa horoonsi’ra 4. Base baalate waa horoonsi’ratenni 5. Shumate mini waa hiissine horoonsi’nanniro diafoommo/a 6. Baaldete waa 7. Gottiima shumate mine 8. Shumate mini dino/xawoho/ishinu giddo |  |  |
| 208 | Konne shumate mine wole maateno horoonsidhanno? | 1. Eewa 2. Dee’ni |  |  |
| 209 | Maatekki ledo woloota maate me’’e horoonsidhanno? | 1. 10 nni ajjannori 2. 10 woy hakkuy ali   998. dibuuxoomma |  |  |
| 210 | Shumate mine loonsoonnihu hiikkooti? | 1. mini giddooti  2. hoowenke giddooti  3. wolewaati |  |  |
| 211 | Maatekkira sagale loosi’rate hiittenne giirate wolqa horoonsidhinanni? | 1.Korreente  2. Liquide gase  3. Kalaqamu gaaze  4.Obbi-kose  5.Laambunni  6.kasaletenni  7. haqqetenni  8. kasheetenni  9. mu’rote agadinni  10. saadate obbinni  11.mine sagale diloosi’neemmo |  |  |
| 212 | Duucha wote sagale minenso, barandahonso woy wolu baxxino mine loosidhinanni? | 1. mine  2. wole baxxino mine  3. barandaho  4. woleno, xawisi------------------- |  |  |
| 213 | Baxxinohu sagale qishidhinanni mini noo’ne? | 1. eewa  0. dinonke |  |  |
| 214 | Galtinanni mini cira ayeho? | 1.Aneho   1. Baante gallanniho 2. Maatenni 3. Fiixu/ elunniho 4. Woleno xawisi………….. |  |  |
| 215 | Mini’nera maatekki goxate horoonsidhanno kifilla me’’e nooho? | -------------------------- |  |  |
| 216 | Minu giddoydo iimiidi raga (korniise) mayinni biifinsoonniho? | - - - 1. Kalaqamu haqqinni (biinfori dino, sabbunni,nna kandoonni sabbinni)       2. Calla anfi gede assinnoonniha (xu’minsoonnikki saattinni/plastic shara,shomboqqotenni/leemmichunni, haqqu xaawulinni, and komborsaatunni)       3. Xu’minsoonni korniise (siwiilunni/culku siwiilinni, haqqunni, alumineemetnni/ simintotenni |  |  |
| 217 | Mini uulliidi loosaminohu mayinniiti? | \| 1. Kalaqamu haqqinni (biinfori dino, sabbunni,nna kandoonni sabbinni) 2. Calla anfi gede assinnoonniha (xu’minsoonnikki saattinni/plastic shara, shomboqqotenni/leemmichunni, haqqu xaawulinni, and komborsaatunni) 3. Xu’minsoonni korniise (siwiilunni/culku siwiilinni, haqqunni, alumineemetnni/ simintotenni   (minxaafe) \| \| --- \| |  |  |
| 219 | Minu anni / ati saadate hoshsho, baatto hawuurranni saadanna lukkuwa ceo noosiho? | 1.eewa  0. dinoe | 222 |  |
| 220 | Aliidi saada giddonni meessi’nehu mageeshshaati?  Nookkiha ikkiro '00'.  95 nna woy roore ikkiro ‘95’.  Buunxoonnikkiha ikkiro '98'.   1. Ado saadanna bootta? 2. Wole saada? 3. Farado/harre/gaangootta? 4. Gaala/ 5. Me’’e? 6. Ge’reewo? 7. Caacurre/ lukkuwa? 8. Diishshote koshsha? | 1. Ado saadanna bootta? 2. Wole saada? 3. Farado/harre/gaangootta? 4. Gaala/ 5. Me’’e? 6. Ge’reewo? 7. Caacurre/ lukkuwa? 8. Diishshotekoshsha? |  |  |
| 221 | Saada galtannohu baxxino mini noo’ne? | 1.eewa  0.dinoke |  |  |
| 222 | Maatekkira meessi’neti/ki’neti loosidhinanni baatto noo’ne? | 1.eewa  0. dinonke | 224 |  |
| 223 | Maatekkira loosi’nanni baatto me’’e hekitaare ikkitanno? | ---------------------------Hekitaareeti |  |  |
| 224 | Maatekkira:   1. Korreente? 2. Raadoone? 3. Telewishiine? 4. Mine silke? 5. Kompiitere? 6. Mitore qiissanno udiinnichi? 7. Xarapheezzu? 8. Barcimu? 9. Shiwote daallasi? 10. Korreentete wolqanni sagale raisi’nanni mixashsho? 11. Laambunni/shaamu/?   Faanoose | Yes No   - - - 1. Korreente ……..1 0       2. Radoone……….1 0       3. Televishiine …….1 0       4. Mini bilbili……….1 0       5. kompiyuutere…….1 0       6. Motore qiissaancho...1 0       7. xarapheeza ………1 0       8. barcimu……………1 0       9. shiwote daallasi ….1 0       10. Siwiilu mixashsho…1 0       11. Laambu. Shaamu, faanoose lamp……………….1 0 |  |  |
| 225 | Maatekki mereero aanino udiinni noosari no:  a) Tolobishiine --------   1. Bilbilu------ 2. Shalleette------------ 3. Xexxerrisu------ 4. Saadate gaare- 5. Kaameelu/ hogowi kaameeli ----- 6. Yowolo motor nooti----- 7. Sasu goommi baajaaje ------------ | Yes No a) Televishiine . . . . . . 1 0  b) Mobayle …… . . . . . 1 0  c) Shalleette . . . . . . . . 1 0  d) Xexxerrisu. . ……….1 0  e) faradu/harrete gaare .1 0  f)kaameelu/hogowikaameeli..1 0  g) Yowolo motore nooti. . 1 0  h) sasu goommi baajaaje… 1 0 |  |  |
| 226 | Maatekkira baankete akkowaante kiiro noonsa? | 1. Eewa   1. Dinonke |  |  |
| 227 | Maatekki giddo shiimmaadda daddalu uurrinsha akkowaante kiiro noonsa? | 1. Eewa 2. Dinonke |  |  |

**Mojule III: Mashalaqqisaanonnita Sirote Gade**

| A.K | Xa’mubba | Dawarote Bebbeehache | sai/ kubbi | buuxo |
| --- | --- | --- | --- | --- |
| 301 | Adhamitta yannara me’’e diro ikkannohe? | 998. diqaagamannoe |  |  |
| 302 | Umi godowi gatihe yannara me’’e diro ikkannohe? | 998. diqaagamannoe |  |  |
| 303 | Godowu gate egenninohe? | 1. Eewa 2. Diegennoomma | 3 |  |
| 304 | Aliidi xa’mote kiiro dawarokki ‘’Eewa ‘’ ikkituro, 303, me’’e higge? | --------------------------- |  |  |
| 305 | Umo ka’e egennootta? | 1. Dee’ni 2. Eewa | 307 |  |
| 306 | Aliidi xam’mote kiirora 305, dawarokki ‘Eewa’ ikkituro, me’’e higge? | ---------------------------- |  |  |
| 307 | Ooso ilte egennootta? | 1. Dee’ni 2. Eewa | 311 |  |
| 308 | Aliidi xa’mote kiiro 306 dawarokki ‘’Eewa’ ikkituro, me’e higge? | ----------------------------- |  |  |
| 309 | Iloottari mereerinni lubbote noohu me’’eho? | ------------------------------ |  |  |
| 310 | Minekki ilootta qaaqquulli kiiro me’’eho? | --------------------------- |  |  |
| 311 | Tenne mule gatihe godowi yannara musi’roottati /xisso xaaddinohe? (Law. shuma giirannonna gaangaanna darshiishanno dhibba ) | 1. Dee’ni 2. Eewa |  |  |
| 312 | Maatekki mereero bikkiweelo mundeete xiiwo leeltinohu no? | 1. Dino 2. Eewa no | 3 |  |
| 313 | Aliidi xa’mo 312 dawarokki ‘’Eewa’’ ikkituro, isi ayeti? | 1. Anna’yaati 2. Ama’yaati 3. Meya rodoo’yaati 4. Ahaahe’yaati 5. Woleno xawisi……………….. |  |  |
| 314 | Godowu giddo ree ilamino qaaqqi noohe (godowamihunni 28 lamala? | 1. eewa  0. dinoe  998. diqaageemma |  |  |
| 315 | Qaaqquullu ilantay heedhe reyte egentinohe (mitte lamala wo’minsakkinni)? | 1. eewa  0. dee’ni  998. diqaageemma | gooffino |  |
| 316 | Aliidi xa’mote kiiro 314 dawarokki ‘’eewa ‘’ ikkitro, me’’e hige xaadinohe? | _____________ |  |  |
| 317 | Aliidi xa’mote kiiro 314 dawarokki ‘’eewa ‘’ ikkitro, qaaqqullu bushaawihehu dargu hiikkooti? | 1. mineeti  2. fayyimmate uurrinsha giddooti  3. wolewano xawisi _________ |  |  |

**Mojule IV: Amuwu Keeraanchimma Agarate Owaante**

| A.K | Xa’mubba | Dawarote bebbeehache | sai/kubbi | Buuxo |
| --- | --- | --- | --- | --- |
| 401 | Gedeno gatihe godowa mixo mixidheeti? | 1. eewa  0. dee’ni | 405 |  |
| 402 | Aliidi xa’mote kiiro 401 dawarokki ‘deeni’ ikkituro, kora ikkiheri maati? | 1. yannitte ilate damboowishshi doogga horoonsi’ra hoogate  2.Horoonsi’roommana diloossinoe  3. Woleno xawisi ………………… |  |  |
| 403 | Aliidi xa’mote kiiro 401 dawarokki ledde ‘dee’ni’ ikkituro, takkonta dibaxooma? | 1. eewa  0. dee’ni |  |  |
| 404 | Aliidi xa’mote kiiro 401 dawarokki ledde ‘dee’ni’ ikkituro, aganu gedensa ila hasi’ratta? | 1. eewa  0. dee’ni |  |  |
| 405 | Gedeni qaaqqi godowi gatihe yannara xisso macciishshantuhe hige? | 1. eewa  0. dee’ni | 407 |  |
| 406 | Aliidi xa’mote kiiro 405 dawarokki ‘’eewa’’ ikkituro, maati xissokki?  (doorsha nabbabboti) | 1. Akkimale siimunni du’nama   1. Bikku aliidi bisu iibbabbo 2. Siimu qooxeessi foole gana 3. Huxano 4. Damuume goola 5. Anga/ alba fuuga 6. Duucha yanna looqaleessa 7. Akkimale mundeete xiiwo 8. Woleno xawisi_________ |  |  |
| 407 | Gedeni qaaqqo godowitta yannara fayyimmate owaante afi’ritta? | 1.eewa  0. dee’ni | 409 |  |
| 408 | Aliidi xa’mote kiiro 407dawarokki ‘eewa’ ikkituro, towaanyote koru maati? | 1. Godowu gatanno yannara xaaddanno mitiimma  2. Godowu ledo xaado nookki fayyimmate qarra  3. Ilate albiidi buuxo  4. woleno (xawisi) ------------------ |  |  |
| 409 | Gedeni qaaqqi godowi gatihe yannara ANC buuxo assi’rootta? | 1. eewa  0. diaasi’roomma  998. diqaageemma | 425  425 |  |
| 410 | Aliidi xa’mote kiiro 409 dawarokki ‘eewa’ ikkituro, ANC buuxo me’’e higge assi’rootta? | ____________  998.diqaagamaae | 412 |  |
| 411 | ANC buuxo assi’rootta yanna aanti-gedeno agadhite xawisi? | 1^mi^ ANC ……………….aganira  2^ki^ ANC ………………aganira  3^kki^ANC………………..aganira  4^ki^  ANC……………….aganira  999. diqaageema |  |  |
| 412 | ANC owaante hiikkiinni afi’rattta? | 1. mootimmate hospitaalenni  2. hallanyu hospitaalenni  3. Halaale irkidhe kaa’litanno wolqa  4. Fayyimmate mereershi  5. hallanyu xaggate mine  6. Halaale irkidhino xagga mine  7. Fayyimmate keella  8. woleno xawisi) ________________ |  |  |
| 413 | Aliidi xa’mote kiiro 412 owwante mayra doodhitta? | 1. mini’yra mule ikkeenna  2. jawaata fayyimmate uurrinsha kalaqate  3. woleno xawisi _________ |  |  |
| 414 | Gedensi godowi gatihe yanna ANC buuxo assihehu ayeti? | 1.Dottorra   1. Nerse 2. Ilshiishaano meenti 3. Fayyimmate biiro 4. fayyimmate 5. budu ilshiishaano 6. wole xawisi |  |  |
| 415 | Gedeni qaaqqi godowi gatihe yanna Titaanosete kitiwate qasirota? | 1. eewa  0. deee’ni  998. diqaageemma |  |  |
| 416 | Aliidi xa’mote kiiro 415 dawarokki ‘eewa’ ikkituro, me’e higee? | ---------------------------------------- |  |  |
| 417 | Gedeni qaaqqi godowi gatihe yanna Laaboraatorrete miirmmara assirotaa? | 1. eewa  0. deee’ni  998. diqaageemma |  |  |
| 418 | Aliidi xa’mote kiiro 415 dawarokki ‘eewa’ ikkituro, hiitee? | 1= Mundee (Mundeete dana)  2= Mundeee (Shufuurrunita)  3= Jawa Shumma  4= Wayi Shumma  5= Wole xawisi |  |  |
| 419 | Gedeni qaaqqi godowi gatihe yanna ANC buuxo assir’rootta? | 1. eewa  0. deee’ni  998. diqaageemma | 425  425 |  |
| 420 | Aliidi xa’mote kiiro 419 dawarokki ‘eewa’ ikkituro, sokkate tajekkina? | 1= Unuuna Qansa  2= Busha Dano Amuwunnita  3= Maatete Damboowishshi Mixo  4= Ilate Owaante  5= Qaaqqu ilamay hee’re busha malaatta leellisha  6= Qaaqqu ilamay hee’reenna keeraanchimmasi agara  8= Woleno Xawisi……………………… | 421 |  |
| 421 | Hiittooha busha xissote malaate qummeessinihe?  (Doorsha nabbabbooti) | 1 = kaajjado umu damuume  2 = Mitiri illete lame ikka  3 = Qaaqqu godowu giddo godo’la giwa  4 = bikkiweelo mundeete xiiwo  5 = Alba darsha  6 = Anganna alba darsha  7 = huxano  8 = Sirote bisi mundee xooho  9 = boode wodanu xisso  l0 = aliidiri giddo dino |  |  |
| 422 | Aliidi xa’mote kiiro 421 te Busha xisso malaati mitiimma leeltuheta ha’rattara amaalihe? | 1.eewa  0. dee’ni  998. diqaagamaae |  |  |
| 423 | Gedeni qaaqqo hiikko ilattaro qummeessinoonnihe? | 1. eewa  0.dee’ni  998. diqaageemma | 428  428 |  |
| 424 | Aliidita xa’mote kiiro 420 dawarokki ‘’eewa’ ikkituro, hiikko ilatta gede kulloonihe? | 1. mine 2. fayyimmate keellira 3. fayyimmate mereershira 4. hallanyu xagga mine 5. meessi fajjo hakimewa 6. mootimmate hospitaalera 7. hallanyu hospitaalera 8. meessi fajjo hospitaalera 9. Woleno xawisi……………… |  |  |
| 425 | Ilitta wote ilshiishaahe ogeessa qeechinoonnihe? | 1. eewa  0. dee’ni  998. diqaagamaae | 427  427 |  |
| 426 | Aliidi xa’mote kiiro 425, dawarokki ‘’eewa’ ikkituro, aye ilshiishahera qeechinihe? | 1. Dottoru 2. Nerse//ilshiishaano/fayyimmate ogeeyye 3. Xeena ekistenshiine 4. Budu ilshiishaano 5. Woleno xawisi__________ |  |  |
| 427 | Ilate barri iilahera alba togoo qixxaawo assootta? | 1. hodhishsha  2. miinja suuqi’rootta  3. mundee aannoheha  4. mittoreno |  |  |
| 428 | ANC towaanyo assi’roottakiha ikkiro, korkaata kulattae?  (hassiisirodawarohalashshi)  (doorsha nabbabbooti) | 1. ANC lainohunni egennote anjewoy hoonge  2. keeraanchimmate qarri xaada hooga  3. fayyimmate uurrinsha galeemma mninni fafa  4. horoonsiraanote batinyinni  5. fayyimmate ogeeyye egennote anje  6. hodhishshu qarrini  7. fayyimmate uurrinsha mareemmata yanna hoogatenni  8. woleno xawisi ---------------------- |  |  |
| 429 | Fayyimmate uurrinsha giddo ilate mixidhe egennootta? | 1. eewa  0. deeni  998. diqaageema | 431 |  |
| 430 | Aliidi xa’mote kiiro 429 te dawarokki ‘dee’ni’ ikkituro, korkaata kulattae?  (dawaro hasiissuro duucha assi)  (doorsha nabbabbooti) | 1. Fayyimmate mine ilate daafira huwanyo hooga  2. fayyimma kisannori xaada hooga  3. fayyimmate uurrinsha mini’ya wiinni fa  4. fayyimmate ogeeyye egenno anje  5. hodhishshu qarra  6. woleno xawisi---------------------- |  |  |
| 431 | Gedeni qaaqqi ilami wote ateno ikki maatekki hiitte qixxaawo assitini? | 1. hodhishshunnita  2. womaashsha suuqi’ra  3. keeraaanchimma buuxi’ra owaante  4. mundee aannoha qixxeessi’ra  5. dandoo noosi ogeessa qixxeessi’ra  6. mittoreno |  |  |
| 432 | Gedeni qaaqqo hiikko ilitta? | 1. Mine   1. Fayymmate keellira 2. Fayyimate mereershira 3. Hallanyu kilinikera 4. Meessi fajjo kilinikera 5. Mootimmate hospitaalera 6. Hallanyu hospitaalera 7. Meessi fajjo hospitaalera 8. Woleno xawisi ……………… | 442 |  |
| 433 | Gedeni ilakki fayyimate uurrinshra iloottaha ikkiro, mamootenna mamote ha’runsatta? | 1. game hanaffo yannara  2. game hanaffuhunni 6-12 saate gedensaanni  3. game hanaffuhunni 13-18 saate gedensaanni  4. game hanaffuhunni 19-24 saate gedensaani  5. woleno xawisi __________ |  |  |
| 434 | Gedeni qaaqo ilita yaannara hiissiennahe ilitta? | 1. Mitii’mummakki iloomma  2. udiinnichu kaa’lonni iloomma  3. Godowa darreennae iloomma  4. woleno xawisi __________ |  |  |
| 435 | Gedeni qaaqqo ilshiishihehu ayeti? | 1.Dottoraho   1. Nersete 2. Ilshiishaanote 3. Dagate Fayyimmate ogeeyyeeti 4. Fayyimmate ekistenshineeti 5. Budu ilshiishaanooti (TBA) 6. Woleno xawisi___________ |  |  |
| 436 | Gedeni qaaqqo ilitta yannara mulekki ikke kaa’lamihehu noohe? | 1. Eewa 2. dee’ni |  |  |
| 437 | Aliita xa’mote kiiro 433te dawarokki ‘’eewa’’ ikkituro, ayeti ilitta yannara ka’lamihehu?  Wolurino hee’riro?  WO’MANTA TUMO HINKIILI | 1. ama’ya 2. ballo’ya 3. meyya rodoo’ya 4. beetti’ya beetto 5. wolootu balluwi’ya 6. wolootu annuwi 7. wolu fiixi firi 8. Neighbour 9. Woleno xawii…………………… |  |  |
| 438 | Ilitta yannara aante noo assootubba assinoonnihe? | 1. mundee aa  2. shota agatto  3. marfe  4. sirote biso dare halashsha  5. Ilate game rakkanno gede assanno xagicho |  |  |
| 439 | Marfe qasi’roottaro, maahooti?  (doorsha nabbabbooti )  BAALANTA TUMO HINKIILI | 1= game ajishate  2 = mundee uurrisate  3= huxano uurrissate  4= woleno xawisi)  999= dibadooma |  |  |
| 440 | Ilate yannara ga’manni mageeshsha keeshshannohe? | 1. 12 saate woro  2. 12- 24 saate  3. 25- 36 saate  4. 37 – 48 saate  5. 48 saate ale |  |  |
| 441 | Gedeno ilami qaaqqi hiittoohu ilami? | 1. lubbote noohu  2. bushaawinohu  3. fayyu ilame darganko reyno |  |  |
| 442 | Aleenni xa’mote kiiro 429 te, qaaqqo mine iloottaro, mine ila mayra doodhitta?  (doorsha nabbabbooti) | 1. boode yanna calla ga’minoehura  2. mule fayyimmate owaante nookkihura  3. hodhishshu mitiimma noo daafiraati  4. Hodhishshaho womaashshu hooginoehuraati  5. Fayyimmate ogeeyye laafa ikkitinohuraati  6. muli fiixi’yanna jaalla’ya nooehuraati  7. Owaante uynanni gara waajjeetii (Sirote bisi kifile halashsha lawinore)  8. fayyimmate uurrinsha fojo maaxawokkihuraati  9. fayyimmate uynanni owaante horo afa hoogeeti   1. Wole manni olluunnihu amaaleennaeeti   11. woleno xawisi ____________ |  |  |
| 443 | Gedini ila game yannara wole xisso xaaddinoheti no? | 1. ee  0. dino  999. diqaagamaae | 456 |  |
| 444 | Aliidi xa’mote kiiro 440te dawarokki ‘’ee’’ ikkituro, may xaadinohe?  (Do not read the choices) | 1 = akkimale sirote bisi mundee  2 = busha foole gana  3 = akkimale bisu iibbabbo  4 = qaaqqu balaxe anga woy lekka abba  5= Baby bad position/ mal presentation  6= seeda yanna game (> l2 saate )  7= hobbaate keeshsha  8 = Otoottote gurdama  9= Suuwo gobba higge fula  10 = Suuwo goowaho xaaxama  11= huxano  l2 = Bisu heleelamme xaada  l3= doorshu giddo dino | 456 |  |
| 445 | Hatte mitiimmara furgao woy hurre hasidhe egennootta?  Buuxo: hiikkonne mine heedhe assi’rootta gargarooshshe | 1. Eewa 2. Dee’ni | 456 |  |
| 446 | Aliidi xa’mote kiiro 442te dawarokki ‘’eewa’ ikkituro, xagga hiikkiinni hasi’rootta)?  AMA XAGGA HASIDHINOHA IKKIRO, MITTE DAWARO CALLA QOLI | 1.Mine   1. Fayymmate keellira 2. Fayyimate mereershira 3. Hallanyu kilinikera 4. Meessi fajjo kilinikera 5. Mootimmate hospitaalera 6. Hallanyu hospitaalera 7. Meessi fajjo hospitaalera 8. Woleno xawisi ……………… |  |  |
| 447 | Aleenni xa’mote kiiro 442te dawarokki ‘’eewa’’ikkituro, xagisihehu ayeti?  MITTE DAWARO CALLA HINKIILI | 1. Dottoru 2. Nerse 3. Ilshiishaano 4. Fayyimmate ogeeyye 5. Fayyimmate extenshine 6. Budu ilshiishaano (TBA) 7. Maate 8. Elunna emu 9. Qachu manni 10. Woleno xawisi___________ |  |  |
| 448 | Maroottawa mitiimma xaaddeennahe wole hospitaale qolle sonkoonnihe?  MINEETIRONNA GOBBAATIRO QORQORI | 1.Eewa   1. Dee’ni | 455 |  |
| 449 | Aleenni xa’mote kiiro 448 te, hiikka sonkihe? | 1. Fayyimmate keella 2. Fayyimmate mereersha 3. Hospitaale 4. Magano huuccitaariwa 5. Woleno xawisi…………………….. |  |  |
| 450 | Wolewa sonkiheha korkaata kultinohe? | 1. Eewa 2. Dee’ni | 451 |  |
| 451 | Aliidita xa’mote kiiro 447te dawarokki ‘’eewa’’ ikkituro, mayra wolewa sokkuheyya?  BAALANTA TUMO HINKIILI | 1 = Dare gowate udiinni hoonginni  2 = Bikkiweelo mundeete xiiwo  3 = Woyyaabbino buuxo assi’rate  4 = Dottorra hooggeenna  5 = Mundee tareessa  6 = Qaaqqu godowaho ofolla gari  7 = Mitu qaaqquulli kalaqami  8 = Qaaqqu cili amate otoottora  9 = Otoottote birxe fa’nama hooga  10 = Wolere xawisi  999 = Dafoomma |  |  |
| 452 | Hakkonne darga marootta? | 1. Eewa 2. Dee’ni   999. diqaageemma | 457 |  |
| 453 | Aliidi xa’mora dawarokki eewaancho ikkituro, mageeshshi yanna agadhite gargadhatta? | _______________ |  |  |
| 454 | Marte egennoottakki darga woshshante marittaro, macciishshammoheri no? | 1. Eewa 2. Dee’ni   999. diqaagamannoe |  |  |
| 455 | Sonkoonnihe darga ha’ra cee’mattahu? | 1 = Meentu busha dancha bada hoogatenni  2 = Minaanni huwata hoogatenni  3 = Owaantete dargi xeertinyinni  4 = Hodhishshu qarrinni  5 = Qaaqqu fayyimma hoonge  6= Bikka sa’no womaashsha baantannihura  7 = Hanqata owaante  8 = Hiikka mara noohero afootto  9 = Ha’rate yanna dinoe  10 = Womaashshu hoonginni  1 l = Woleno xawisi  999 = Diafoomma |  |  |
| 456 | Godowinni nootta yannara hodhishshaho doorsha assi’ratta? | 1. Eewa 2. Dee’ni |  |  |
| 457 | Suuqqi’rootta womaashsha horoonsidhe egennootta? | 1. Eewa 2. Dee’ni |  |  |
| 458 | Konne qaaqqo ilittahunni 6 ki lamala geeshsha xaadduhe mitiimma? | 1. eewa  0. dee’ni  999. diqaagamannoe | 460 |  |
| 459 | Aliidi xa’mote kiiro 458 te dawarokki ‘’eewa’’ ikkituro, maati xaaadiheri?  (doorsha nabbabbooti) | 1 = Akkimale mundee otoottote giddonni xooha  2 = Busha foole gana  3 = Bikka sa’ino bisu iibbabbo  4 = Qaaqqu unuunni giddollicho maaxama  5 = ‘Tetaanuse’  6 = Hobbaate keeshsha  7 = Kaajjado godowu xisso  8 = Huxano  9 = Gobbagino unuuna  10 = dawaro dino |  |  |
| 460 | 42 ki barra fayyimmate mini owaante afi’ratta? | 1. eewa  0. dee’ni  999. I don‘t remember |  |  |
| 461 | 457 ^ki^ xa’mora rakkino owaante afi’rattahu mamooteeti? | 1 ______ saate gedensaanni  2. ______ barrubba gedensaanni  3. ______ lamala gedensaanni |  |  |
| 462 | Aliidita xa’mote kiiro 457te dawarokki ‘’eewa’’ ikkituro, 42 barri giddo ilate gedeni owaante me’’e higge hasi’ratta | 998.Don’t know/remember | 412 |  |
| 463 | Umi yannara buuxo mama assi’ratta? | 1. Mine 2. Fayyimmate Keellira 3. Fayyimmate mereershira 4. Hospitaalete 5. Woleno xawisi……………… |  |  |
| 464 | Wole lexxote owaante noohe? | 1. Qaaqqoho kittiwaate aa  2. Maatete kiiro bikkaasincho mixo  3. Unuuna qansinanni gara amaala  4. woleno xawisi ____________ |  |  |
| 465 | Ilate gedeni owaante hiikkiinni afi’ratta? | 1.Fayymmate keellira   1. Fayyimate mereershira 2. Hallanyu kilinikera 3. Meessi fajjo kilinikera 4. Mootimmate hospitaalera 5. Hallanyu hospitaalera 6. Meessi fajjo hospitaalera 7. Woleno xawisi ……………… |  |  |
| 466 | Gedeni qaaqqira ilami wote ilate gedeni owaante uyhehu ayeti? | 1.Dottoraho  2. Nersete  3. Ilshiishaanote  4. Fayyimmate ogeeyyeeti  5. Fayyimmate ekistenshineeti   1. Budu ilshiishaanooti (TBA) 2. Woleno xawisi___________ |  |  |
| 467 | Galtekki hedo/ lao amate fayyimmate owaante hiittoote? | 1. Eewaanchote  2. Hi’naanchote  3. Diafoomma |  |  |
| 468 | Minikki maate yannitte fayyimmate mini owaante ayee la’annote ? | 1. Lamenka  2. Gashshaanna’ya  3. Anella  4. wole xawisi _____________ |  |  |
| 461 | Minikkira mule doogote injo noohe? | 1.Eewa   1. Dinoe |  |  |
| 670 | Qarichimmate qajeelsha maatekki adhitino? | 1. Eewa 2. Dee’ni |  |  |

**Mojule V: Amuwu Fayyimma Owaante Lainohunni Egennonna Laooshshe Xiinxallo Assinoonni Qooxeessira**

**1. Amuwu Fayyimma La’nohunni Assinanni Egennote Xiinxaawo**

| A.K | Xa’mubba | Dawarote bebbeehache | sai/kubbi | buuxo |
| --- | --- | --- | --- | --- |
|  | **Ilate balaxote buuxo lainohunni egenno buuxa** |  |  |  |
| 501 | Ilate balaxote buuxo owaante maatiro afootta? | 0. Dee’ni  1.Eewa | 503 |  |
| 502 | Aleenni xa’mote kiiro 501te dawarokki ‘’eewa’’ ikkituro, ilate balaxote buuxo amuwu otootto keeraanchimmara horo noose? | 1. Dee’ni 2. Eewa |  |  |
| 503 | Umi doycho ilate balaxote buuxo towaanyo xaphi yitannohu mamooteeti? | 1.16^ki^ lamala sa’eenna   1. 16^ki^ lamala wo’mikki |  |  |
| 504 | Xaphooma ilate balaxote buuxo towaanyo meyati godobbe ilta geeshsha noota afootta? | 1.4nni ajjanno   1. 4 nna hakkuy ale |  |  |
| 505 | Umiti woyyaabbino ilate balaxote buuxo towaanyo assi’roottahu hiikkooti? | 1. Fayyimmate keellira 2. Fayyimmate mereershira/hospitaalete |  |  |
| 506 | Ilate balaxote towaanyo yannara godowii noo ama labaratorete buuxo assidhanno? | 1. Dee’ni 2. Eewa |  |  |
| 507 | Ilate balaxote towaanyo yannara godowii noo amara xagga hasiissanno? | 1. Dee’ni 2. Eewa |  |  |
| 508 | Ilate balaxote towaanyo yannara godowii noo amara amaalete kaa’lo hasiissanno? | 1. Dee’ni 2. Eewa |  |  |
|  | **Fayyimmate Uurrinsha Giddo Ilate Mayimma Lainohunni Shiqqino Xa’mubba** |  |  |  |
| 509 | Fayyimmate uurrinshara ilate hornyi maatiro afootta? | 0.diafoomma  1. eewa |  |  |
| 510 | Allidita xa’mote kiiro 501te dawarokki ‘’eewa’ ikkituro, uurrinshate gidoo ila amuwahonna ilantanno qaaqquullira hornyaamete? | 1. Dee’ni 2. Eewa |  |  |
| 511 | Hiitte manchooti Fayyimma uurrinsha ilatta kultinoheti? | 1. Danotenni  2. Baalu |  |  |
| 512 | Ilate seyanno dargi hiikkooti? | 1.Mineeti   1. Fayyimmate uurrinsha |  |  |
| 513 | Qaaqquulle ilate injo qineessora ayeti dandoo noosihu? | 1.Anfoonnikki manni (TBA), elunna emu , jaalto/jaala   1. Fayyimmate mini ogeeyye |  |  |
| 514 | Ilate yannara xaaddinose xissonni mannu aalo ikkitino ama no? | 0. Dino  1. Eewa |  |  |
| 515 | Qajeeltino fayyimmate ogeeyye amuwu rewo gargartanno yite hedatta? | 1. Dee’ni   1. Eewa |  |  |
|  | **Ilate gedeni towaanyo Lainohunni Egenno (Huwanyo ) Badate Xa’mubba** |  |  |  |
| 516 | Ilate gedeni towaanyo daafira afoottari no? | 1. Dino 2. Eewa |  |  |
| 517 | Aliidi xa’mote kiiro 501te , dawarokki ‘’eewa’’ ikkituro, Ilate gedeni amuwunna qaaqquullu keeraanchimmara horaameettete | 1. Dee’ni 2. Eewa |  |  |
| 518 | Ilate gedeni towaanyo me’’e hinge assi’nanni agarranniro afootta? | 1. 3nni ajjanno   1. 3 nna hakkuy ale |  |  |
| 519 | Umi yannara ilate gedeni towaanyo assate tolanno dargi hiikkooti? | 1. Fayyimmate keellaati 2. Fayyimmate mereersha / hospitaalete |  |  |
| 520 | Godowii noo ama PNC towaanyo yannara wole lexxote amaale hasidhanno? | 0.Dee’ni  1. Eewa |  |  |
| 521 | Ilate gedeni yannara xaaddanno mitiimmanni ama reytanno yite hedatta? | 0. Dee’ni   1. Eewa |  |  |
| 522 | Ilate gedeni towaanyo assi’ra amuwu rewo gargartanno yite hedatta? | 1. Dee’ni 2. Eewa |  |  |

1. **Amuwu Fayyimmate Owaante Daafira Beeqqaanote Noonsa Lao Badate Shiqqino Xa’muwa**

1-takkonta sumuu yaa giwa; 2- sumuu yaa giwa 3- mereerima; 4- sumuu yaa ;

5- Addanko sumuu yaa

| A.K | Xa’mubba | 1 | 2 | 3 | 4 | 5 |
| --- | --- | --- | --- | --- | --- | --- |
|  | **Laote xa’mubba Ilate balaxote** **lainohunni** |  |  |  |  |  |
| 523 | Ilate balaxote towaanyo amuwahonna qaaqqu suuwora horaameessa ikkasira mageeshsha sumuu yaatta? |  |  |  |  |  |
| 524 | Umi doycho Ilate balaxote towaanyo 16 lamalanni ajjannoha ikkiro, mageeshsha sumuu yaatta? |  |  |  |  |  |
| 525 | 4 calla hige Ilate balaxote towanyo assi’ra gatino godowira ikkitanno; yitanno hedora mageeshsha sumuu yaatta? |  |  |  |  |  |
| 526 | Umi doycho Ilate balaxote towaanyo assi’rate injaanno dargi fayyimmate mereershaati/ hospitaalete yitanno hedora mageeshsha sumuu yaatta |  |  |  |  |  |
| 527 | Ilate balaxote towaanyo yannara godowinni noo amuwi labaratorete buuxo assi’ra noonsata mageesshsha sumuu yaatta? |  |  |  |  |  |
| 528 | Ilate balaxote towaanyo yannara godowinni noo amuwi wole hasiissanno xagga qixxeessinansara hasi’rannota mageeshsha sumuu yaatta? |  |  |  |  |  |
| 529 | Ilate balaxote towaanyo yannara godowinni noo amuwi wole hasiissanno amaale uynansara hasi’rannota mageeshsha sumuu yaatta? |  |  |  |  |  |
| 530 | Amuwu fayyimmate owaantera Ilate balaxote towaanyo assi’rannokkihu, womaashshu fulo batidhanno yee hedayti yitanno hedo mageeshsha irkisatta? |  |  |  |  |  |
| 531 | Amuwu fayyimmate owaantera Ilate balaxote towaanyo assi’rannokkihu, fayyimmate mini ogeeyye mishshannonke yee hedayti yitanno hedo mageeshsha irkisatta? |  |  |  |  |  |
|  | **Laote Xa’mubba Fayyimmate Uurrinsha Giddo Ila Lainohunni** |  |  |  |  |  |
| 532 | Amuwahono ikko ilamawo qaaqqira addinta jawa horo noose |  |  |  |  |  |
| 533 | Amuwu fayyimmate uurrinshara ila danchate yite mageeshsha hedatta? |  |  |  |  |  |
| 534 | Fayymmate uurrinshara qaaqquulle danchate yite hedatta? |  |  |  |  |  |
| 535 | Fayyimmate ogeeyye qajeeltinota ikkansa mageeshsha sumuu yaatta? |  |  |  |  |  |
| 536 | Ilate yannara babbaxxitino mitiimma rewoteno iillisha dandiitanno? |  |  |  |  |  |
| 537 | Qajeeltino fayyimmate ogeeyyenni qaaqquulle ilshiisha amuwu rewo gargartanno? |  |  |  |  |  |
| 538 | Ganto godowi keeraanchimma agarate mixo fushshi’ra hasiissanno? |  |  |  |  |  |
| 539 | Labbaahu fayyimmate ogeeyyenni ila saalaho woy seerimalete yite hedatto? |  |  |  |  |  |
| 540 | Ilshiinshanni daallasira miteekkite game gadadissuro, disaallanni? |  |  |  |  |  |
| 541 | Amuwu fayyimmate mine ila giwannohu, womaashshu fulo batidhanno yee hedayti yitanno hedo mageeshsha irkisatta? |  |  |  |  |  |
| 542 | Fayyimmate ogeeyye amuwa ilshiishshanno yanna ayirrinye diuytanno yinanni hedo mageeshsha sumuu yaatta? |  |  |  |  |  |
|  | **Laote Xa’mubba Ilate Gedeni Towaanyo Daafira** |  |  |  |  |  |
| 543 | Ilate gedeni towaanyo amuwahonna daaimaho danchate ikkase mageeshsha sumuu yaatta? |  |  |  |  |  |
| 544 | 3 calla hige Ilate gedeni towanyo assi’ra ilate yannara xaaddanno mitiimma hunate ikkitanno; yitanno hedora mageeshsha sumuu yaatta? |  |  |  |  |  |
| 545 | Umi doycho Ilate gedeni owaanyo assi’rate injaanno dargi fayyimmate mereershaati/ hospitaalete yitanno hedora mageeshsha sumuu yaatta |  |  |  |  |  |
| 546 | Godowii noo amuwira Ilate gedeni owaanyo assinanninsa yannara baxxitino amaale hasiissannonsa? |  |  |  |  |  |
| 547 | Ilate gedeni towaanyora xaaddannose mitiimmanni ama reytannota mageeshsha sumuu yaatta? |  |  |  |  |  |
| 548 | Ilate gedeni towaanyo fayyimmate uurrinshara amuwu rewo gargartanno yite mageeshsha hedatta? |  |  |  |  |  |
| 549 | Amuwu fayyimmate owaantera PNC towaanyo assi’rannokkihu, womaashshu fulo batidhanno yee hedayti yitanno hedo mageeshsha irkisatta? |  |  |  |  |  |
| 550 | Amuwu fayyimmate owaantera PNC towaanyo assi’rannokkihu, fayyimmate mini ogeeyye mishshannonke yee hedayti yitanno hedo mageeshsha irkisatta? |  |  |  |  |  |

**Mojule VI: Dagoomi-budu Himano**

| A.K | Xa’mubba | Tumi-qolote bebbeehache | sai/kubbi | Buuxo |
| --- | --- | --- | --- | --- |
| 601 | Qarqari’nera nooha dagoomi-bude Amuwu fayyimmate owaante heekki assinori maati?  (doorsha nabbabbooti)  BAALANTA TUMI-QOLO HINKIILI | 1. Budu xagissaano hee’ranna iillishanno hekko  2.Amma’note xagissaano hee’ranna iillishanno hekko  3.Budunni ilshiishanno manni qooxeessaho hee’ra attendants  4.Maassamino way  5. Amu fayyimmate owaante horoonsha waajja  6.Waaxishshu hekko  7. MHSU lainohunni qooxeessu mdagoomira galtanno huwanyo  8. Gashshaanni iillishanno hekko  9. Balluwu ayiddi hekko  10. Woleno xawisi …………. |  |  |

**Modele VII: Ilaannaho Uynanni Fayyimmate Owaante Horo Isilanchimara Noonsa Egenno Keennanni Borri-Xa’mo**

**Ilate Balaxote Owaataasinu Harunso Silanchimma Daafira Shiqqino Borri-xa’mo**

1-lowo geeshsha diduushshanno.; 2- diduushshanno 3- mittoreno yaa didandeemmo; 4- danchaho 5- lowo geeshsha danchaho.

|  | **Xa’mo** | 1 | 2 | 3 | 4 | 5 |
| --- | --- | --- | --- | --- | --- | --- |
| 1 | Fayyimmate ogeeyye ledo noohe miiloomikki hiittooho, ilate balaxote harunsokki yannara? |  |  |  |  |  |
| 2 | Fayyimmate ogeeyye ledo noohe miiloomi guutinokkiha ikkiro, hiittoo ogeessaati yite hedatta? |  |  |  |  |  |
| 3 | Fayyimmate ogeeyye ganyite uytanno owaante hasi’ratta woyte illacha tuge loosanno |  |  |  |  |  |
| 4 | Fayyimmate ogeeyye, ilate balaxote harunso yannara ikkado yanna, uytanno yaatta towaanyokki yannara fayyimmate urrinshara ate hedora? |  |  |  |  |  |
| 5 | Fayyimmate ogeeyye owaante uytanno yannara teeda agarate gade ilate balaxote owaante uynanni kifilera ate hedora hittooho yaate? |  |  |  |  |  |
| 6 | Fayyimmate ogeeyyera hedokki xawisate noonsa gade ate hedora hittoote? |  |  |  |  |  |
| 7 | Ate hedo gumulora beeqqootta ikkiro hiittoonni? |  |  |  |  |  |
| 8 | Ilate balaxote harunsokki yannara, owaante afi’rate towaanyo assate agadhinanni yanna dikeeshshinanni yite hedatta? |  |  |  |  |  |
| 9 | Fayyimmate uurrinsha wo’mankawa co’ittete ate hedora? |  |  |  |  |  |
| 10 | Aterano daaimahono uynoonni fayyimmate owaataasinenni afi’rootta kaa’lo hiittoote? Lawishshu gede ilate yannara atenna daaimu co’ima agaratenni? |  |  |  |  |  |
| 11 | Ilate balaxote harunsora uynanni yannara fayyimmate taje ikkadote yite hedatta? |  |  |  |  |  |
| 12 | Fayyimmate ogeeyyewinni ikkado taje uynanni ilate yine hendanni yannana godowu gatinkunni me’’e agana ikkiro, ikkadote yite hedatta? |  |  |  |  |  |
| 13 | Ikkado taje afi’roomma yite hedatta, godowaho busha malaatta bade afate widoonni? |  |  |  |  |  |
| 14 | Ate hedonni fayyimmate ogeeyye lifixxinote fayyimmate qara tirate? |  |  |  |  |  |
| 15 | Ate hedonni ikkado tajenna xawishsha afi’roomma yite hedatta wo’munku fayyimmate qarrira? |  |  |  |  |  |
| 16 | Ate hedora, ilate balaxote harunso assi’nanni kifile co’itete? |  |  |  |  |  |
| 17 | Ate hedora, taaltino sagale saga’late ikkado taje afi’rooma yite hedatta? |  |  |  |  |  |
| 18 | Ate hedora, TT (titanesete dhiba gargaranno kitiwaati) daafira ikkado taje noohe? |  |  |  |  |  |
| 19 | Ate hedora, mundeete dani buuxo daafira ikkado taje nooe yite hedatta? |  |  |  |  |  |
| 20 | Ate hedora, shumate buuxo daafira ikkado taje nooe yite hedatta? |  |  |  |  |  |
| 21 | Ate hedora, kiilogiraamekkinna seendillikki bikko daafira ikkado taje noohe? |  |  |  |  |  |
| 22 | Ate hedora mundeekki xiiwo bikkora ikkado taje nooe yite hedatta? |  |  |  |  |  |
| 23 | Ate hedora, ikkadu xawishshi noohe uynannihe dinyera? |  |  |  |  |  |

**Konni Woroonni Noo Borri-xa’mo Ama Ikkadonna Silancho Owaante Illtu Yannara Afi’rase Keennanni Xa’mo**

1-lowo geeshsha sumuu diyeemma 2- sumuu diyeemma; 3- mittoreno diyeemma; 4-sumuu yeemma 5- lowo geeshsha sumuu yeemma

| A.K | XA’MUWA | 1 | 2 | 3 | 4 | 5 |
| --- | --- | --- | --- | --- | --- | --- |
| 1 | Mule’ne noo fayyimmate uurrinshara owaante afi’nara agadhinanni baychi danchaho yee hedeemmo |  |  |  |  |  |
| 2 | Amuuwu kifilera, no shummate bale co’itete yee heedeemma mule noo’ne fayyimmate uurrinshara |  |  |  |  |  |
| 3 | Mule no fayyimmate urinshara, anganna biso hayishi’nanni dargi no. |  |  |  |  |  |
| 4 | Mule no fayyimmate uurrinshara kuriuu labbannori no, korreentenna way. |  |  |  |  |  |
| 5 | Amuwu kifile e’nanni waalchinni hananfe mittore qarramikkinni/shotuniititi. |  |  |  |  |  |
| 6 | Mule no uurrinshara, fayyimmate ogeeyye uytanno gametenna ilate yanna kaa’lo danchate. |  |  |  |  |  |
| 7 | Mule no uurrinshara, fayyimmate ogeeyye uytanno daaimu/marqu keeranchimma daafira danchate. |  |  |  |  |  |
| 8 | Mule no uurinshara, fayyimmate ogeeyye uyitanno kaa’lo xissote yannara danchate. |  |  |  |  |  |
|  | | | | | | |
| 9 | Mule no fayyimmate uurinsha mageeshshi yanna adhanno lekkatenni? | ________sa’ate | | | |  |
| 10 | Hodhishshu owaante afidhinanni fayyimmate uurinsha hadhinanni woyte. | 1. Ee  0. Dee’ni | | | |  |
| 11 | Mule no fayyimmate uurrinshara, owaante afi’rate baantanni. | 1. lowo batooshshe  2. mereerima  3. shiima | | | |  |

**Konni Woroonni Noo Borri-xa’mo Keennannihu Silancho Owaante Ilate Gedensaanni Noo Yannara Fayyimmate Uurrinshshara**

1-lowo geeshsha diduushshanno; 2- diduushshanno; 3- mittorenno diyeemma; 4- danchaho 5- lowo geeshsha danchaho

|  | **Xa’muuwa** | 1 | 2 | 3 | 4 | 5 |
| --- | --- | --- | --- | --- | --- | --- |
| 1 | Fayyimmate ogeeyye ledo noohe miiloomikki hittooho, ilate balaaxxote harunsokki yannara? |  |  |  |  |  |
| 2 | Fayyimmate ogeeyye ledo noohe miiloomi guutinokkiha ikkiro, hittoo ogeessaati yite hedatta? |  |  |  |  |  |
| 3 | Fayyimmate ogeeyye ganyite uytanno owaante hasi’ratta woyte illacha tuge loosanno. |  |  |  |  |  |
| 4 | Ate hedora, xisso gargarate uyinanni xagichi no, ilate gedensiidi yannara. |  |  |  |  |  |
| 5 | Fayyimmate ogeeyye owaante uytanno yannara teeda agaratenna ayirrisate gade ilate gedensaanni uyinanni owaante kifilera , ate hedora hittooho yaate |  |  |  |  |  |
| 6 | Ate hedora,fayyimmate ogeeyye ledo hedokki xawisate hasaawatta |  |  |  |  |  |
| 7 | Ate hedora,fayyimmate ogeeyye maatekkira leellishshino albi danchaho |  |  |  |  |  |
| 8 | Ate hedora, qaaqqu ilame ka’’anni hee’reenna unuunna qansate orte hiittoote ilanni kifilera. |  |  |  |  |  |
| 9 | Ate hedora, fayyimmate ogeeyye uytino owaante daaimu keraanchimmara ka’litanno. |  |  |  |  |  |
| 10 | Ate hedora, afirootta owaante fayyimmate ogeeyyewinni fayyimmakkira lowo kaa’lo afidhino lawishshu gede ilate gedensaanni uynanni bisikki co’imara. |  |  |  |  |  |
| 11 | Ate hedora, ikkado taje nooe yite hedatta qaaqqu keeraanchimmara/fayyimmara. |  |  |  |  |  |
| 12 | Ate hedora ikkado taje nooe yite hedatta ragunni unuuna qansate hayyora? |  |  |  |  |  |
| 13 | Busha ilate widoonni dagganno malaatta bade afate daafira ikkado taje nooe yite hedatta ilate gedensiidi yannara, amaate keraanchimmaranna qaaqqu fayyimmara. |  |  |  |  |  |
| 14 | Ate hedora, fayyimmate ogeeyye uytanno owaante lifixxinote qaqqu fayyimmara. |  |  |  |  |  |
| 15 | Ate keraanchimara uynanni owaantera fayyimmate ogeeyyenni fayyimmte qarra bade afatenna tirate widoonni. |  |  |  |  |  |
| 16 | Aye hajorano ikkado xawishshi nooe yite hedatta. |  |  |  |  |  |
| 17 | Ate hedora fayyimmate mini co’imma lainohunni. |  |  |  |  |  |
| 18 | Ate hedora shumate mininna hayishshi’nanni kifilla co’imma daafira. |  |  |  |  |  |
| 19 | Ate hedora, ilate gedensaanni owaante afi’nanni baychi ikkadoho. |  |  |  |  |  |
| 20 | Ate hedora owaataassinete ikkadu baychi no, xiwamaassinete ledo xaadinohunni. |  |  |  |  |  |
| 21 | Ate hedora, ikkadu daallasi no gamete/ilanni kifilera. |  |  |  |  |  |
| 22 | Ate hedora, ikkado ogeeyye noo kaa’litanohe gede. |  |  |  |  |  |
| 23 | Ate hedora ilate gedensaanni afi’nanni woyte ikkado baychi no fooliishshi’rate mittu qarri nookkiha (caabbichu, hurote. kifilete loonsannirichinni). |  |  |  |  |  |

**Mojule IIX: Ilate Albiidi yanna, Illanni yannaranna ilate gedensaanni xaadanno qarri daafira keenanni borri-xa’mo.**

| A.K | Xa’muwa | Dawaro | Sai | qaagiishsha |
| --- | --- | --- | --- | --- |
| 801 | Godowinni hee’neenna fayyimmate ogeeyyewiinni kaa’lo hasi’rano qarri maatiro ballo kulattae?  Wolunna?  (Mittunni aleenni doora dandinanni.) | 1= deerra sa’ino umu damuume.  2= Mitiri illete la’nanni woyte duuccha ikka.  3=qaaqqu godowu giddo milli yaa hooga/aja  4= mundeete xiiwo lexxa  5= alba darsha.  6= albanna lekka darsha.  7= huxi’ra.  8= otoottote widoonni mundaa.  9= mudukku woroonni deerra aleenni xissa.  l0=aleeni noori doorshi giddo dino. |  |  |
| 802 | Xaa kayinni kulattaeri, illanni yannara fayyimmate ogeeyyewiinni xagisi’ra hasi’ranno qarri maatiro ballo kulattae?  Woluna?  (Mittunni aleenni doora dandiinanni) | 1 =Deerra sa’ino otoottote mundaa.  2 = Otoottotenni busha foole afi’rinori fula.  3 = Deerra sa’ino bisu iibbilli.  4 = Ilamanno qaaqqi anga woy lekkate widoonni daa.  5=Ilamano qaaqqi maqqaalame/ragiweelo daa.  6. Illanni yannara 12 saate aleenni godowu game/gamaa’ma.  7= Keere tidhama hooga.  8 = Otoottote gurdaama.  9= Suuwo, qaaqqo balaxxe otoottotewinni fula.  10=Suuwo qaaqqu goowira xaaxante fula.  11= Bisu huxi’ranno yannara.  l2 = bisu heleelama.  l3= dawaro dino. |  |  |
| 803 | Qaaqqo ama ilte ka’anni hedheeenna fayyimmate ogeeyyewiinni xagisi’ra hasiissanno qarri hiikkonneeti?  Woluna?  (Mittunni aleenni doora dandiinanni) | 1 = Derra sa’ino otoottote mundaa.  2 =Otoottotenni bushu foole afi’rinori fula.  3 = Deerra saino bisu iibbilli.  4 = amate ununnu qacce giddo hige ea .  5 = Tetaanese  6 = Keere tidhama hooga  7 = mudukku woroonni deerra aleenni xissa.  8 = Bisu huxi’ranno yannara  9 = Amate unuuni darsha.  10 = dawaro dino |  |  |

**Mojule IX: Ilatenna Ille Ka’ni Gedensaanni Daanno Qarri Balaxote Qixxaawote Rosicho**

| A.K | Xa’mo | Dawaro | Sai | qaagiishsha |
| --- | --- | --- | --- | --- |
| 901 | Godobbe heedhe ikko ilattara albaanni ati woy maatekki mixidhinoonnihu hiikko mine ilateeti? | 1= Ee  2= Dee’ni  999= Diqaageemma. | 906 |  |
| 902 | Ee ikkiro xa’mo 901, hiittenne mixo mixidhinoonni ati woy maatekki?  (Doorshsha nabbabooti!  Wolu no?  Dawaro qoqqowi | l= Ilate mule no fayyimmate uurrinsha doodhoomma  2= Qajeeltino fayyimmate ogeeyyenni ileemma yee mixiroomma  3= womaashsha suuqiroomma , sorranniere qixxeess’iroomma  4=ikkannoha hodhishshu dana doodhooma  5=Mundee’ya dani ay ledo xaadannoro afi’re noomma aannoeha |  |  |
| 903 | Ilatta uurrinsha, hiikkiicho doodhita mixokkira?  Nabbabbooti doorsha! | 1. Mine  2. Fayyimmate keellira  3. Fayyimmate agarooshshira  4. Hosipitaalete  5. Wole xawisi……………… |  |  |
| 904 | Ogeeyye doodhoottaha ikkiro ay iillishihe?  Nabbabbooti doorsha! | 1. Dottoru  2. Nerse/ilshiishaano ogeeyye  3. Xeena ekistenshine  4. Budu ogeeyye  5. Wole xawisi______________ |  |  |
| 905 | Hodhishshaho dodhootta ikkiro, ati doodhootta hodhishshinni haratta godowinni heedhe? | 1. Ee  0. Dee’ni |  |  |
| 906 | Womaashsha suqqirootta ikkiro, godowinni hedhe horonsi’roota? | 1. Ee   1. Dee’ni |  |  |

**Ha’runsokkira galaxxeemmo!**
