# Supplementary material for: Effect of community health education on mothers’ knowledge of obstetric danger signs and birth preparedness and complication readiness practices in southern Ethiopia: A cluster randomized controlled trial
Source: PLoS One. 2024 Nov 27;19(11):e0312267. doi: 10.1371/journal.pone.0312267 (PMC11602057; doi:10.1371/journal.pone.0312267)
Supplement: S5 File — (DOCX) [file pone.0312267.s005.docx]

Improving maternal health service utilization among women of reproductive age in Sidama Regional State, Ethiopia: A Cluster Randomized Controlled Trial

Investigator: Amanuel Yoseph (BSc in Public Health, MPH)

May, 2022

**Summary**

**Introduction:** Maternal health service (MHS) utilization helps to decrease maternal morbidity and mortality. However, the utilization of MHS is very low in Ethiopia and a complex phenomenon affected by several predictors. Its predictors, barriers, and facilitators differ from region to region in Ethiopia, and existing evidence is not sufficient to design effective and efficient strategies. One of the methods to increase the utilization of maternal health care is improving the maternal knowledge about the obstetric danger signs, skills of birth preparedness, and complication readiness practice using health education. However, the effect of health education on these outcomes had not been comprehensively explored and the presently existing studies reported controversial results.

**Objective:** This study aims to improve maternal health service utilization among women of reproductive age in Dale and Wonsho districts of the Sidama region, Ethiopia.

**Methods:** A community-based cross-sectional study will be carried out to assess the utilization and predictors of maternal health care. A phenomenological qualitative study will be conducted to explore perceptions, barriers, and facilitators of maternal health care. A cluster randomized controlled trial will be employed to assess the effect of health education on maternal knowledge about the obstetric danger signs, birth preparedness and complication readiness plan, and maternal health service utilization (MHSU). The minimum required sample size is 1,104 for a cross-sectional study. To explore perceptions, barriers, and facilitators of maternal health care a minimum of 20 in-depth interviews, 3 focus group discussions per group, and 20 key informative interviews will be conducted. The minimum required sample size to assess the effect of health education on outcomes is 1,126. A multi-stage sampling method will be used to select the study participants for a cross-sectional study. A maximum variance sampling method will be used for the qualitative study. A cluster-randomized sampling method will be utilized for the interventional study. Quantitative data will be collected using a validated, pre-tested, and structured questionnaire. Qualitative data will be collected using pre-tested focus group discussions, in-depth interviews, and key informant interviews guides until information saturation is reached. The intervention group will be received 12 rounds of pre-recorded audio-based health education. The utilization of maternal health care will be measured at baseline and 6 months after the intervention. Data will be entered into Epi Data version 3.1 and exported to the SPSS and Stata software for analysis. Descriptive analyses will be carried out to get descriptive measures for the important variables of interest. The generalized estimated equations analysis will be used to assess the potential predictors of MHSU. The coding and analysis of the qualitative data will be done using the thematic content analysis technique by Atlas-Ti software and presented in narratives. For maternal knowledge about the danger signs and practice of birth preparedness and complication readiness, the multilevel linear regression models will be utilized. The intention-to-treat analysis will be used to compare outcomes between groups. Paired and independent t-tests will be used to compare the effect of an intervention. Statistical significance level will be adjusted to account for the effect of multiple comparisons problems using the Bonferroni corrections methods.

**Schedule and budget:** This study will be conducted from October to August 2023; the total cost of the project will be **421,600 ETB (7,026.67 EURO)**.

**Keywords:** Health education, maternal health service, utilization, women of reproductive age, Dale and Wonsho districts, Ethiopia.

**Introduction**

The highest number of maternal mortality, particularly in developing countries is due to low-level utilization of existing maternal health service (MHS) [[1](#_ENREF_1)]. Prior studies in Ethiopia showed that MHSU is very low in general and a significant regional and urban/rural difference exists [[2-7](#_ENREF_2)]. For instance, 43% of pregnant mothers in Ethiopia had 4 or more antenatal care visits whereas 48% gave birth at the health facility, and merely 34% of women received postnatal care within 48 hours after childbirth [[3](#_ENREF_3)] . Likewise, the utilization of maternal and neonatal healthcare services during illness is 6%, 2%, and 3% for pregnant women, postpartum women, and neonates in the Wonago district of Southern Ethiopia, respectively [[4](#_ENREF_4)].

Furthermore, great differences in maternal death happen between the national regional states in Ethiopia. For instance, it is ranged from 74 in the Tigray regional state to 548 mortalities per 100,000 live births in the Afar region [[8](#_ENREF_8)], which briefs the requirement to further study the landscape of utilization patterns of maternal health services at the limited local settings.

The various predictors contributed to the use of the existing MHS and can be categorized as socio-economic and demographic, behavioral, cultural practices and beliefs, organizational, service quality linked predictors, health system functioning, and the lack of transport in rural settings [[2](#_ENREF_2), [4-6](#_ENREF_4), [9-11](#_ENREF_9)]. However, these earlier studies that investigated the utilization of MHS in Ethiopia, focused mainly on individual-level predictors with limited consideration to community-level and contextual predictors. Also, these studies were only quantitative cross-sectional and recommended conducting a further study using a qualitative method to clearly understand the perceptions, barriers, and facilitators of MHSU. These studies also not utilized a multilevel regression model. The predictors, barriers, and facilitators of MHSU differ from region to region in Ethiopia, and utilization of MHS are highly variable and existing evidence is not satisfied to design effective and efficient strategies [[2](#_ENREF_2), [4-6](#_ENREF_4), [9-11](#_ENREF_9)].

The community-level health education intervention (CLHEI) is a fundamental approach to increase maternal knowledge regarding the obstetric danger signs, the practice of birth preparedness as well as complication readiness for emergency complications and MHSU [[12-19](#_ENREF_12)]. However, the effect of a CLHEI on MHSU had not been comprehensively explored and the currently existing literature or evidence is contradicting or reported mixed findings. The several observational and interventional studies conducted in the Sokoto state, Nigeria [[20](#_ENREF_20)], Korogwe district of rural Tanzania [[21](#_ENREF_21)], Kwara state of Nigeria [[22](#_ENREF_22)], Mundri East County, South Sudan [[23](#_ENREF_23)], Alimosho, Lagos State of Nigeria [[24](#_ENREF_24)] and Spain [[25](#_ENREF_25)] showed positive effects.

On the contrary, the interventional studies conducted in Nepal [[26](#_ENREF_26)] and Latin America found that no significant effect of the health education intervention on the perinatal outcomes and use of health facilities service [[27](#_ENREF_27)]. Moreover, a study from community antenatal clinics showed that a general service for initiation of breastfeeding using peer support workers was unsuccessful in enhancing initiation rates of breasting feeding [[28](#_ENREF_28)]. Also, these entire studies do not offer a satisfactory appreciation of the effect of a CLHEI on the outcomes due to method limitations (e.g. using a purposive sampling method, absence of randomization and appropriate control group, lack disaggregation of effect, or low powered analysis and inadequate sample size). Moreover, there is no previously conducted study in Ethiopia.

Therefore, this study aims to improve MHSU among women of reproductive age in Dale and Wonsho districts of the Sidama region, Ethiopia.

The specific objectives are:

**Paper I:** To assess the prevalence and predictors of MHSU among women of reproductive age in Dale and Wonsho districts of the Sidama region, Ethiopia.

**Paper II:** To explore the perceptions, barriers, and facilitators of MHSU among mothers and service providers in Dale and Wonsho districts of the Sidama region, Ethiopia.

**Paper III:** To assess the effect of HEI on the knowledge of mothers regarding ODS and BPCR practice among women of reproductive age in Dale and Wonsho districts of the Sidama region, Ethiopia.

**Paper IV:** To evaluate the effect of HEI on MHSU among women of reproductive age in Dale and Wonsho districts of the Sidama region, Ethiopia.

**Research hypothesis**

• **H_o_:** The CLHEI has no a significant effect on the knowledge of mothers regarding ODS, BPCR practice and MHSU.

• **H_A_:** The CLHEI has a significant effect on the knowledge of mothers regarding ODS, BPCR practice and MHSU.

## Significance of the study

The current study is designed to empower the women with knowledge and skill for improved utilization of existing MHS for the progress of women and newborn health outcomes. These tactics will benefit the governmental organizations, other development allies, and non-governmental organizations to relieve the severe scarcity of human resources in developing countries including Ethiopia. However, the participatory community level and HEI studies for MHC improvement in African regions are scarce. Moreover, there is no previously conducted study in Ethiopia.

Therefore, the epidemiological evidence is a prerequisite to developing appropriate intervention strategies or to filling the existing knowledge gaps. The results from this study will be helpful to inform program managers, policymakers, and implementers in designing effective intervention strategies to improve MHSU and achieve SDGs. The results will also be useful to encourage evidence-based decision-making to address the problems Ethiopian women face throughout the continuum of care. Furthermore, this study will be generated results that can inform the maternal health champions by offering predictors, barriers, and facilitators working in the context of the Sidama region. It also provides evidence for the effective utilization of resources by coming up with an evidence-based recommendation. Moreover, it acts as a source of information for those interested to conduct further studies elsewhere.

**Methods and materials**

**Study area**

This study will be conducted in 2 districts (Dale and Wonsho) of the Sidama region, Ethiopia. Dale district has an overall population of 268, 839 with an estimated total of 53, 768 households (HHs) and comprises an area of 30,212 km2. Of these, 24% were WRA. The district has consisted of two urban and thirty-six rural *kebeles* (the least administrative unit of Ethiopian structures). The Wonsho district has an overall population of 129, 730 with an estimated total of 21, 857 HHs and comprises an area of 14,528 km2. The district has consisted of 1 urban and seventeen rural *kebeles.* Farming or agriculture is the major share of income-producing activity in both districts. The main crops grown in both districts are coffee, *enset* (false banana), khat, barely, khat, haricot beans, corns, sweet potatoes, and local varieties of cabbage. The Dale woreda has consisted of 10 health centers and 33 health posts, whereas Wonsho woreda has 5 health centers and 17 health posts. This site was chosen based on concern of the existing structure to provide a sampling frame, experienced data collectors and supervisors, access to transportation, and a good geographical location.

**Study design**

A community-based cross-sectional study, phenomenological qualitative study, and parallel-group Cluster Randomized Controlled Trial (CRCT) will be used to address the objectives of the current study. The study is a 2 arm CRCT, with every cluster being *kebeles* located in the Dale and Wonsho districts of Sidama region, Ethiopia. The hallmark of this design is each cluster remains in the arm it was randomly assigned to during the course of the whole study period. Thus, all study subjects are randomly assigned into a group and all the study subjects in the assigned group obtain or do not obtain an intervention.

**Study subject**

Study subject will be all WRA, service users and providers, all pregnant women residing in Dale and Wonsho districts of the Sidama region, Ethiopia.

**The inclusion and exclusion criteria**

All systematically selected WRA who gave live birth in the last 12 months, all purposely selected service providers and users, all pregnant mothers residing in the selected *kebeles* for at least for 6 months and have no plan to change residence during the implementation of an intervention, no psychiatric problems, capable of providing written informed consent, and voluntary to be visited by WDT facilitators, data collectors, and supervisors will be included for this study. Women who have a severe illness during the data collection period will be excluded from this study due to unable to communicate. Also, study subject who have a psychiatric disorder will be excluded due to that could affect participation and consent procedure. Similarly, women who experienced stillbirth and infant deaths will be excluded because the questions from this study are expected to re-memorize women the unfavorable pregnancy outcome they had faced. This may seriously affect maternal participation and genuine response. Midwives who have been serving in the maternal and child health case team for less than two years will be excluded. Due to this reason, these midwives have less experience and interaction with pregnant women and couldn’t provide in-depth information on perceptions, barriers, and facilitators of MHSU. Moreover, all WRA included in the quantitative study will be excluded because they have information during the quantitative study that may introduce information bias.

**Sample size calculation**

The adequate sample size for all objectives is calculated using openepi version 3 software. The sample size needed to estimate the utilization of MHS is calculated assuming the anticipated utilization of ANC (74%), births attended in an HF (48%), and PNC (34%) according to the report of 2019 Mini EDHS[[29](#_ENREF_29)], the margin of error of 5%, 95% confidence interval, 1.31 design effect. Based on the above information, the estimated sample size is 392 (ANC), 508 (births attended in a health facility), and 457 (PNC). The sample size needed for identifying the predictors associated with MHSU is calculated considering variables significantly associated with MHSU based on previous studies done in Northern Ethiopia [7], in rural Haramaya district, Eastern Ethiopia [10], and in Northwest Ethiopia [11]. For all calculations, power was set at 80%, level of confidence at 5%, and the ratio of unexposed-to-exposed were assumed to be 1. Hence, the sample size of 1,104 obtained from the second objective will be utilized due to it is the maximum sample size assessed and would be adequate to assess all objectives of this study. The minimum required sample size for the qualitative study will be determined according to the recommendation of Morse and Creswell for Phenomenological studies (20 in-depth interviews, 9 focus group discussions, and 20 key informant interviews) [[30](#_ENREF_30)]. The minimum required sample size for the third and fourth objectives is calculated based on the following considerations. Due to the lack of the previous cRCT on the same topic, assumptions on the proportion of women with knowledge on ODS in the control and intervention arms were taken from a previous quasi-experimental study. The proportion before the intervention was taken as the proportion in the control arm and the proportion after the intervention as the proportion in the intervention arm. Accordingly, P1 = 45.7% (proportion of women’s knowledge regarding ODS during pregnancy) in the control group, P2= 62.9% (proportion of women’s knowledge regarding ODS during pregnancy in the intervention arm) [[14](#_ENREF_14)], 95% CI, and 80% power. According to the above considerations, the estimated effective sample size for individual-based randomization is 286. For this objective, the sample size is adjusted to non-response rate (NRR) by dividing the initial sample size by the anticipated response rate. In case of knowledge regarding ODS during pregnancy, the adjusted sample size to NRR is 286/1 = 286.The minimum required number of clusters is computed by multiplying the effective sample size and ICC factors for both groups [28]. We received the typical value of ICC factor of 0.01 from range values (0.01 to 0.05) based on the existing recommendation [26]. Consequently, the minimum needed cluster number is 329*0.01 = 3.29 for both groups. Nevertheless, to maintain the sufficiency of the cluster and to have adequate power [26, 28], 12 clusters (*12 kebeles*) will be included in this study. To account for the effect of the cluster, the effective sample size is multiplied by a VIF of 1.26. The VIF is calculated with the assumption of an equal cluster size of 27 study subjects from 12 clusters (*kebeles*). Using ICC value of 0.01, (VIF = 1+ [(n-1) ICC]), where ‘n’ is the average cluster size [28]. Thus, the final estimated sample size is 360 (180 in the intervention arm and 180 in the control arm). However, the sample size calculated for the fourth objective which is designed to evaluate the effect of the intervention on MHSU is 1,126 (i.e., larger). Hence, the sample size of 1,126 obtained from the fourth objective will be utilized for this objective as well as it would be maximum or representatives of the sample for both objectives.

**Methods of selection of study respondents**

A multi-stage, purposive and stratified sampling technique will be used to select the study participants.

**Blinding**

Because of the nature of the study intervention neither research team members nor study respondents can be masked.

**Data collection and quality control**

The study tools will be initially prepared in English, translated to Sidamic language, and reconverted back to English to retain its originality and consistency. The assessment will be conducted to evaluate the inconsistency among the 2 versions of the tools. It will be pre-tested on the 5% of sample size in kebeles that are not involved in the actual research area. Then, any inconsistency between the 2 versions will be amended accordingly. The training regarding tools will be provided for the data collectors and supervisors by the principal investigator for two days. During the training, attention will be given to the significance of the study, data collection procedure, objective, methods, and ethical issues. The quantitative and qualitative data collection will be conducted by employees of Hawassa University Health and Demographic Surveillance System (HDSS) and masters of public health experts. Two public health experts with RH will be carefully supervised the data collection method. The consistent checkup for incompleteness and non-consistency of the data will be made on a daily basis. Double data entry will be carried out. The incomplete, inconsistent, and invalid data will be distinguished correctly to acquire a high quality of data before, during, and after data entry. The corrections will be made based on the original data.

**Intervention procedure**

WDA volunteers will be recruited by using the following selection criteria as able to read and write the Sidama language and are willing to do the intervention. Following recruitment, intensive training will be given for 1 week on the topics. One health education session consists of key messages on normal pregnancy, danger signs and complications during pregnancy, delivery, and the postpartum period, the practice of birth preparedness as well as complication readiness, and the benefits of MHSU. The other actions executed by WDA facilitators will be the motivating mother and their families for MHSU. One hour will be allocated for each session, from this 20 minutes will be allowed for the lecture which is moralistic in nature and the remaining 40 minutes will be allowed for raising questions and responses. After the session, some of the women will be selected to carry out role play which is basic to demonstrate the important messages and share experiences. This will be done to repeat the information provided to enable them to internalize the key message. The women will be also exposed to posters to reinforce the session message. The session will be conducted 2 times per month for six months. The follow-up will be conducted every month or more repeatedly if any potential problems indicated.

Interventional group (n= 563)

Control group (n= 563)

Included in to study (n = 1,126)

Randomization

Received CLHEI (n= 563)

Loss to follow up (n=)

Loss to follow-up (n=)

Analyzed at 6 months (n=)

Received routine intervention (n= 563)

Analyzed at 6 months (n=)

**Figure 1:** Flow Diagram for randomization of study participants in to intervention and control groups in Dale and Wonsho districts of Sidama region, Ethiopia.

**The study variables**

| Outcome variables | Definition |
| --- | --- |
| MHSU | Utilization of maternal health care measured using the yes or no questions at the baseline and after 6 months of intervention. |
| Knowledge of obstetrics danger signs | Will be measured by using the 30 knowledge assessment questions. |
| Birth preparedness and complication readiness practice | Will be assessed using the 5 practice assessment questions. |
| Perceptions, barriers, and facilitators | Perceptions, barriers, and facilitators of MHSU that affect the utilization of MHS will be explored qualitatively. |
| Exposure variable | Definition |
| Health education | **Intervention group:** will receive routine plus pre-recorded audio-based HEI package for six months until date of delivery.  **Comparator group:** will receive the routine health education package for six months until the date of delivery as per the Ethiopian guidelines. |
| Predictors | Predictors that predict the utilization of MHS. It will be measured retrospectively. Socio-economic and demographic, obstetric characteristics, health facility and socio-cultural related predictors. |
| Covariates | Place of residence (urban/rural), educational status of women and her spouse, wealth index, maternal age, occupational status of women and her spouse |

**Data source**

The data sources are women of reproductive age, service users and providers.

**The measurements**

**ANC utilization** is measured as a proportion of women who utilized ANC from skilled providers (HEWs, midwives, nurses, health officers, and doctors) at least once during the last pregnancy using a woman's self-report. However, different studies considered different figures of ANC visits being optimum. For instance, some researchers had fixed 2 ANC visits to be adequate. The WHO suggests at least 8 ANC visits throughout pregnancy while the first visit will be conducted before 16 weeks or within the first trimester. Therefore, standardization of the numbers of ANC use is required to assist the comparison of diverse or different studies. Accordingly, we considered utilization of ANC as a woman who has at least one ANC follow-up which is similar to EDHS. Besides, the time must be within the first trimester or before 16 weeks of gestation. Moreover, women must consume more than 90 iron-folic acids (IFA) tablets or syrup, receive at least one dose of tetanus toxoid injection, have a laboratory test result of syphilis, and blood group.

**Institutional delivery utilization** is measured as the proportion of women who utilized institutions (governmental health centers or hospitals, and/or private hospitals or clinics) for delivery in their last pregnancy using a woman’s self-report.

**PNC utilization** is measured as the proportion of women who utilized a PNC visit within 42 days after giving birth at least once using a woman’s self-report.

**Maternal knowledge regarding ODS** will be measured using the 30 questions during three phases namely antepartum (9 questions), intrapartum (12 questions), and postpartum (9 questions). The correct answers are assigned a score of 1, while incorrect answers are assigned a score of 0. Lastly, the total knowledge scores range from 0 to 30. The study respondents who spontaneously mention at least 3 ODS during each phase are classified as having “good knowledge” and those who are not able to spontaneously mention 2 or fewer ODS have “poor knowledge”.

**Spontaneous knowledge** is defined as the knowledge of study participants who can name or call an ODS without being read the name of that sign by data collectors. Merely true ODS spontaneously mentioned by study participants will be recorded during the interview.

**BPCR practice** will be measured using 5 components of the question as to whether or not the woman planned for her most recent pregnancy such as identified a closer proper HF for childbirth, founded and communicate an SBA, saved money, a material resource for childbirth, and other associated costs, prepared or arranged transportation to a proper HF in case of childbirth and obstetric emergency and identified and fixed the compatible blood group givers in case of blood requirements. If a woman prearranged at least three components out of 5, she will be considered as having “well prepared” and those who are prepared 2 or fewer considered “poorly prepared”.

**Bias**

To minimize the biases the maximum efforts will be taken by careful selection of subjects that represent the source population, maximizing response and follow-up rates, standardization of data collection process, provision of training for the data collectors and supervisors, and randomization. To decrease loss to follow-up, study participants will be reminded by WDA facilitators 2 days before the CLHEI date.

**Statistical data analysis**

Data will be entered into Epidata version 3.1 and exported to the Statistical Package for Social Science (SPSS) version 25 and Stata version 14 for further processing and analysis. During the analysis, all quantitative variables will be handled by recoding, calculations, and categorizations before carrying out the main analysis. Descriptive analyses will be carried out to get descriptive measures for the important variables of interest. The descriptive statistic techniques will be utilized for the data organization and presentation. All continuous data will be checked for the normal distribution. If approximately normal distributed the mean and SD will be reported. The missing data will be handled by using multiple imputation methods. The Principal Component Analysis (PCA) will be carried out for the computation of the wealth index. The effect of clustering will be checked by using an Intra-Cluster Correlation Coefficient (ICC) value. The multilevel analysis will be considered if the calculated value of ICC is greater than 5%. Before conducting a multilevel analysis, we will decide whether or not the multilevel model is required by doing a random intercept model. This model offers data regarding ICC which is utilized to determine whether a multilevel model is required or not. If the chi-square test is significant or the ICC value is greater than 5%, the multilevel analysis model is mandatory to consider. Both bi-variable and multivariable analyses using generalized mixed models with random effects will be carried out. The Generalized Estimated Equations (GEE) or multilevel regression models will be utilized to adjust for between and within-cluster variation for adjusted analysis. Those variables with p-values < 0.25 on the bi-variable analysis model will be included in a multivariable regression model to find out predictors independently associated with MHSU adjusting for other variables in the model. The candidate variables will be entered into the multivariable regression model using the ENTER regression method. The basic assumptions of generalized mixed models will be tested before proceeding with the analysis. The stratified and multivariable analyses will be used to control for the potential confounding variables. Effect modification will be examined using stratification and entering effect modifiers into the multivariable analysis model one at a moment. Multicollinearity among the independent variables will be also evaluated using a multiple linear regression model. The variance inflation factor (VIF) < 10 and tolerance statistics > 0.1 will be used to declare the absence of multicollinearity. The presence and strength of a statistically significant association between MHSU and the independent variables will be assessed using adjusted odds ratios (AORs) with a 95% CI. A statistically significant association between the variables of interest will be confirmed when the 95% CI of the AORs did not contain 1. All analyses of the study will be carried out using a 95% CI or 5% level of significance. Though, a statistical significance level will be adjusted to account for the effect of multiple comparisons problem using the Bonferroni corrections methods. The adjusted level of significance is computed by dividing the preset level of significance by the number of statistical tests conducted (outcome variables). In our case, the adjusted level of significance is 0.05/3 = 0.017. Thus, a statistically significant association will be declared when p-value less than 0.017. After the finish of the qualitative data collection, the tape-recorded data will be transcribed verbatim of the local language and translated to English. The coding and analysis of the qualitative data will be done using the thematic content analysis technique by Atlas-Ti software and presented in narratives. A paired t-test will be utilized to measure the mean change from baseline for both groups (intervention and control) groups. Then, an independent t-test will be utilized to compare the mean change of women’s knowledge regarding ODS and BPCR practice scores and MHSU for interventional groups against the mean change of the control group as effect size. To assess the effect of HEI on maternal knowledge regarding ODS and BPCR practice scores and MHSU, an intention-to-treat analysis will be done to compare outcomes between the two groups.

**Auditing**

This study is open for auditing via any concerned body or organization such as governmental and non-governmental.

**The study protocol modification**

Timely communication will be conducted about the study protocol amendment with the concerned body like IRB and project Sponsors/funders.

**Dissemination of the study findings**

The study findings will be officially presented to the Hawassa University School of Public Health. The summary of the main findings will be delivered to the district health office and the Sidama region health bureau. The findings will also be distributed to vital stakeholders in maternal health policy, comprising the WHO. Moreover, the findings of the study will be published in peer-reviewed international journals and discussed at national and international seminars and conferences.

**CONSORT 2010 statement** **and STROBE guidelines**

The primary outcomes of this study will be reported based on CONSORT 2010 statement: extension to cluster randomized trials guideline. The secondary outcomes were prepared based on the Strengthening the Reporting of Observational Studies in Epidemiology (STROBE) guideline.

**Ethical considerations**

The ethical approval of this protocol will be obtained from the Institutional Review Board (IRB) of Hawassa University. The aim of the study and methods of data collection, privacy, voluntary participation, potential benefits, and harms will be described to the study participants before signing informed written consent. The written informed consent will be received from all study participants who meet inclusion criteria before enrollment. All data collection methods and intervention techniques will be carried out in confidentiality. The maximum efforts will be exerted to register the protocol in the international community trials registry.

**Schedule and budget:** This study will be conducted from October to august 2023; the total cost of the project will be **421,600 ETB (7,026.67 EURO)** and funded by Hawassa University only **60,000 ETB (1053 EURO)**.

# EXPECTED OUTCOMES

We expect the current study will provide four documents that can be disseminated by published articles reports and conference presentations. The first paper will deal with utilization and predictors of maternal health service among women of reproductive age. The second paper provides in-depth evidence on perceptions, barriers, and facilitators of MHSU among service providers and users. The third paper will provide epidemiological evidence about the effect of HEI on maternal knowledge towards ODS and BPCR practice among women of reproductive age using WDT facilitators. The last paper is expected to provide evidence of the effect of an HEI on the MHSU among women of reproductive age in Dale and Wonsho districts of Sidama region, Ethiopia. This large-scale study comparing community-level HEI with routine intervention for improving maternal health will be expected to provide key evidence for policymakers and planners concerning the best design of MHSU to improve maternal health.

**Reference**

1. World Health Organization (WHO), Maternal mortality. Available from https://[www.who.int/news-room/fact-sheets/detail/maternal-mortality](http://www.who.int/news-room/fact-sheets/detail/maternal-mortality)

2. Borde MT, Loha E, Johansson KA, Lindtjorn B. Utilisation of health services fails to meet the needs of pregnancy-related illnesses in rural southern Ethiopia: A prospective cohort study. PLoS One. 2019 Dec 4;14(12):e0215195. doi: 10.1371/journal.pone.0215195.

3. Central Statistical Agency (CSA). “[Ethiopia] and ICF,” Ethiopia Minin Demographic and Health Survey 2019: Key Indicators Report: Addis Ababa, Ethiopia, and Rockville, CSA and ICF, Maryland, USA.

4. Borde MT, Loha E, Johansson KA, Lindtjørn B. Financial risk of seeking maternal and neonatal healthcare in southern Ethiopia: a cohort study of rural households. Int J Equity Health. 2020 May 18;19(1):69. doi: 10.1186/s12939-020-01183-7.

5. Borde MT, Loha E, Lindtjørn B. Incidence of postpartum and neonatal illnesses and utilization of healthcare services in rural communities in southern Ethiopia: A prospective cohort study. PLoS One. 2020 Aug 27;15(8):e0237852. doi: 10.1371/journal.pone.0237852.

6. Abosse Z, Woldie M, Ololo S. Factors influencing antenatal care service utilization in hadiya zone. Ethiop J Health Sci. 2010 Jul;20(2):75-82. doi: 10.4314/ejhs.v20i2.69432.

7. Mamuye SA. Magnitude and Determinants of Postnatal Care Service Utilization Among Women Who Gave Birth in the Last 12 Months in Northern Ethiopia: A Cross-Sectional Study. Int J Womens Health. 2020 Nov 13;12:1057-1064. doi: 10.2147/IJWH.S269704.

8. Geleto A, Chojenta C, Taddele T, Loxton D. Association between maternal mortality and caesarean section in Ethiopia: a national cross-sectional study. BMC Pregnancy Childbirth. 2020 Oct 6;20(1):588. doi: 10.1186/s12884-020-03276-1.

9. Berelie Y, Yeshiwas D, Yismaw L, Alene M. Determinants of institutional delivery service utilization in Ethiopia: a population based cross sectional study. BMC Public Health. 2020 Jul 8;20(1):1077. doi: 10.1186/s12889-020-09125-2.

10. Kifle D, Azale T, Gelaw YA, Melsew YA. Maternal health care service seeking behaviors and associated factors among women in rural Haramaya District, Eastern Ethiopia: a triangulated community-based cross-sectional study. Reprod Health. 2017 Jan 13;14(1):6.

11. Ayalew TW, Nigatu AM. Focused antenatal care utilization and associated factors in Debre Tabor Town, northwest Ethiopia, 2017. BMC Res Notes. 2018 Nov 16;11(1):819. doi: 10.1186/s13104-018-3928-y.

12. Aktaç S, Sabuncular G, Kargin D, Gunes FE. Evaluation of Nutrition Knowledge of Pregnant Women before and after Nutrition Education according to Sociodemographic Characteristics. Ecol Food Nutr. 2018 Nov-Dec;57(6):441-455. doi: 10.1080/03670244.2018.1544561. Epub 2018 Nov 13.

13. Masoi TJ, Kibusi SM. Improving pregnant women's knowledge on danger signs and birth preparedness practices using an interactive mobile messaging alert system in Dodoma region, Tanzania: a controlled quasi experimental study. Reprod Health. 2019 Dec 12;16(1):177. doi: 10.1186/s12978-019-0838-y.

14. Fetohy EM. Impact of a simple health education program about antenatal care on knowledge, attitudes, subjective norms and intention of pregnant women. J Egypt Public Health Assoc. 2004; 79(3-4):283-310.

15. Kamau M, Mirie W, Kimani S, Mugoya I. Effect of community based health education on knowledge and attitude towards iron and folic acid supplementation among pregnant women in Kiambu County, Kenya: A quasi experimental study. PLoS One. 2019 Nov 25;14 (11):e0224361. doi: 10.1371/journal.pone.0224361.

16. Vural F, Vural B. The effect of prenatal and postnatal education on exclusive breastfeeding rates. Minerva Pediatr. 2017 Feb;69(1):22-29. doi: 10.23736/S0026-4946.16.04183-9.

17. Caine VA, Smith M, Beasley Y, Brown HL. The impact of prenatal education on behavioral changes toward breast feeding and smoking cessation in a healthy start population. J Natl Med Assoc. 2012 May-Jun;104(5-6):258-64. doi: 10.1016/s0027-9684(15)30159-0.

18. Umar NJ, Afolayan JL, Emmanuel EA, Rejuaro FM, Onasoga OA, et al. (2017) Impact of Health Education on Knowledge and Access to Delivery Care Services by Women among Edu Local Government Area, Nigeria. J Community Med Health Educ 7: 510. doi:10.4172/2161-0711.1000510

19. Lassi ZS, Kedzior SG, Bhutta ZA. Community-based maternal and newborn educational care packages for improving neonatal health and survival in low- and middle-income countries. Cochrane Database Syst Rev. 2019 Nov 5;2019(11):CD007647. doi: 10.1002/14651858.

20. UM Ango, MO Oche, IS Abubakar, KJ Awosan, Kaoje AU, MO Raji. Effect of health education intervention on knowledge and utilization of health facility delivery services by pregnant women in Sokoto State, Nigeria. International Journal of Contemporary Medical Research 2018;5(6):F4-F9.

21. Shimpuku Y, Madeni FE, Horiuchi S, Kubota K, Leshabari SC. A family-oriented antenatal education program to improve birth preparedness and maternal-infant birth outcomes: A cross sectional evaluation study. Reprod Health. 2019 Jul 16;16(1):107. doi: 10.1186/s12978-019-0776-8.

22. Jibril UN, Saleh GN, Kayode OS, Morisola RF, Umar A, et al. Impact of Health Education Intervention on Knowledge and Utilization of Postnatal Care Services among Women in Edu Local Government of Kwara State, Nigeria doi: 10.4103/2278-960X.194496

23. Izudi J, Akwang DG, McCoy SI, Bajunirwe F, Kadengye DT. Effect of health education on birth preparedness and complication readiness on the use of maternal health services: A propensity score-matched analysis. Midwifery. 2019 Nov;78:78-84. doi: 10.1016/j.midw.2019.08.003.

24. OKAFOR, Orji Urenna; YEWANDE, Ademuyiwa Iyabo. Effect of antenatal education on knowledge and utilization of facility-based delivery services among pregnant women in two health institutions in Alimosho, Lagos state. International Journal of Research in Medical Sciences, [S.l.], v. 8, n. 10, p. 3457-3462, sep. 2020. ISSN 2320-6012.

25. Soriano-Martín PJ, Tejedor-Tornero A, Castro-Sánchez E. The effect of prenatal education classes on the birth expectations of Spanish women. Midwifery. 2018 May;60:41-47. doi: 10.1016/j.midw.2018.02.002.

26. Bolam A, Manandhar DS, Shrestha P, Ellis M, Costello AM. The effects of postnatal health education for mothers on infant care and family planning practices in Nepal: a randomised controlled trial. BMJ. 1998 Mar 14;316(7134):805-11. doi: 10.1136/bmj.316.7134.805.

27. Belizán JM, Barros F, Langer A, Farnot U, Victora C, Villar J. Impact of health education during pregnancy on behavior and utilization of health resources. Latin American Network for Perinatal and Reproductive Research. Am J Obstet Gynecol. 1995 Sep;173(3 Pt 1):894-9. doi: 10.1016/0002-9378(95)90362-3.

28. MacArthur C, Jolly K, Ingram L, Freemantle N, Dennis CL, Hamburger R, Brown J, Chambers J, Khan K. Antenatal peer support workers and initiation of breast feeding: cluster randomised controlled trial. BMJ. 2009 Jan 30;338:b131. doi: 10.1136/bmj.b131.

29. Central Statistical Agency (CSA) [Ethiopia] and ICF. Mini Ethiopia Demographic and Health Survey 2019: Key Indicators Report. Addis Ababa, Ethiopia, and Rockville, Maryland, USA. CSA and ICF. 2019.

30. Creswell J. W, Qualitative Inquiry & Research Design: Choosing Among Five Traditions. Thousand Oaks: CA. Sag Publications, Inc. 1998.
