# Supplementary material for: Effect of community health education on mothers’ knowledge of obstetric danger signs and birth preparedness and complication readiness practices in southern Ethiopia: A cluster randomized controlled trial
Source: PLoS One. 2024 Nov 27;19(11):e0312267. doi: 10.1371/journal.pone.0312267 (PMC11602057; doi:10.1371/journal.pone.0312267)
Supplement: Attachment — Submitted filename: Reviewers Dr. Amanuel Yoseph.pdf [file pone.0312267.s007.pdf]

PONE-D-23-40070R1

Effect of community health education on mothers' knowledge of obstetric danger signs and birth preparedness and complication readiness practices in southern Ethiopia: A cluster randomized controlled trial

PLOS ONE

Reviewer 1

1. There are a lot Keywords, usually require about 3-8 keywords but 9 are a lot.
2. The title is so long too. I suggest: "community health education on mothers' knowledge with obstetric risk signs, birth preparedness and complication readiness practices in southern Ethiopia: A cluster randomized controlled trial"
3. Focus on the variables identified in the title so as not to make the writing so extensive.

Reviewer 2

Comments to authors:

Thank you for the opportunity to review this manuscript. Their results highlight the effectiveness of community-based interventions to increase women's knowledge about pregnancy complications and best practices which is necessary to reduce maternal and infant mortality rates worldwide. The manuscript would benefit from some editing and summarizing some sections as it is very long and hard to follow at times. Please see comments below.

Introduction
1. The introduction could be significantly shortened, and some of the information used more in the discussion section. I would suggest the following structure;
a. Paragraph describing ODS including some prevalence data
b. Paragraph addressing the women's knowledge gap of ODS
c. Paragraph describing interventions and government efforts
d. Paragraph describing the lack of research and objectives of this manuscript
2. It would be good for the authors to add some data on prevalence of ODS and maternal mortality particularly in Ethiopia.

Methods
3. Adding a flow chart or diagram depicting the participant's selection and randomization process would be helpful to follow the study design, population and randomization paragraphs a little better.
4. Study variables: I suggest the authors only focus on variables that are relevant for this manuscript.
5. I suggest moving the description of the intervention to supplementary material and including only a brief summary of it in the manuscript. I also think it should be placed before talking about the outcomes.
6. Page 14 paragraph 2 talks about data collection and then goes back to randomization, this is a little confusing. I would suggest authors to mention everything that has to do with randomization in the appropriate paragraph and not coming both.

7. While the authors offer very detailed information and justifications for their model selection, I'm a little confused if their multilevel models were linear or Poisson models. It is not the standard to estimate ICC from a Poisson model and usually variance portioning is used. If fitting a logistic model then MOR is what is usually estimated as opposed to ICC If authors estimated ICC from non-linear models I would suggest that they add the formula of how they did it to the manuscript.
8. Why did authors decide the cutoff of $p < 0.25$ to be included in multivariate models
9. What was the reasoning behind the effect modification tests? Which variables were tested? I would suggest authors to elaborate more on this.
10. In general, authors provide many statistical terms and details to the methods section that makes it hard to follow. I would suggest authors simplify it and add only the important information readers would need in order to replicate this analysis. The section is very hard to follow and all the justification and details can be distracting.

Results
11. On the description of table 1, it would be important to highlight the differences between groups, given that this is an RCT and Table 1 should be used to assess balance between groups. The statistically significant differences for Mass Media access and wealth index are important and could be associated with the outcome as well so should be highlighted.
12. Similar to previous comment, it is important to highlight significant differences between groups, previous history of neonatal death and ODS during last pregnancy could be associated with the outcomes as well and should be highlighted as potential confounders.
13. I find tables 3 and 4 a little confusing. I would separate the N(%) of good and poor knowledge into a separate table and just leave the model results. Also, it is not very clear which models the authors are showing. In the methods they refer to a sequence of four models and they only show results for 2, clarifying that would be very helpful.

Discussion
14. While the information provided is good and authors do a good job of comparing their findings to other literature, the limitations section is very long and disorganized. I would strongly advise to summarize better the limitations with regards to clustered RCTs and then they can talk about limitations of analysis or other types instead of going back an forth which makes it hard to follow.

Reviewer 3

Amanuel Yoseph and co-authors evaluated the effect of community-based health education intervention facilitated by women's groups (women's development team) on mothers' knowledge of SDG and BPCR practice in Sidama region in the southern Ethiopia.

I would like to thank the authors for this excellent manuscript. A problem that affects a high proportion of women in underdeveloped countries is outlined: high maternal morbidity and mortality. But, even more importantly, the authors present evidence on effective strategies to mitigate this important public health problem. Adding to the relevance of the topic is the correct design, the careful handling of ethical aspects, a robust analysis and the writing of the manuscript.
My only suggestion is to review the title, its length seems a bit excessive to me. Additionally, I consider the comment "while another paper is focused on three skilled [...], in the "Study variables" section, unnecessary.

Congratulations, I really enjoyed reading the manuscript

Reviewer 4

The topic is relevant because of the social impact and inequalities that occur in developing countries. It is essential to recognize indicators of potential risks during childbirth in order to make informed decisions and prevent complications that could lead to the death of both mother and baby. However, we have some observations.

Overall, the current version of the article is quite dense and difficult to understand because of long and ambiguous sections. To ensure clarity for the reader, it is essential to reorganize the structure of the paper. In addition, it is substantial in the paper, to balance the section statistical analysis with importance of the impact of the intervention, will allowing the validity of the study to be measured.

The specific comments below recommend reducing the text in certain sections:

Introduction Section: From our perspective, the ideas in this section are long and repetitive. We recommend condensing sentences 2, 3, and 8. Sentence 6 could be rewritten or omitted.

In order to develop an educational intervention that addresses complications and reduces maternal and infant mortality rate, it is crucial to have a solid theoretical and causal framework. The causal framework is a diagram that considers the factors associated with social knowledge and establishes clear theoretical relationships between these factors. By incorporating a causal framework, the intervention can show its function and impact, highlighting the desired variable of change and the desired health outcomes. In addition, the baseline measure allows identifying should identify the variables that should be included in the analysis conceptual and ensure factor similarity between the intervention and non-intervention groups. It also allows for including relevant variables in the statistical analysis. The causal framework will justify the importance and scope of the intervention. For further help, see the following references:

• Rossi, P. H., Lipsey, M. W., & Freeman, H. E. (2004). Evaluation: A Systematic Approach (7th ed.). Thousand Oaks, CA: SAGE Publications. This classic program evaluation text underscores the importance of understanding and documenting causal relationships in intervention programs.

Centers for Disease Control and Prevention (CDC). (2011). Developing an Effective Evaluation Plan. Atlanta, GA: CDC, National Center for Chronic Disease Prevention and Health Promotion. This paper details the importance of causal and logic frameworks in health program evaluation.

• Funnell, S. C., & Rogers, P. J. (2011). Purposeful Program Theory: Effective Use of Theories of Change and Logic Models. San Francisco, CA: Jossey-Bass. This book provides comprehensive guidance on how to develop and use theories of change and logic models in program evaluation.

• Weiss, C. H. (1997). Theory-Based Evaluation: Past, Present, and Future. New Directions for Evaluation, 1997(76), 41-55. This article reviews the history and utility of theory-based evaluations, including the importance of causal frameworks.

• Bamberger, M., Rugh, J., & Mabry, L. (2012). RealWorld Evaluation: Working Under Budget, Time, Data, and Political Constraints (2nd ed.). Thousand Oaks, CA: SAGE Publications. This book addresses the challenges and strategies for evaluation in real-world settings, including justifying interventions through causal frameworks.

Method Section:

As a general comment, this section should be revised and summarized.

It is recommended that the participant recruitment process be described in more detail both in the text and in Figure 1.

I am confused by the way participant recruitment is described. If pregnant women were selected by visiting all households, if so, how was the response rate assessed if some households had a pregnant woman who did not want to be interviewed, or was not home because she had been hospitalized for complications of pregnancy? Other questions: How many households were visited in total? What is the rate of refusal or nonparticipation? During what stage of the recruitment process must women provide consent to take part? What was the community consent process? Given that the intervention was part of a major project, at what point in the study process were the groups randomized?

How do you measure the equivalence of the two groups in personal, social, and health service variables that theoretically take part in knowledge?

In the sample size calculation section, if a household census was conducted and pregnant women were identified, the most appropriate thing to do is to identify the statistical power or precision got when identifying knowledge in the total number of women who took part in the study.

On the other hand, what were the criteria for generating the clusters?

It would be appropriate to summarize this section as well.

After randomization: Are the factors that determine knowledge the same in both groups (intervened and non-intervened)? How do you check if randomization worked in creating kebeles?

In the variables section of the study, when it is noted that "The respondents in the study spontaneously mentioned three questions... spontaneous knowledge was defined... they could spontaneously name two or more..." Question: During the validation of the questionnaire and in particular of the knowledge questions, was the potential information bias that could influence the results quantified?, and in this context, did they measure its potential impact on the misclassification of knowledge in the statistical analysis?

Regarding the measurement of conceptual constructs such as "knowledge" during the analysis of the information, does the questions have the same value to measure the dimensions of the Knowledge construct?

In the last sentence of the same section, they show that this study is part of another larger project with another aim. It is not clear if it affected the creation of representativeness of women in the community or the recruitment and randomization process. Can you clarify?

IES process section. How did the topics offered to improve knowledge used in the intervention emerge? What competencies should participants have at the end of the educational training? Were assessments conducted before the intervention? What were the assessment results at the beginning and during the training process? If so, is there a parameter at the beginning of the intervention to measure the difference in knowledge between the intervention and non-intervention groups? If so, were these controlled for in the statistical analysis? Please comment.

Regarding the sections on data collection procedures, data techniques, and ethical statements, please reduce the length of these sections and rewrite them.

Results section. The statistical analysis should begin by evaluating the difference in the knowledge indicators that allow the initial counterfactual to be evaluated and induce the final counterfactual. It is important, measure the change before and after the intervention in both groups. A knowledge index be developed to meet the stated aim.

The tables are extensive. Please include the results of the important variables.

Explain the statistical differences that allow the identification of variables used in the adjusted statistical model.

Tables 3 and 4, please include at the bottom of the table the variables used to adjust the final model, and show the most essential findings that allow the subsequent discussion of the results.

Create a bar graph showing the percentage of increase in knowledge caused by the intervention that is the objective of the study.

In the Discussion section, it is recommended that the authors include the percentage of change in knowledge about the SDG and BPCR practices got at the end of the intervention.

An important aspect of the discussion of the study findings is the statistical analysis, which includes both intention to treat and final analysis.

The authors refer to an increased likelihood of women performing BPCR after the intervention, but do not specify which specific outcomes they are referring to.

The discussion mentions potential cost and material savings, but it is unclear whether this was one of the study's goals.

In conclusion, the paper cannot be published as it is, observations and comments are necessary.
