# Supplementary material for: Effect of community health education on mothers’ knowledge of obstetric danger signs and birth preparedness and complication readiness practices in southern Ethiopia: A cluster randomized controlled trial
Source: PLoS One. 2024 Nov 27;19(11):e0312267. doi: 10.1371/journal.pone.0312267 (PMC11602057; doi:10.1371/journal.pone.0312267)
Supplement: Attachment — Submitted filename: Response to Reviewers.docx [file pone.0312267.s006.docx]

***Point-by-point responses to reviewers’ and editor’s comments***

**Academic editor**

**Comment 1**:  I currently have four reviews for your paper PONE-D-23-40070. The reviewers, who have important expertise in areas your paper covers, including health interventions and methodology, overall found the manuscript of potential interest and useful findings; however they raised important topics that need be addressed by author in the resubmission. Taking into account the reviewers' comments will improve the manuscript for acceptance. The changes should be observed mainly in terms of synthesizing the introduction, strengthening the methodology and strengthening the discussion and conclusions.

**Authors’ response**: Thank you for your kind remark. We duly considered all comments and have revised the manuscript accordingly.

**Comment 2:** My suggestions: follow the criteria for publications in PLOS ONE, reduce the number of keywords, and check consistency between all sections of the manuscript.

**Authors’ response:** Thank you a lot for this valuable comment. We have accepted the comment and made the required revision. The keywords have been edited to be consistent with PLOS ONE publications criteria as per your suggestion. Besides, we have checked the consistency of all section of our manuscript.

**Comment 3:** If you would like to make changes to your financial disclosure, please include your updated statement in your cover letter. Guidelines for resubmitting your figure files are available below the reviewer comments at the end of this letter.

**Authors’ response**: Thank you for this important notice. However, we didn’t change our financial disclosure because we have correctly stated it in the online submission system. Besides, we have already followed the figures files preparation style of PLOS ONE journal.

**Comment 4:** If applicable, we recommend that you deposit your laboratory protocols in [protocols.io](http://protocols.io/) to enhance the reproducibility of your results. Protocols.io assigns your protocol its own identifier (DOI) so that it can be cited independently in the future.

**Authors’ response**: Thank you for this recommendation. This study hasn’t laboratory protocol but has a study protocol. We have already provided the study protocol as a supplementary material.

**Reviewer 1**

**Comment 1:** There are a lot Keywords, usually require about 3-8 keywords but 9 are a lot.

**Authors’ response:** Thank you very much for your vital comments. As per your comment and comment 2 from the editor, we have revised the keywords to align with journal requirement.

**Comment 2:** The title is so long too. I suggest: "community health education on mothers' knowledge with obstetric risk signs, birth preparedness and complication readiness practices in southern Ethiopia: A cluster randomized controlled trial"

**Authors’ response:** Thank you, too, for this comment. We have accepted your comment and made the required revisions as per your comment. The slight deviation in the title from your suggested title is meant only for grammatical correctness and clarity.

**Comment 3:** Focus on the variables identified in the title so as not to make the writing so extensive.

**Authors’ response:** Thank you for pointing this out. Yes, what you pointed out is a possibility. However, the clear definition of the exposures and outcomes are very important to provide comprehensive information for readers on how variables are measured. Moreover, the variables measurement is not accurately and consistently provided across the medical literature that precludes the replication of measurement and comparison of findings by scholar in different setting and time. Thus, we provided the clear definition separately to each variable to facilitate replication and comparison by different researchers.

**Journal requirements**

**Requirement 1:** Please ensure that your manuscript meets PLOS ONE's style requirements, including those for file naming.

**Authors’ response:** We have followed PLOS ONE’s style requirements, including file naming conventions, in preparing the manuscript.

**Requirement 2:** Note from Emily Chenette, Editor in Chief of PLOS ONE, and Iain Hrynaszkiewicz, Director of Open Research Solutions at PLOS: Did you know that depositing data in a repository is associated with up to a 25% citation advantage (<https://doi.org/10.1371/journal.pone.0230416>)? If you’ve not already done so, consider depositing your raw data in a repository to ensure your work is read, appreciated and cited by the largest possible audience. You’ll also earn an Accessible Data icon on your published paper if you deposit your data in any participating repository (<https://plos.org/open-science/open-data/#accessible-data>).

**Authors’ response:** Thank you very much for this vital information. Now we have provided the full data set on repository in which the manuscript is prepared to take advantage of citation as per your suggestion.

**Requirement 3:** Thank you for stating the following financial disclosure: 
 [Hawassa University and Sidama region president office].  Please state what role the funders took in the study.  If the funders had no role, please state: "The funders had no role in study design, data collection and analysis, decision to publish, or preparation of the manuscript."  If this statement is not correct you must amend it as needed.

Please respond by return e-mail so that we can amend your financial disclosure and competing interests on your behalf.

**Authors’ response:** As we also responded to the above comment (comment 3), the financial disclosure statement has been correctly stated as per your suggestion during first online submission in the system. All the required details are provided.
