# Supplementary material for: Effect of community health education on mothers’ knowledge of obstetric danger signs and birth preparedness and complication readiness practices in southern Ethiopia: A cluster randomized controlled trial
Source: PLoS One. 2024 Nov 27;19(11):e0312267. doi: 10.1371/journal.pone.0312267 (PMC11602057; doi:10.1371/journal.pone.0312267)
Supplement: Attachment — Submitted filename: Response to Reviewers.docx [file pone.0312267.s008.docx]

***Point-by-point responses to reviewers’ and editor’s comments***

**Reviewer 1**

**Comment 1:** There are a lot Keywords, usually require about 3-8 keywords but 9 are a lot.

**Authors’ response:** Thank you very much for your vital comments. As per your comment and comment 2 from editor, we have revised the keywords to align with journal requirement.

**Comment 2:** The title is so long too. I suggest: "community health education on mothers' knowledge with obstetric risk signs, birth preparedness and complication readiness practices in southern Ethiopia: A cluster randomized controlled trial"

**Authors’ response:** Thank you, too, for this comment. We have accepted your comment and made the required revisions as per your comment.

**Comment 3:** Focus on the variables identified in the title so as not to make the writing so extensive.

**Authors’ response:** Thank you for pointing this out. This comment is also shared by reviewers 2 and 4; we have revised this section of the manuscript as per the suggestions of the reviewers.

**Reviewer 2**

**Comment 1:** The introduction could be significantly shortened, and some of the information used more in the discussion section. I would suggest the following structure; a. Paragraph describing ODS including some prevalence data b. Paragraph addressing the women’s knowledge gap of ODS c. Paragraph describing interventions and government efforts d. Paragraph describing the lack of research and objectives of this manuscript 2. It would be good for the authors to add some data on prevalence of ODS and maternal mortality particularly in Ethiopia.

**Authors’ response:** Thank you a lot for this valuable comment. We have accepted the comment and made the required revision. The introduction has been edited to be more logical and coherent by including prevalence of ODS and maternal mortality data as per your suggestion.

**Comment 2:** Adding a flow chart or diagram depicting the participant’s selection and randomization process would be helpful to follow the study design, population and randomization paragraphs a little better.

**Authors’ response**: Dear reviewer, thank you for your critical comment. What you raised is very important to improve this section of the manuscript. However, the flow chart or diagram is already published in another paper (see figure 2). We haven’t provided the figure because to avoid dual publication of the same figure that can lead to scientific misconduct. See the link to the publication: https://doi.org/10.3390/healthcare12101045.

**Comment 3:**  Study variables: I suggest the authors only focus on variables that are relevant for this manuscript.

**Authors’ response:** Thank you for pointing this out. As indicated in the comment (reviewers 1 and 3), we have revised this section of the manuscript to make it more concise as per the suggestions of the reviewers.

**Comment 4:**  I suggest moving the description of the intervention to supplementary material and including only a brief summary of it in the manuscript. I also think it should be placed before talking about the outcomes.

**Authors’ response**: Thank you for this comment, too. We have carefully revised this section to improve the readability of the manuscript as per your comment.

**Comment 5:**  Page 14 paragraph 2 talks about data collection and then goes back to randomization, this is a little confusing. I would suggest authors to mention everything that has to do with randomization in the appropriate paragraph and not coming both.

**Authors’ response**: Thank you for your vital comment and for fixing our mistake. Now we have revised it based on your suggestion.

**Comment 6:**  While the authors offer very detailed information and justifications for their model selection, I’m a little confused if their multilevel models were linear or Poisson models. It is not the standard to estimate ICC from a Poisson model and usually variance portioning is used. If fitting a logistic model then MOR is what is usually estimated as opposed to ICC If authors estimated ICC from non-linear models I would suggest that they add the formula of how they did it to the manuscript.

**Authors’ response**: Dear reviewer, thank you for your vital comment. As you correctly pointed out, it is not the standard and possible to estimate ICC from a Poisson model. However, we have provided a clear description of ICC estimate calculation using a logistic regression model in the data analysis and result section of this manuscript. To the best of our knowledge, it is possible to calculate the ICC estimate using a logistic regression model to determine whether or not a multilevel analysis is required. Besides, we have provided a stata do file that shows the formula for ICC estimation using stata software as per your request.

**Comment 7:**  Why did authors decide the cutoff of p < 0.25 to be included in multivariate models.

**Authors’ response:** Thank you again. **You raised a very critical point, but we haven’t only used the p-value cutoff point to screen candidate variables for multivariable analysis.** A p-value of less than 0.25 and other variables of known clinical and social significance were included for additional multivariable analysis for this study. Utilizing a cutoff value of 0.25 is supported by most statistics books, statisticians, and literatures. We have decided to use the cutoff of p < 0.25 and other variables of known clinical and social importance to include candidate variables into multivariate models because most statistics books, literatures, and statisticians recommend by rule of thumb. Dear reviewer, kindly see the following references:

1. Bendel RB, Afifi AA. Comparison of stopping rules in forward “stepwise” regression. Journal of the American Statistical association. 1977 Mar 1;72(357):46-53.
2. Mickey RM, Greenland S. The impact of confounder selection criteria on effect estimation. American journal of epidemiology. 1989 Jan 1;129(1):125-37.
3. Model building strategy for logistic regression: purposeful selection available from <https://atm.amegroups.org/article/view/9400/pdf>

**Comment 8:**  What was the reasoning behind the effect modification tests? Which variables were tested? I would suggest authors to elaborate more on this.

**Authors’ response:** Thank you too for this comment. The reason we conducted the effect modification tests was to assess variables that modify the effect of our intervention. We have already provided the variables that were tested for effect modification and every detail of effect modifiers in supplementary file 1.

**Comment 9:**  In general, authors provide many statistical terms and details to the methods section that makes it hard to follow. I would suggest authors simplify it and add only the important information readers would need in order to replicate this analysis. The section is very hard to follow and all the justification and details can be distracting.

**Authors’ response:** Thank you a lot for this appreciated comment. We have accepted the suggestion and done the needed revision. Now this section has been revised as per your suggestion.

**Comment 10:**  On the description of table 1, it would be important to highlight the differences between groups, given that this is an RCT and Table 1 should be used to assess balance between groups. The statistically significant differences for Mass Media access and wealth index are important and could be associated with the outcome as well so should be highlighted

**Authors’ response**: Thank you for this remark. We have highlighted all variables that significantly differ between two arms during bivariable analysis. These variables were considered as potential confounders and controlled using multivariable analysis.

**Comment 11:**  Similar to previous comment, it is important to highlight significant differences between groups, previous history of neonatal death and ODS during last pregnancy could be associated with the outcomes as well and should be highlighted as potential confounders.

**Authors’ response**: Thank you for this remark, too. As indicated in above comment 10, these variables were considered potential confounders and controlled using multivariable analysis.

**Comment 12:**  I find tables 3 and 4 a little confusing. I would separate the N(%) of good and poor knowledge into a separate table and just leave the model results. Also, it is not very clear which models the authors are showing. In the methods they refer to a sequence of four models and they only show results for 2, clarifying that would be very helpful.

**Authors’ response**: Thank you for the likely comment. This comment is also shared by reviewer 4; we have accepted comment and revised tables 3 and 4 as per suggestions of reviewers. We have provided the results of the final model (model 4) in this manuscript, but not for two models. The final model that contains both individual and community-level determinants was used to present the findings of this manuscript because it is the best fitted data as compared to the other three consecutive models (models 0, 1, and 2).

**Comment 13:**  While the information provided is good and authors do a good job of comparing their findings to other literature, the limitations section is very long and disorganized. I would strongly advise to summarize better the limitations with regards to clustered RCTs and then they can talk about limitations of analysis or other types instead of going back and forth which makes it hard to follow.

**Authors’ response:** Thank you for this comment. We have accepted your comment and did the required revisions as per your comment to make the section more concise.

**Reviewer 3**

**Comment 1**: Amanuel Yoseph and co-authors evaluated the effect of community-based health education intervention facilitated by women's groups (women's development team) on mothers' knowledge of SDG and BPCR practice in Sidama region in the southern Ethiopia. I would like to thank the authors for this excellent manuscript. A problem that affects a high proportion of women in underdeveloped countries is outlined: high maternal morbidity and mortality. But, even more importantly, the authors present evidence on effective strategies to mitigate this important public health problem. Adding to the relevance of the topic is the correct design, the careful handling of ethical aspects, a robust analysis and the writing of the manuscript.

**Authors’ response**: Thank you for your kind remark.

**Comment 2:** My only suggestion is to review the title, its length seems a bit excessive to me.

**Authors’ response:** Thank you a lot for this valuable comment. This comment is also shared by reviewer 1; we have accepted the suggestion and made the required revision as per the suggestion of both reviewers.

**Comment 3:** Additionally, I consider the comment "while another paper is focused on three skilled [...], in the "Study variables" section, unnecessary

**Authors’ response**: Thank you for the genuine and plausible comment. We have removed this unnecessary description from the manuscript as per your suggestion.

**Comment 4:** Congratulations, I really enjoyed reading the manuscript.

**Authors’ response**: Thank you very much again for your kind remark.

**Reviewer 4**

**Comment 1:** Introduction Section: From our perspective, the ideas in this section are long and repetitive. We recommend condensing sentences 2, 3, and 8. Sentence 6 could be rewritten or omitted.

**Authors’ response:** Thank you for this comment. We have accepted your comment and did the required revisions as per your comment to make the introduction section more concise.

**Comment 2:** In order to develop an educational intervention that addresses complications and reduces maternal and infant mortality rate, it is crucial to have a solid theoretical and causal framework. The causal framework is a diagram that considers the factors associated with social knowledge and establishes clear theoretical relationships between these factors. By incorporating a causal framework, the intervention can show its function and impact, highlighting the desired variable of change and the desired health outcomes. In addition, the baseline measure allows identifying should identify the variables that should be included in the analysis conceptual and ensure factor similarity between the intervention and non-intervention groups. It also allows for including relevant variables in the statistical analysis. The causal framework will justify the importance and scope of the intervention. For further help, see the following references:

**Authors’ response:** Thank you for the important comment. This study was developed based on the theoretical framework, which was well elaborated in another publication of this project. We have already provided all details of the theoretical and causal framework as per your suggestion in another publication. It was denied from this manuscript to avoid plagiarism of similar theoretical frameworks and figures from that paper. Dear reviewer, kindly see this link to the publication: https://doi.org/10.3390/healthcare12101045.

**Comment 3:** It is recommended that the participant recruitment process be described in more detail both in the text and in Figure 1.

**Authors’ response**: Thank you for this comment. Our manuscript participant recruitment process seems to contain detailed information. However, we have revised this section based on your suggestion to make it clearer for the reader.

**Comment 4:** I am confused by the way participant recruitment is described. If pregnant women were selected by visiting all households, if so, how was the response rate assessed if some households had a pregnant woman who did not want to be interviewed, or was not home because she had been hospitalized for complications of pregnancy?

**Authors’ response**: Dear reviewer, thank you for your vital comment. As you correctly pointed out, there is a possibility of refusal of study participants due to different reasons. However, we haven’t practical experienced the concern you raised because we provided detail information about the aims and significance of the study, method of selection, benefits, and harms of the study to pregnant women. Due to this, study participants actively participated in this study during data collection. Besides, our data collectors visited a single pregnant home three times to avoid non-response due to absenteeism. If women were absent from home after three consecutive visits, they were considered non-respondent. We had never experienced hospitalized women during data collection because our project data was collected after 45 days of childbirth. We only experienced non-response due to change of place, abortion, stillbirths, and mortality during our data collection, as clearly shown in Figure 1.

**Comment 5:** Other questions: How many households were visited in total?

**Authors’ response:** Thanks for this query. However, we have clearly provided the information in the respondent details part of the result. Between November and December 2022, we assessed 1,440 pregnant mothers or households for eligibility; 1,126 from 24 clusters satisfied the criteria and were recruited for the study.

**Comment 6:** What is the rate of refusal or nonparticipation?

**Authors’ response:** Thanks for this query, too. A total of 1,070 (95.02%) women were available for outcome assessment during the data collection period: 540 in the intervention (94.91%) and 530 in the control (94.13%). The study's overall response rate was 95.02%. The proportion of women lost to follow-up was comparable among both groups (4.98% in the intervention group vs. 5.87% in the control group).

**Comment 7:** During what stage of the recruitment process must women provide consent to take part? What was the community consent process? Given that the intervention was part of a major project, at what point in the study process were the groups randomized?

**Authors’ response:** Thanks for this comment. The written informed consent was received from all pregnant women who met inclusion criteria before randomization and enrollment. The community leaders approved on behalf of the community before recruitment and randomization. Randomization was conducted after successful screening, consent, and recruitment of study participants.

**Comment 8:** How do you measure the equivalence of the two groups in personal, social, and health service variables that theoretically take part in knowledge?

**Authors’ response:** Thank you for the important comment**.** We have tried to balance to arms based on all attributes like personal, social, and health service variables that theoretically take part in knowledge using stratified randomization. However, some of the covariates were unbalanced from measured sources. These confounders were controlled using multivariable analysis, but confounding from unmeasured sources cannot be ruled out. This issue was clearly discussed in the limitation part of this manuscript.

**Comment 9:** In the sample size calculation section, if a household census was conducted and pregnant women were identified, the most appropriate thing to do is to identify the statistical power or precision got when identifying knowledge in the total number of women who took part in the study. On the other hand, what were the criteria for generating the clusters?

**Authors’ response**: Thank you for the genuine and plausible comment. Because of this, we have calculated the power of the study and increased the number of clusters to gain sufficient power. Clustering is a mechanism in which related objects are bunched together. We have used already government-generated clusters, or *kebeles* (the lowest administrative unit in Ethiopia), rather than generating new clusters using a certain criteria.

**Comment 10:** After randomization: Are the factors that determine knowledge the same in both groups (intervened and non-intervened)? How do you check if randomization worked in creating kebeles?

**Authors’ response**: Thank you for this comment, too. At the design stage, we used stratified randomization using place of residence (urban and rural *kebeles*) to ensure the balance of two arms. However, the stratified randomization in this study cannot completely assure the perfect balance of factors that determine knowledge between both arms. We checked the balance of both arms during the analysis stage, and those unbalanced covariates were considered confounders. A multivariable analysis was used to control the effect of known confounders in the manuscript. However, confounding from an unmeasured source cannot be ruled out for this study, and this issue is provided in the limitation of the study part.

**Comment 11:** In the variables section of the study, when it is noted that “The respondents in the study spontaneously mentioned three questions… spontaneous knowledge was defined… they could spontaneously name two or more…” Question: During the validation of the questionnaire and in particular of the knowledge questions, was the potential information bias that could influence the results quantified?, and in this context, did they measure its potential impact on the misclassification of knowledge in the statistical analysis?

**Authors’ response**: Thank you for this important comment. What you pointed out is a possibility for this study. However, cognizant of this, we have already considered this during the study tool validation and data collection period. We have taken all important measures to minimize the effect of potential information bias by standardizing study tools, conducting pretests, providing training for data collectors and supervisors, telling the study participants about the importance of questions, and encouraging study participants to provide genuine responses. However, these efforts and measures cannot completely preclude the occurrence of information bias, and this issue is discussed as the limitation of the study part.

**Comment 12:** Regarding the measurement of conceptual constructs such as “knowledge” during the analysis of the information, does the questions have the same value to measure the dimensions of the Knowledge construct?

**Authors’ response:** Thank you for this query. Yes, we have used the measurement of conceptual constructs such as “knowledge” during the analysis of the information as provided in the definition part. Here is below the definition of knowledge question constructs that is clear and straightforward.

Maternal knowledge regarding ODS was measured using 30 questions during three phases, namely antepartum (9 questions), intrapartum (12 questions), and postpartum (9 questions). The correct answers were assigned a score of 1, while the incorrect answers were assigned a score of 0. The total knowledge scores range from 0 to 30. The study respondents who spontaneously mentioned at least 3 ODS during each phase were classified as having "good knowledge," and those who spontaneously mentioned two or fewer ODS were classified as having "poor knowledge"

**Comment 13:** In the last sentence of the same section, they show that this study is part of another larger project with another aim. It is not clear if it affected the creation of representativeness of women in the community or the recruitment and randomization process. Can you clarify?

**Authors’ response:** Thank you for this query, too**.** We have conducted similar procedures, such as the creation of representativeness of women in the community or the recruitment and randomization process for this objective and another objective, including data collection. Thus, everything is similar and does not affect the objective of this manuscript.

**Comment 14:** Regarding the sections on data collection procedures, data techniques, and ethical statements, please reduce the length of these sections and rewrite them.

**Authors’ response**: Thank you for this comment. We have cautiously and comprehensively revised these sections to improve readability and understandability, as per your comment.

**Comment 15:** How did the topics offered to improve knowledge used in the intervention emerge? What competencies should participants have at the end of the educational training?

**Authors’ response:** Thank you a lot for this valuable comment. The topic of this manuscript emerged before the initiation of this study. While we reviewing literature, we found that one research article that reported women's education status was associated with knowledge of obstetric danger signs. At that time, we imagined that if we provided community-based health education for rural illiterate women using a women's development team, it might improve the women’s’ knowledge of obstetric danger signs. As a result, we designed and provided health education for pregnant women, and it improved women’s knowledge of obstetric danger signs. This is the story of how the topic of this manuscript emerged. The study participants who have successfully completed 6 months of training graduated and obtained identification of model families in each respective interventional *kebeles* from the district health office. Now they are a model family and diffuse the information about obstetric danger signs, birth preparedness and complication readiness, and maternal health service utilization in the *kebeles* during the pregnant women forum. The topics of health education intervention emerged through a comprehensive review of different literatures and adapted to the local context by research teams. Besides, topics and contents were reviewed by health education and promotion experts from Hawassa University to assure whether or not they conveyed the intended messages for this study. The final version was developed after several revisions by taking feedback from different experts.

**Comment 16:** Were assessments conducted before the intervention? What were the assessment results at the beginning and during the training process? If so, is there a parameter at the beginning of the intervention to measure the difference in knowledge between the intervention and non-intervention groups? If so, were these controlled for in the statistical analysis?

**Authors’ response**: Thank you for the genuine and reasonable query. Yes, based on the rough assessment findings, the both arms communities were similar before implementing the intervention with parameters like wealth status, education status, age, health service availability and accessibility, place of residence, topography, distance from health facilities, level of mass media use, etc. Besides, we have tried to balance both arms using stratification randomization by place of residence (urban and rural stratum). However, most of these parameters were imbalanced between two arms during statistical analysis time. These parameters were considered as confounders and controlled during statistical analysis using multivariable analysis, while the effect of confounding from unmeasured sources of the parameters cannot be ruled out. We have already addressed this issue in the limitation of the study section of the manuscript.

**Comment 17:** Results section. The statistical analysis should begin by evaluating the difference in the knowledge indicators that allow the initial counterfactual to be evaluated and induce the final counterfactual. It is important, measure the change before and after the intervention in both groups. A knowledge index be developed to meet the stated aim.

**Authors’ response**: Thank you for this important comment. However, this is not a pre- and post-test design to measure before and after intervention change in both arms; in our cRCT design, we have recruited study participants after we assured baseline comparability between two arms and measured level of knowledge at the end of intervention, which can preclude us from measuring change.

**Comment 18:** The tables are extensive. Please include the results of the important variables.

**Authors’ response**: Thank you. Now we have revised tables to make them more concise as per your suggestions.

**Comment 19:** Explain the statistical differences that allow the identification of variables used in the adjusted statistical model.

**Authors’ response**: Thank you for your vital comment. However, we have already provided the statistical differences that allow the identification of variables used in the adjusted statistical model for this study in the statistical analysis and result section. We used a crude analysis p-value less than 0.25 cutoff point and other variables of known clinical and social significance to screen candidate variables to enter the multivariable analysis.

**Comment 20:** Tables 3 and 4, please include at the bottom of the table the variables used to adjust the final model, and show the most essential findings that allow the subsequent discussion of the results.

**Authors’ response**: Thank you for your suggestion, and we have revised the tables as per your suggestion.

**Comment 21:** Create a bar graph showing the percentage of increase in knowledge caused by the intervention that is the objective of the study.

**Authors’ response**: Thank you for your suggestion, and we have created a bar graph as per your suggestion.

**Comment 22:** In the Discussion section, it is recommended that the authors include the percentage of change in knowledge about the ODS and BPCR practices got at the end of the intervention.

**Authors’ response**: Thank you for your suggestion. However, our aim was to assess the effect of health education on knowledge of ODS and BPCR practice among pregnant women but not percentage change. We have provided the recommended information of the percentage increase in knowledge using a bar graph as per your suggestion in the result section. In this study, the percentage increase of knowledge and practice using the chi-square test in unadjusted analysis was significant between the two arms. Similarly, after adjusted for the confounders and effect of clusters, the effect of health education on the knowledge of ODS and BPCR practice among pregnant women was significant. Thus, discussing these two similar significant differences between two arms makes the discussion section repetitive and very long. This is the reason we omitted discussing the percentage increment between two arms.

**Comment 23:** An important aspect of the discussion of the study findings is the statistical analysis, which includes both intention to treat and final analysis.

**Authors’ response**: Thank you for this comment. However, our project is proposed to be conducted with the intention-to-treat analysis, and we have conducted it as per the proposal. Now we have discussed every detail as per the analysis.

**Comment 24:** The authors refer to an increased likelihood of women performing BPCR after the intervention, but do not specify which specific outcomes they are referring to.

**Authors’ response**: Thank you for this comment. However, we have clearly indicated the BPCR practice outcome. This outcome is an overall outcome calculated from five components of questions and defined as follows:

BPCR practice was measured using a questionnaire having five components as to whether or not the woman planned for her index pregnancy, such as identifying a closer proper HF for childbirth; finding and communicating an SBA; saving money; preparing material resources for childbirth and preparing for other associated costs; preparing or arranging transportation to a proper HF in case of childbirth and obstetric emergency; and identifying and fixing compatible blood group givers in case of blood requirements. If a woman prearranged at least two components out of 5, she was considered as having "well prepared" and otherwise considered "poorly prepared"

**Comment 25:** The discussion mentions potential cost and material savings, but it is unclear whether this was one of the study’s goals.

**Authors’ response:** Thanks for this query. Yes, it is the descriptive objective of this study, and we described the main BPCR practice components, like saving money and materials, in the result and discussion sections.

**Academic editor**

**Comment 1**: I currently have four reviews for your paper PONE-D-23-40070. The reviewers, who have important expertise in areas your paper covers, including health interventions and methodology, overall found the manuscript of potential interest and useful findings; however they raised important topics that need be addressed by author in the resubmission. Taking into account the reviewers' comments will improve the manuscript for acceptance. The changes should be observed mainly in terms of synthesizing the introduction, strengthening the methodology and strengthening the discussion and conclusions.

**Authors’ response**: Thank you for your kind remark.

**Comment 2:** My suggestions: follow the criteria for publications in PLOS ONE, reduce the number of keywords, and check consistency between all sections of the manuscript.

**Authors’ response:** Thank you a lot for this valuable comment. We have accepted the comment and made the required revision. The keywords have been edited to be consistency with PLOS ONE publications criteria as per your suggestion. Besides, we have checked the consistency of all section of our manuscript.

**Comment 3:** If you would like to make changes to your financial disclosure, please include your updated statement in your cover letter. Guidelines for resubmitting your figure files are available below the reviewer comments at the end of this letter.

**Authors’ response**: Thank you for this important notice**.** However, we couldn’t change our financial disclosure because we have correctly stated in online submission system. Besides, we have already followed the figures files preparation style of PLOS ONE journal.

**Comment 4:** If applicable, we recommend that you deposit your laboratory protocols in [protocols.io](http://protocols.io/) to enhance the reproducibility of your results. Protocols.io assigns your protocol its own identifier (DOI) so that it can be cited independently in the future.

**Authors’ response**: Thank you for this recommendation. This study hasn’t laboratory protocol but contains study protocol. We have already provided study protocol for journal as supplementary material.

**Journal requirements**

**Requirement 1:** Please ensure that your manuscript meets PLOS ONE's style requirements, including those for file naming.

**Authors’ response:** We have followed PLOS ONE’s style requirements, including file naming conventions, in preparing the manuscript.

**Requirement 2:** Note from Emily Chenette, Editor in Chief of PLOS ONE, and Iain Hrynaszkiewicz, Director of Open Research Solutions at PLOS: Did you know that depositing data in a repository is associated with up to a 25% citation advantage (<https://doi.org/10.1371/journal.pone.0230416>)? If you’ve not already done so, consider depositing your raw data in a repository to ensure your work is read, appreciated and cited by the largest possible audience. You’ll also earn an Accessible Data icon on your published paper if you deposit your data in any participating repository (<https://plos.org/open-science/open-data/#accessible-data>).

**Authors’ response:** Thank you very much for this vital information. Now we have provided the full data set on repository in which the manuscript is prepared to take advantage of citation as per your suggestion.

**Requirement 3:** Thank you for stating the following financial disclosure: 
 [Hawassa University and Sidama region president office].  
Please state what role the funders took in the study.  If the funders had no role, please state: "The funders had no role in study design, data collection and analysis, decision to publish, or preparation of the manuscript." If this statement is not correct you must amend it as needed.

Please respond by return e-mail so that we can amend your financial disclosure and competing interests on your behalf.

**Authors’ response:** As we also responded to the above comment (comment 3), the financial disclosure statement has been correctly stated as per your narration during first online submission in the system. All the required details are provided. However, if there is concern which makes some confusion for you due to the discrepancy in the online submission system for financial disclosure. Please kindly consider amendment of financial disclosure and competing interest on our behalf.
